# Correction: Evolution of insect olfactory receptors

**DOI:** 10.7554/eLife.05087

**Published:** 2014-10-13

**Authors:** Christine Missbach, Hany KM Dweck, Heiko Vogel, Andreas Vilcinskas, Marcus C Stensmyr, Bill S Hansson, Ewald Grosse-Wilde

Missbach C, Dweck HKM, Vogel H, Vilcinskas A, Stensmyr MC, Hansson BS, Grosse-Wilde E. 2014. Evolution of insect olfactory receptors. *eLife* **3**:e02115. doi: 10.7554/eLife.02115. Published 26 March 2014

In the published article, there were errors in the files for Figure 8—source data 1 and Figure 8—source data 2.

The amino acid sequence PsicIR76b_translated_full ORF in Figure 8—source data 1 and the nucleotide sequence PsicIR76b_partial mRNA in Figure 8—source data 2 were identical to the preceding sequences for PsicIR8a. The correct sequences for PsicIR76b were therefore missing.

The source data files have been corrected to replace the duplicates with the correct sequences. The corrections are shown with tracked changes in Supplementary files 1 and 2 (file held on figshare under doi: 10.6084/m9.figshare.1352024).

