# Supplementary material for: Evolution of insect olfactory receptors
Source: eLife. 2014 Mar 26;3:e02115. doi: 10.7554/eLife.02115 (PMC3966513; doi:10.7554/eLife.02115)
Supplement: Figure 4—source data 1. — DOI: http://dx.doi.org/10.7554/eLife.02115.010 [file elife02115s002.docx]

>LsigGR1_ putative gustatory receptor_translated full ORF

MKVDPNSEAACDTSGSNTIYSELKPALLLAHALGVIPFSPAEHGLTFTWCGLKVWHHVAGLLTFLVALCYLSWRLTYGDFENSADIILHVSITFLSASALLIGAVWFLNRKKFPELLNKWAELQALLSTSEESVDEEQKYNLNFCCANRKWYVIVTYLISVLCMGLNMAGVFVHHATYQRFPPYPTSLVSAPSNLLITVSWLFTLGYMLLTLAAVTFLEIVLVIISLNVACAYNVINNRLSAVLSSQEKDVYDEKKQAKIVRKRCCYQARKERGTSPVSTIVPSEASLKSSEDILTKAIADHETITVFLDEVNSIFGLTLLVQLVVFIVLICALSYTTLSMYNSINLLVTLVIGNNALCFLAKACLLIPCYGRISDAAHQPTVILHKILTSIWSKVKKAPCYELINTFMTRLYSSPVSLSAWGVPLTSSTVLAFLSITATYIIVLLELQNPLPDDTVKCNATIS*

>LsigGR2_ putative gustatory receptor_translated full ORF

MRWSEFKDVLRYLTGQSTMVKEISRPNTGFLIELKQSQGITSVTNEKSSEFLYLVQPPNHKGIWEAFRGILRFACICGIFPLHSIFQNDVNKLHFKICTVPMLIWLLAFIGIVTFNLATICHLFVGKPKADGDILPDGSTGLMFYFCIMISSLQLLVFASRFPTLLTKWRHGEA?LRNYGCDENLPKICWAICFIILTSGFVEHGGSIANAIWNPKENQLNETDLTSLLERYSLLSHSFIFSQIPYNPYTGTIAFIVSNYSTIVWNFLDLLLILVSFCLHRQFRKLNLSLKDDATKSWTSKEWRCFRENYVEMVEVIWEADKAISGMFLTYYIGNVFFLCMGILAQLTRVTSGIIDSIYHAWGFVHMLGRTMAVSLCASKVFEDAQEPLRAIYTCSSDTYCTEVSRLQTHITSTPIGLTGLNFFIINRGFLLGMVGAIVTYEIVLLQFTSSSK*

>LsigGR3_ putative gustatory receptor_partial_translated mRNA

IKKTADMSVGKKLQTARSMYESLVKGIYSFNRFCGPLILLAMSVSVFIGITTVFHFGTLLLTMVKNSPXPNESPLFLLTVITAFSLIIIIIVMPAEGVANAAQSVTEVLRSSQCYDLKSSEANQVQVFLASNMASPVRINACGVFDVGTGLLAPLIGNVVTYVLVLLQFQLGEDTSTTSNETSTGQPIFQSTES

>LsigGR4_ putative gustatory receptor_partial_translated mRNA

VHGESQASSGTTTATTSHLHYRSTSSRGSGQVAAFESLCSRHVLLDTLVRDMCSLLGPGLWISSLCSLTLSVVNMYFFLKEFATPHIGLLQSFTYLLMALLNSARLINLVLAASVVTKKASQPLEVLWKLVPSRMTTNLYQQVTLMSSKLLHHPLQITASDFFVLDRRLLTSIFGAFITYVVLLVQFKMSEQNEDCSNNTQWRK*

>LsigGR5_ putative gustatory receptor_partial_translated mRNA

VVLSLLGIVRLFVLSKAGDLLQDEAHHAPESILQQVSTAALGSKCEEKIHFFAVKLAGKRVGFQPAGFFNLNMELLTSIAGAVTTYLVVLIQFQVSGTEPETNSTTISAVEDFSMTTPL*

>TdomOrco1_putative coreceptor of insect ORs_translated full ORF

MPEPQKGLIALLQTHIKFLQFSGHFMLDFHSNDAPMMRWFRAMYSIMQI?ISTLHMIFCVLRILYSLSNLSKLVPVVVSTTFAIHGVIKLIYVAVRRKTFTKVLRLWDDAGTHPMFEKADEMTLQVTRYRTKRWLIISTVFYLFY?VFWTVSPFFDKDYEDIMVDNETMRVDKPRLIVGAWYPLDLTSSPGYQIAFLYQMYWAFFGPMQVHSIDILFCCMLVHASEQLKHLKKILIPLVELSSNPDGKSVTEKSKYMGLSQLSLLSEGNNYMSELPRRRQMAWSTNRIYVDEVLNRENVMNSGGGNTPMDAGGAAELIENEKARNLRSAIKYWVERHRQIMRFASDVEEMYGLALLFHILLASLTLCLLAYEASQIREMNVYSINVLGYMIQNLLHIFVFCIEGNSLIEQSSSLMRSVYDSSWYAGSEDAKVFIQIVSQQCQRPLSISGAKFFTLSFDFFGSVLGAVITYFIVLVQMK*

>TdomOrco2_putative coreceptor of insect ORs_translated full ORF

MKYIRQGLVADIYPIIRTMRFVGHYILRYYQDDGPMKRAFRAIYSVSNISLITLHFLLGAVSIMFKMNDIEGLVANAISTFFAFHAVTKMIYFAVRKKAFYETLDCWDVTNSHPMFAESNARFKMSAIRRTKDTSAICIWRMCAFSSSSVYTAIFVAPFRTIMDGNETIVVENSPLIVDAWYPWSLKDFTFFAASYFYQLYWLIFCIFQVNSIDVLFCSFLIYACEQLKHLKEIMTPLVELSAGRDPEALRKAELWPEITAIDKSASRLDGTPPPYQTA?RNRIYPETLGVDMERSLVLSDFAHLKEPMVTYSTDEANIGENVLTKKQQLYVRSAIKYWVERHKHVVRFVESVGDTYGLALLLHMLTSTITLSLLAYEATKISAFDIYAMNVIGYLLYTLLQVFLFCIFGNDLIEESLSVMKAAYECPWYNGSEEAKTFIQIVCQQCQRALSISGAKFFTVSLDLFASVLGAVVTYFMVLIQLK*

>TdomOrco3_putative coreceptor of insect ORs_translated full ORF

MLGFQSLQVCLQFNFRLLKVAGHWITEPAQDNNNRQNAALHWSYWLLLLYRVFITLITTIHVISVVAGCLKNADKFKDQPMIGAMGLFAFQALVKLVYIILKRAKIKNVLNTWNDTYTHSSFIWSRINAIESSTKSSKTVSTCLLASYVVLAIQWCLAPTSVSKDTTEEYNITTFINVTSSSKPLPFLAWFPLDFERSPIYTFIFAFQVVASLYFALIVAAFDGLFCALLSQAVNQMDHLRDSLGFLIDVCIENEPLLKDNIKSPNRTAIDNLASEIAGGLYRRYITSSSGSRIRPIELPYDQRMNKSTNLIVKSGRNKGFTHFGGDDYWENMRMSMSYCIHHHQYLIRFTDTLEELFSASMLIQFLYSTGLLCVLAFEATLIRGFDMKTLTLVVFLLVSVIQLFAICSYGNKILSESTRVTEEAYSKAWHKGSEDVRNVLQLIFQRSQRALVLSGANIFTVDLETFANVLAASFTYFMVLIQLG*

>TdomGR1_putative gustatory receptor_partial_ translated mRNA

MWFNDTTIKCFFTQSRSRNCSGRSCKEVKVKDMETVSDVFYHELKPIFVVLRITGCFPILHTAAGVFTFAKCSLLSLVTFLHYIGYLALSIYFSRSVLDLIGAKDRNFDDMVYDAIRLTYFIMPHVHLVTFLCKSKNIADYLKHWYEVETLFFKTTGKCLVLHQRRKAWLMILSTPVLVLLAGTQQHFVFAKLDTWHKLTFCYSMSLSIFVDILWTLICWSLIETCKTYTEQLRSVLSDSSGCFFVHTLRRYRGLWLQLCRLIEETGSVLCFQ

>TdomGR2_putative gustatory receptor_translated full ORF

MSENPEVSWKDRLFEDWAVRNNVYISSKKPLSFLQYFGLMPYSIENSERLVFSKGWNKWSWIFRGSLLTIFPLLCIYFVKVQYNESDIIWWSLRFQIIVGFCFMYFIPLWYLAKGVRKTRKICFHYFADEIVETYKMMSDQTIMDLLSPAKEECNMPWKAIVCFILC?IFLYIFKILISLSMVTSIFGYLYGLLAFVSFIASQPDFIYVLVFAYLCRFLYTRFDALMKKVDEAARTENVKHLFEIYRKLCDIVDYVNDTFGQYLLLSTYYNLIQLVASLFYFMYTVFTNEKTTYKTPYIIIAAATISFTIVQFINIVSHCSEVVQKVEEIRNKVLQKAFVEVADGSFAEFSLLMESRKVQFTTNGWFSLDNIFLYTVIGAVIVCLAVFLSFQIDLSNN*

>TdomGR3_putative gustatory receptor_ partial_translated mRNA

MNVYETAKRPLQVLRCFGLLPYVVENSKELVRSTGWLVYSVAFRLFVSLSVVVMLLYSCNYYILDPHKVHLELDAVGWSLICQIAISVAFIFFISIYDFCKNVTKVFGLLTEVDEIVVTFPSASFHNANPWNTLAILNICLWLVIFKTVLSIVMVVVMKSGLLLFFVLAFVSFVASFLDLICILAFYYVCLLLYQRFQVMKTELGLVVKELSTDVQGEGLFHSVTKLKTLGELYNKLCEIIDRVNATFGLYLLVSIIYNFIQVVGCVFYVVYVLFPILHMDKMWIRYGRYGLIMEMDRISNIAITAATTLLSFLIFYCVVFHCSKVMQKVKDIHNELLYSIALEDCQSRELEHQLLKFFHLLEFRKVKFT

>TdomGR4_putative gustatory receptor_translated full ORF

MCAVRMSIVSIALFIVSWTILIILTFFAIVLLIRENIYKDSILTSTGLIAWNLDLLIVSCSGLLSYGFSIKNFYKIKEMIQLMKGYEAELKIPSSKFNVSQLCVGTYVFVLVVVNVYDCLSFTNFFRDSRRLFLTFYYFLRLPPIPVDLCVYIILTKLARCFSAINSRISTEHPCYESTTIRPLQLEQDFRFVSKLSALSSNSLSAADVRQILRKHALFCQNIEDFTSLFGIPIFAAVLSCVILLIAKLYFFWVYVYHKLFDNINSVIAEGLWPLIYICRLFVITWSCEQVTEKAKRTGVLLHKIRSPTMDAETKEEIQFFSSQMLHRKIEFSACGFFTLDFTLLCSIAGAVTTYLVILIQFQTSVKDESTSTYSTASPMWNVSTTPL*

>PsicOR1_putative olfactory receptor_partial_translated mRNA

QFDVLSATLRGVRRVAEDELGLRAGTASTQELFDTELLPCVQRPSESANYHLMLDRQMKQILIHCIKHHKDVIEFVRKLDAVYNPVMLGLILYSMTMIAIASFQASESSTELGDLMKFAVLSSTCIFELYIFCTLSEDILQRSQAVGTAAFCCDWCDAGTEVKNIVKMFIVRANRP

>PsicOR2_putative olfactory receptor_partial_translated mRNA

IFSPVIFCEVTNCLAIICQAGFLVLVKKGSIAEFLKYFGYLVVTLFELLLYCWYGNEVIYQSSMVQSAAYDSAWLNSSPQFHSTVQMVMLRSQTKLALTAGNLHTMSLSTFTAVSRLQFTVKPDRTSRTVTCSATN*

>PsicOR3_putative olfactory receptor_partial_translated mRNA

ESEMMKGFCQQHQEMLRCAQELVDLLRNWLFSHYLVASLLVCSLCYRLQVMEDMRDLFTQVSHLTVVMFRLFTLCYFSSELTHQSLRVADAAYSCAWHHLGADAKRCLVMIACRAQRPVVMKTGHFGDMSLSTFSALLQAAYSYFNIMNDFG*

>PsicOR4_putative olfactory receptor_partial_translated mRNA

RTGSSSEDRSSDIYGKLEGDNSSELEKNLIDLVKLYQDILCNAQALADLLSPLMFTHYLTSSINVCLVAYETAMTDEISQMVAGVSHLMMLMGRIFLLSYFSSEIMDQSMNVGQMAYSCNWLSCSKDFKRMLMMMMCRAEKPVVMRTGWFGILSMETFAKLMQTAYTYYTLLKKLNI*

>PsicOR5_putative olfactory receptor_partial_translated mRNA

KYFIYLYLIAKQWCYCFLLMAGTLCYMLLPLTGTSGRSQMPLTTYHLLDRADPASFAVEYLLQVCSMFAANAIIVPFDLFEFFAMKYCAHQFQAIAVLLSIADDRKARPRDHGSKVGSEPDIAPAPSPGGDGLDETWHSLIRYHQSILENAERLANLFSPLLFADYVTVSIA

>PsicOR6_putative olfactory receptor_partial_translated mRNA

MDVFSNALFVQFLTVSVSVCFCSYRIIQLPPSLKMVSIIAYMFCIIGQLGIICWYADDLSDKCLRVGEVAFSSRWYLMPASFKFCILHTITRAQKPVTFKAGYFGVISMQLFAGLLQTSYSYFAVLKQAQLQ*

>PsicOR7_putative olfactory receptor_translated full ORF

MKKHAGGIMTLPMTLLGVFGLWHGDGRWAMSVGRFLRCLTLLLSASFQLSLLRPYFHFFDDPVMAFRALGWHSTIVPTYIMAFSLLANKDKYVKIMSRKTADEDNEETGQIFSAALVQGRKLTIVYLCLGTVYTLSTWAVPLVMFHIKPSQTRVPYDIWFPVPASDPTGLVAQYVFQSTGIELSCYMVIVCDMFFISMMLKMIGDFKSLNCALMQLKTKDVTRPRLIFKLWKNFTFQGSSIGDISSLQMAHKEMDTKLAALIQYHQDLLRDVNELRQLMDTFLFVLYLLGSGNLCALAYEVSKVSGFNHQMTSAGSYLMGMVFRLGVYCYYGSEITNESLKLGESLYFSDWLSASMNFKRCCVNVIARSHRPFRFTAGKFGTLSLDAYSKIINTAYSYFMLLRKMQTTGI*

>PsicOR8_putative olfactory receptor_translated full ORF

MSSPEARFYYLGRIWYYLGLDIFDDGPGRKVTGWVYANYFCFIYCIVAYSSYVYSIVIADELEEHVHDIAILFTMSYMFFVWISATLRRKDYAEIILYLSRDVCRQYVHADVGDEIVSKAVRTERSIAKGITIWFCTSAMSQTLAPFFTMHVYYEGGTAFVNASSPTNNWTPFDRTILWQFLADYFFASVLVLIGTYISIATTIYLSIIMLHAASNFEMLNAAIESLKFENVEEGSIDHTDSQNNVCDSLPSYCSDSVIQQEVRGEAFDLKVSSVGRKESELMSRLGHRENTKNNSNCVGENAETQLYELVALHKTLKSVVAKMNTLMTPVLVLYFLGLAMNFTVLLYEIITMFAGYDSNFWMYVFHIGADFFRFGLCCFFGNMLTEKCEETCNSLYATEWYTLTPKVMKVCGIMLAVCQQRVVIGAGILGDLTMPTFAAVLSASYKYLNVLANLKTEND*

>PsicOR9_putative olfactory receptor_translated full ORF

MARPLTTRFSYVGNMWYYIGLDIFGNNDRKTRIRAFFNYFLFTYAVLGYSTFCHPLIEMVALEEQIHDIAILLTISYMFFVWISVTFYRKEYAEIIVYLMQDTCKSYITDSAAKKIINKAIGMERMVARGIQGLFLTSAMSQIVFPFFTIQVYEEGNRTVVNSSLPVHNWTPFDRSIPWQFVIDNMFLTFLVLTATYVSIATTIYFIIIIIHAASNFELLNAAISSLKAEVLRESGGECFQNANYEDSDGEPETLESVDVSPSLEDFQNTTGLKLSYAEDKDFQILFHPRSRDRMYRDLSYSLTAEAKLNELVGIHQALMSVVNKMNDMMTPVLFLYLLGLALNFTVLLYEIVTMFAGFEAGFWMCVLHIGADLFRFGLCCYYGDILTTQIEDTYQALYDTDWYKFAPNFKKAVNVMMAVCQRRVVISAGLLGDLTLETFAAVLNTSYTYLNILANLRESE*

>PsicOR10_putative olfactory receptor_partial_translated mRNA

RTTVNPIIQMEEKLSKLIEVHQAILGTVDDMNFVMGPVVFLLYVSVTINLCVLIFQAVVALEEDTRAFVMNASQLTFVMLRLGVYCYLGNALTSQAEETYQIVYGTSWYNFSGRFKSTIYIMMARA

>PsicOR11_putative olfactory receptor_partial_translated mRNA

VQFVFDRICMATVIACLMYLSLIANCFYNSLLINGASHFELLNACLLTLVTQDDGDIFSSCETGEESNEELPDLAEGTYGAVDDQTYNLPNTLKGSFHYGRKEIVSGAISPGVNREVGRTTVNPTIQMEEKLSKLIEVHQAILRTVDDMNFVMGPVVF????????????TTFLMITTQLIFVLLRVGVYCYLGNTLTTQAEETYQIVYGTSWYNFSGRFKSTIYIMMARAQRRVAISSTMVGELSMEAYADILNKTYTYFTILKNMRE*

>PsicOrco_putative coreceptor of insect ORs_translated full ORF

MQKMKVTGLVADLWTHIRVLQISGHWLLDINKSSSMMWNYLRTAGTMMQSLLLVMNYVFMIINCTQQTADSDEFTSNVATILFFSHCVFKLFYCAMRRRKFYRTLNSWNNTNSHPLFAESSARHHANATSSMKRLLIIILSCTLASAFAWITITFFGDSVIHVKDPENDNNTLIEEVPRLMIRAWYPWDTSNGFMYVVTFVYQMFWLVSMLVLCNLLDTLFCCWLIYGCEELIHLKEIMKPLMEISHSMDAIMPQTADLFQAASSSSHAALLSASTDGLDSNIRSIYNSSSEYSGLRHGIGTLATVQGSSMGPNGLSKKQELFIRSAIKYWVERHKQVVRYVNDISDTYGGALLAHMLISTVELTLLAYLATTITGLNPRGLCIIGYVIYSFGQVFQFCYYGNQLIDESSSVLEAAYSCRWYDGTEEAKAFVQIICQQCQKSMTISGAKFFTVSLDLFASILGAVVTYFLLLIQLN*

>PsicGR1_putative gustatory receptor_partial_translated mRNA

MNAHQVIAPSGGVLCAVPAGVDTVPGCGDQVAAAGTTVAGSGTSDSTPGHTRGLGRQSEGLHHNHSGCLHRGAHTGAHEDGNHGH?GSLSEWLAYYFHAEYPQVFDHFAYSAWAGILVAAIDLFAMYVWNYSDMFII

>PsicGR2_putative gustatory receptor_translated_full ORF

MKLASRVDDLRWAIKPAFLTAKLLGTAPLTATDDEDNGGGYRVSWPWLVFSLLLHSAAAGASVSCAVDGYLSTPEGSVARRFFVIGALLRACRDTTLVAASLGYRWRRCEDVLSEVADVDQEFPQCARPSRGRRAFASQVAQVSVMHLATAAFCAAYGGLSAHPSPLEAFSVAVFCEVQWAHVLEFCGVLQVLESRFTALNEQAASLERCLLAHDGKVAVAPQLCRLMHFHSRLLRLASSVNRKYQLQVFVYAVLYVLFLTLHCSTVMGAMLCQDRASGDDQGRAVICALYMSFTFIWQMVMTANLGRSLSVKGDELLMTVARMELVKGLDQECTEKVRLFCRHLSVRKVVLSAYGFFAVDNRALASAVKTGVTYLVLLVHLQKY*

>PsicGR3_putative gustatory receptor_partial_translated mRNA

SSSIVVTCSSYVAFVFSVYHSQSSPVLYAVLCLWGGVHLMRLWITTHACKATAREANRAAVVITRLLTGTNDSTHTNQLQRYSLQLLRRKVQFTASGLFPLDHTSLHNIVGTITTYILIL

**Additional contigs**

> Tdom_38756_putative gustatory receptor_partial_translated mRNA

KEQANNTSSIVEKLLHGNTKAESSSELRHFSRQLLHRKKVEVTACGIFTIDLKLLHSIASAVTLYLIILIQFDIQAKHHVIIGETANDEKTASPSTSSTTMTIQPDISFFNTSS*

>Tdom_61232_putative gustatory receptor_partial_translated mRNA

RICESTSNEADRTGVLVHRLLRRDMHPETKEELQLFASQLLHKKAEFTACGLFSINLSLLHSVVAAVTTY

>Tdom_87570_putative gustatory receptor_partial_translated mRNA

IMDILTLFILTIPSVSTMNKSNEIGILVHKLQDGKISTPAREKLQLFISQVSYRKVMFTAYGFFPVNFTLLT

>Psic_22013_predicted olfactory receptor_partial_translated mRNA

VRANRPVRLSAGGFKTMSLEAYAALMNSSYSFFAMLKQLHH*

>Psic_25058_predicted olfactory receptor_partial_translated mRNA

SVTINLCVLIFQAVIALEDDTTAFVMIASQLTFVLLRLGVYCYLGNALTTQAEETYHIVYGTSWYNFSGRFKSTINIMMARAQRHVTISSPMVGELSMEAYADMLNKTY

>Psic_2682_predicted olfactory receptor_partial_translated mRNA

VITASKFGVLSLETFSGLLRTAYSYYTLLKKFSSS*

>Psic_31622_predicted olfactory receptor_partial_translated mRNA

PGVSLPGGSADCRQEVLNSHMFQYLRTCVQHHIEIIKFVKKLDDVFNPVMLVQILYSVFDIAVTGLQATESSADFGSLIKLLALAIALFVELYLFC

>Psic_31622_predicted olfactory receptor_partial_translated mRNA

PGVSLPGGSADCRQEVLNSHMFQYLRTCVQHHIEIIKFVKKLDDVFNPVMLVQILYSVFDIAVTGLQATESSADFGSLIKLLALAIALFVELYLFC

>Psic_31623_predicted olfactory receptor_partial_translated mRNA

KTSRHVRAVTLALSFLIFLIFLSWTASPFITAGFSVTDPTGLGPHPFMSAFFFDYQSSPTYEVIYVFQTVSLFSYCLSVSSFDTVCVAFFMQVGAQFRILLESLKRLQEVAKESLA

>Psic_31984_predicted olfactory receptor_partial_translated mRNA

LTGRNRTNKLEEEESEHFSKTVYGNNERSVRMQVSRKEDKIAVVLRKLAAYHQEALRVANVLGEFLSTIFFFQYAVPSLKLCMLLYQFNRTEELFQEIHIACFLGSTTVRIFIMNFFPTRLTSKSVSLSDELGAISWYNYSPEAKR

>Psic_44668_predicted olfactory receptor_partial_translated mRNA

GDVCTSLRIVLQHCGRPLRLTAMGVVPLSSETFESVAKTAVSYFMVLWKLHTEGDDTGA*

>Psic_52266_predicted olfactory receptor_partial_translated mRNA

TEFGVELQKLYSPMWVVQFLCSLLILCFTAFVGIVVSLSVLPYVLLVGYASGNLVMLYLLCHCGDRLQEASVLVSREAHAEPWYLCGG

>Psic_69815_predicted olfactory receptor_partial_translated mRNA

EIQWYECSSIFKKTTLMILINSKVPIVLKAGPFYTVTLESFLKVVKGAYTYFTIMRQMQQ*

>Psic71173_predicted olfactory receptor_partial_translated mRNA

LVWVLGPVVMGSLEPDAPRVLPLLTWYPYDFRSSPAVYWSTYAFQVFFCWLVNSWFSALTLVYIFLVIGACSRFEMVSELFRMSGEIAQDMSKVQIADDFLDDSVMKESVRRSNKKIFSPDTEWNLCSGRRITQEFIKTETLNRMDANMKKIWVDCIIYHEKAIR

>Psic_7597_predicted olfactory receptor_partial_translated mRNA

ALCLTVYNAVMDDNAAMKLRNILFANVIASVVFLKWYFCSQLSAQSQAFGDAAYSCRWYEFNSRAKVIVAVMIMRAQRPVQMRAGSFAVVSLELYTKMVNAAY

>Psic_78369_predicted olfactory receptor_partial_translated mRNA

QRPAQLWAGHFAVMSLETFAALNIGFTLDWKNYAVGVLSTNLFYQLQYLLPYNATELRQPPGVSSRSRRQS

>Psic_82237_predicted olfactory receptor_partial_translated mRNA

AISSETETCDGVTTEFDIELEVRGKDETAQLWRLLAAYHQEALRNANNLCELLRTLLFVHYVTNSLIICMLLY

>Psic_61824_predicted olfactory receptor_partial_translated mRNA

PVFADSSNLQQERPHPLIAAYFFDHRRSPIYELVYLLQSASLLLFVPSVCGFDTTAVALLLHISAQFRVLLASLRSVR

>Psic_31984_predicted olfactory receptor_partial_translated mRNA

AFQVLSLLLLIFSVLTFDTFCMALVLHASAQFRVLSMTLRNLREVAVGLKQGDGTSDRRTSATTTRQDGGVTSTGAACDALQLSKEMRQS

>Psic_39262_predicted olfactory receptor_partial_translated mRNA

MQVSRKEDKIAVVLRKLAAYHQEALRVANVLGEFLSTIFFFQYAVPSLKLCMLLYQFNRTEELFQEIHIACFLGSTTVRIFIMNFFPTRLTSKSVSLSDELGAISWYNYSPEAKR

>Psic_33458_predicted olfactory receptor_partial_translated mRNA

CVAESLHMWLARRRFHRLVALWEAAGEFDVAHGRELSKVRLVSVCIAVTTVTPAIVYCLMPVIPGIREVLGRRHSVPAWFFFDTASSPGFEASVVFQSAAAALTVARIAAFDCFFLSLAGLHLARLGGLRRDLKAVFADLGPAADVTACLQSWIVRHQRATEFGVE

>Psic_10375_predicted gustatory receptor_partial_translated mRNA

SIFSTPIEAANTAVIVHKLINQTENRIIKNELKLFSLQLLHRKVQFSACGFFGINSGFVTSVVGVVATYVAILIQFRQSQDQQPSCCTPSQNVTY*

>Psic_33301_predicted gustatory receptor_partial_translated mRNA

VKPEEFWQQMREDYNSLSWLCLSIDKTISKLVLLSFTSNVYFICLQLYNTIRPMKKVEDIVYFYYSFGFLLLRSMLVAISAAAVNDESKEPKLVLLSVPSRSFSME

>Psic_40564_predicted gustatory receptor_partial_translated mRNA

GVVKQPEWALPLKKLRTTRPAAAMQAHARQDAAGSFHRAARPMLLMGRALSVLPLQGLLAPNASALRFSWCSLGMVYTLAVLTSCCFMLWMSVRKIVVHSWNLEDSFIA
