# Supplementary material for: Evolution of insect olfactory receptors
Source: eLife. 2014 Mar 26;3:e02115. doi: 10.7554/eLife.02115 (PMC3966513; doi:10.7554/eLife.02115)
Supplement: Figure 4—source data 2. — DOI: http://dx.doi.org/10.7554/eLife.02115.011 [file elife02115s003.docx]

>LsigGR1_putative gustatory receptor_partial mRNA

CGACGAGTGAGGATCTATCTTGGTAGTGGTGCTTGATATACGCAGTGAGTGTTTGTTAGTTCAGCAAGAAGTTACTACACAGATTTTACGCCATGAAGGTCGACCCTAACTCCGAGGCAGCGTGCGACACGTCTGGATCAAACACTATATATTCTGAGTTAAAACCAGCGCTGTTATTAGCCCACGCTTTGGGAGTGATACCTTTTTCACCAGCGGAACATGGCTTAACATTTACATGGTGTGGTTTAAAAGTTTGGCATCACGTTGCGGGGTTATTAACCTTTCTAGTTGCACTTTGCTACCTCAGTTGGAGACTTACGTACGGCGATTTTGAAAACTCTGCGGATATTATTCTTCACGTTTCCATTACGTTTCTGTCAGCGTCAGCCCTGCTGATAGGTGCAGTGTGGTTCTTGAACCGAAAGAAGTTCCCGGAATTGCTGAATAAGTGGGCAGAGTTGCAAGCATTGCTGAGTACATCCGAAGAATCTGTCGATGAGGAACAAAAGTATAACCTTAATTTTTGTTGTGCAAACAGAAAATGGTATGTAATTGTTACTTATTTAATATCTGTACTCTGCATGGGACTAAACATGGCTGGCGTTTTTGTGCACCATGCTACGTACCAAAGGTTTCCACCGTACCCAACATCACTGGTCTCTGCACCATCAAATTTATTGATAACTGTATCCTGGCTGTTTACTCTTGGTTATATGCTACTAACTCTAGCTGCAGTTACGTTTTTGGAAATCGTATTGGTAATTATATCTCTTAACGTGGCTTGTGCATACAATGTAATTAACAATAGGCTGAGTGCTGTACTCTCAAGCCAAGAAAAGGATGTATATGATGAGAAGAAACAAGCAAAGATTGTCAGGAAAAGATGCTGTTATCAAGCAAGAAAAGAACGAGGTACATCACCTGTTAGTACAATCGTGCCTAGCGAAGCATCTTTGAAGAGCTCGGAAGATATCCTAACCAAAGCTATCGCAGATCATGAGACCATCACCGTTTTCCTGGATGAAGTCAATAGCATCTTTGGTTTGACGCTTTTGGTCCAACTCGTTGTGTTCATAGTACTCATTTGTGCTCTCTCTTACACCACTCTGAGTATGTACAACAGTATCAACCTCTTAGTCACCCTGGTGATAGGCAACAATGCCCTATGTTTCCTTGCCAAGGCTTGCCTTTTAATCCCGTGCTACGGCCGTATCAGTGACGCTGCCCATCAGCCAACTGTAATTTTACACAAAATTCTTACCTCAATTTGGTCAAAGGTCAAGAAAGCACCATGCTATGAGCTTATAAACACTTTCATGACACGTCTGTATTCATCACCTGTGTCACTTTCTGCCTGGGGAGTACCTCTTACAAGCTCAACCGTCCTGGCTTTCCTCAGTATAACCGCAACATACATCATTGTTCTTCTTGAACTACAAAATCCACTGCCGGATGACACCGTAAAATGCAACGCTACAATCTCTTAAATATTAAGTCGTGGAACTGGAAGCCGATGATCGTGTTTAGCAGTGGTTGCTGGAGCGCCGGAACTCTTGAGGAGAGAGAACAGGAACTAGACGACTTCCGGTTACGGGTCACGTGGTCTTCCGGCCGGTCAACAACAAGCGGCGTCCAGCTGTACTCATAAGACACACACACCTTTATTTAATTTCTATTGCGCCTATAATTTAGCACATTCTTCCTACAACACAGATATGTACTTTCATATATCAGAATATCAGCCTCAATAACTCGTTATGTAATTAAAGCTCAATATTTTTAAGCAAATGTTTTTTATCAGTGTGACGTATTTGCTGGTCGGTATTTCATATTTTATTAAAAATGTTAGTGTTTGAAGTAAGAGATTATATTAAGTGGATAAGTCACGTGATATGTGAACTAACGTACTGTATGTACTGTATCAGCGTGTTCATGTATTTCCAAGGTGAGTTGTGGAATTGACAAATGACAATGGTAAAAAACGTGTGATACAATTGTGATAACTTTTTAATTTTGTTATATTCACTTGTTTACTGGTACCATTAGAATTTTTAGATGTGTAATACCATGAAATTAACTCTGTAGATATAGACATTTAAATATTTCAGTAAGTGTTTCTATAGTAAGCCTGCTGTTTACTTTTTTAATTTTTCTTAAAGACATGATATATAGGTGCAGATTGTAGACTCAAAATATAAAATGATTTAGCAATACATAAAACTCAGTAAAAGTGCGTAATTAACAGGTTTAAATAATCCAGAAACGTGTATTTATTGGCTGCTGTATTACAAAATTAATATTTAATTAAGTGTATTTTCAAATACTAAAAATATTAAAACTTCGGTCCTATTTTAATACAGATATTTATAAACATCCTTCACATATAAAACATATTTATATACACACTCACAATAGGTGACTCATAAAATGAGTAAAATTTTGAATTATTTATATAAATAGTAAATATAAATTGTAATCCTCAAATCACAATAATTAAGTTAAAATATTCAATCTACAAATTGTTTTCTTGTTTACCAACAATCTCTTTTAATCTGTCTTGAATTAAAATTTTTTGGTTTAAAGACTTGAAGAATTTTGTTATGTCTGTATTCAGGTTATATGTTGTGTTAGTCTGTAATATACAAATTCAATACATTTATAATAAATACATGACTCAAAATGCTATTGATTCCGTTGACGTTACAATTCATAAAAACCATCCGTACTTTCAACACAATATGTCGCAAATATCTGTGTTTATATGGTGTATAATGTACTTTCTATAAAAACCCCCAGAATATGTACATGAGTTTGTTACAATTGAAACTGTTTTAAAACAATTCTTTCCAAATATAAATTGTTTATCAATGATTGTTTACTATTAATAAGCTATCATAGAATCCCACGGTATTTAGTGGTAAGTGGCACTCTAAATTATTTTAAGCAGTCGCATTTAAAGATTGTAAATAAAGATGATACACATTTTATATAGAGATGTAGAGTAGAGTCTTGCTAATCCGGACACCCCTAATCCGGACATCCGGTTAATTCGGACGGACCTTAAAAAAAAGCGTACATGAATTTTAAAACATACACTGTACTATTTCCAAAGCTTACAAATACACTTTTAAAAAATGTTATTACCTTTTCAGAGCGTGTTTACAAGGTTTCAATCAGTAAAATGTTTTGTTTTTTATCCGTAAATCAATTCGTAGGTTAAAAATGTAGGCCTACGTTGTAGCATTAAGACTGAAAGTCATTTTTAGGACAAATATCGATTTTAATAGTGTTACCCATCTAAAACTTGTTTTACCGACATTTTAAAGCAAATTCCGTTTAATCCGGACTTCCAGATGATCCGGACGGAATCCGGTCCCATCAAGTCCGGATTAGCGAGACTCTACTGTA

>LsigGR2_putative gustatory receptor_partial mRNA

CATGGGGATTCGACGGAAATGTGTATTGAGAAGGATACTCATTTAGCGTGTGTACTTGCTTCAAACATCTATGTAACTATAGGAGTTCGTTTGTGAGAATGAGATGGTCGGAATTTAAGGATGTACTGAGATATTTAACAGGCCAGAGCACAATGGTAAAGGAAATATCCAGGCCTAACACTGGTTTTCTCATCGAGTTAAAACAATCGCAGGGAATTACCAGTGTTACAAACGAGAAGAGTTCAGAGTTCCTATATCTTGTACAACCCCCGAATCACAAAGGCATCTGGGAAGCATTTCGGGGTATACTTCGCTTTGCATGTATTTGCGGCATATTCCCTTTACATTCAATTTTCCAAAATGACGTCAACAAATTACATTTCAAAATATGTACCGTCCCGATGTTGATTTGGCTTCTGGCATTCATAGGCATTGTCACCTTTAACCTCGCTACAATATGCCATTTGTTTGTTGGAAAACCAAAAGCCGACGGCGATATCCTACCTGATGGAAGTACAGGGCTGATGTTCTACTTTTGTATCATGATCTCGTCCTTACAACTTTTGGTTTTCGCCTCACGATTTCCTACATTGCTGACCAAGTGGCGCCACGGTGAGGCACAKCTGAGGAATTACGGATGTGACGAAAACTTACCTAAGATCTGTTGGGCCATCTGCTTCATAATTCTTACCTCGGGTTTTGTTGAACACGGCGGAAGTATTGCTAACGCCATCTGGAATCCAAAGGAAAACCAGTTGAATGAAACGGATTTGACTTCGCTTCTGGAAAGATACAGTCTGCTTTCCCACAGCTTCATTTTCAGCCAGATTCCGTACAACCCGTATACTGGAACAATTGCGTTCATTGTTAGCAATTACAGCACAATTGTCTGGAATTTTCTAGATCTACTTTTGATTTTAGTCAGCTTCTGCCTTCACCGTCAATTTCGGAAACTGAATTTGTCACTCAAGGACGACGCGACCAAGAGTTGGACGTCAAAAGAGTGGAGATGTTTTCGTGAAAACTACGTCGAAATGGTGGAGGTGATTTGGGAGGCAGACAAAGCTATATCGGGGATGTTTTTGACGTACTATATTGGGAACGTCTTCTTTCTTTGCATGGGAATCTTGGCACAACTCACGAGAGTAACCTCGGGTATTATCGACTCAATTTACCATGCCTGGGGCTTTGTTCACATGTTAGGCCGTACAATGGCTGTATCTTTGTGCGCGTCAAAAGTGTTCGAAGATGCGCAAGAACCTCTGCGGGCAATTTATACATGTTCCTCGGACACTTATTGCACGGAAGTGTCTCGACTGCAGACCCACATCACATCAACGCCAATTGGACTCACAGGACTGAATTTCTTCATAATAAATAGAGGATTTCTCCTTGGCATGGTGGGCGCCATTGTTACTTACGAGATTGTGCTACTCCAATTTACTTCCTCATCGAAATAAWTATGAAAATATTAACCTACATTGTTYRATACTGAACCTATACGTCTTTGCATCTAACTACTGTGTTTAGGTTCATTTATTTCATTTATTGATTTACCATCATCGTCTTTACCACAATTGTTACGCCAGTACTGTAWTGTTGAAAATAAAACAGTACAACAGAATAAAAACATTAAATTAAGAGYCAAACTYAAGTACATAAAAAGTTTACATATATTATTTTTTCTTAAAATAAATTTGATCACTTTGTATAAACAAAAAGTGTTTCACAATTGTCCTGCAGAATTACATTTACCAAATAACACTTCAAAAGGAAATATAATAAAAATATTATGAAACAAA

>LsigGR3_putative gustatory receptor_partial mRNA

CATGGGATTGGCGTGATACAAAAATGGTGAGCTAATCGTATTGAAAAAGAGGACTATAATACAATAAATCAAGAAAACTGCAGACATGTCAGTTGGGAAGAAACTGCAGACAGCACGTAGCATGTATGAGTCTTTGGTCAAAGGGATATACTCTTTCAACAGATTCTGTGGACCCCTTATACTTCTCGCCATGTCTGTTTCTGTGTTCATAGGAATAACGACTGTGTTTCATTTTGGCACTTTGCTATTAACGATGGTGAAGAACTCTCCCGGTCCAAACGAATCTCCACTCTTTTTACTCACTGTAATAACGGCTTTCAGCTTAATTATCATTATCATTGTCATGCCAGCAGAGGGTGTTGCAAATGCTGCGCAGTCTGTTACTGAAGTATTAAGATCATCTCAATGCTACGATTTGAAATCCTCGGAAGCAAACCAGGTTCAGGTATTTCTGGCTTCAAATRTGGCTTCCCCAGTGCGAATCAACGCGTGTGGAGTATTTGATGTTGGGACTGGTCTTTTGGCACCGCTAATTGGAAATGTGGTGACATATGTTTTGGTCTTACTTCAGTTTCAACTTGGAGAAGACACATCTACAACATCAAACGAAACTTCCACTGGACAGCCAATTTTCCAATCTACTGAATCG

>LsigGR4_putative gustatory receptor_partial mRNA

CATGGGGAGTCGCAGGCTAGTTCTGGAACAACAACGGCGACCACTTCACATCTGCACTATCGGAGTACATCAAGTCGTGGATCCGGCCAGGTGGCAGCATTCGAGTCTCTGTGCTCACGCCATGTGCTCCTTGATACTTTGGTTCGTGACATGTGCTCGCTCCTAGGACCAGGCCTTTGGATTAGTTCGTTGTGTTCTTTAACTCTCTCCGTTGTGAATATGTATTTCTTCTTAAAAGAATTTGCTACTCCGCATATTGGACTCTTGCAGAGCTTTACGTACCTATTAATGGCATTGCTAAACTCAGCTCGCTTGATCAACTTGGTGTTAGCAGCAAGTGTTGTCACCAAAAAGGCATCTCAACCTTTGGAAGTACTTTGGAAACTTGTACCTTCAAGAATGACTACTAACCTTTATCAACAGGTGACGCTGATGTCCTCTAAGCTGTTACATCATCCGTTACAAATCACTGCATCTGACTTCTTTGTCTTGGATCGACGGCTTTTGACTTCGATATTCGGAGCTTTCATTACCTACGTTGTTCTCTTAGTACAATTCAAGATGAGTGAACAGAACGAAGACTGCAGTAACAACACCCAGTGGAGGAAATGAGAACTACAGTATTGAAATCATGTTGCCAATGTATATATCTGTATGACTGAGAATGGGAATTACAAATTGCCATTATATAATTTATTTTCAATAAAAATCATAACCATCTCAAAAAAAAAAAAAAAAAAAAAAAAAAA

>LsigGR5_putative gustatory receptor_partial mRNA

CTGTTGTTTTGTCTCTGCTTGGGATCGTTAGACTCTTCGTTCTATCGAAAGCAGGAGACCTTCTACAAGATGAGGCCCACCATGCCCCTGAATCCATTTTGCAGCAGGTGTCTACTGCTGCATTGGGAAGTAAATGTGAAGAAAAGATTCACTTCTTTGCTGTTAAACTGGCAGGAAAAAGAGTTGGCTTCCAACCAGCAGGATTTTTCAACCTTAATATGGAGCTTCTAACATCGATTGCTGGAGCTGTAACAACTTATCTGGTGGTCCTTATCCAATTCCAAGTGTCTGGAACAGAACCTGAAACAAACAGCACTACAATATCAGCAGTGGAGGATTTCTCAATGACAACACCTCTGTAATTCACAATCACTGATTGTGATTATTCGATATGGAAAACAATCTATCCTAACCAGTACGAAGTGTTGCATCTACAATTTATAAATACTCAATAAGGCTTACATAAGGACATTATACAGTATTTGATGTACCTAGA

>TdomOrco1_putative coreceptor of insect ORs_partial mRNA

ACATGGGGGCCATTGTTGAGGTTACAGATAAATTCATCGAGTAAACGAGACTCTAGTAGGAAATAACGCTGAAATTGCTCCAGAAATTGTGGTAAAGAAACTATAWTAAGGATGCCGGAACCTCAAAAGGGGCTGATAGCCCTGCTACAAACTCACATAAAATTTCTACAGTTTTCAGGGCATTTTATGTTAGATTTTCACTCTAACGATGCACCCATGATGCGTTGGTTTCGAGCWATGTACTCCATAATGCAAATTMTCATTTCTACCTTGCACATGATTTTCTGTGTATTGAGAATTCTGTACAGYCTGTCTAAYTTGTCCAAACTTGTTCCTGTAGTTGTATCGACTACGTTTGCCATTCACGGCGTCATCAAATTAATATACGTAGCCGTTAGAAGAAAGACATTTACCAAAGTATTAAGATTATGGGACGATGCAGGAACGCATCCAATGTTTGAAAAAGCTGACGAAATGACGTTGCAAGTTACACGATATAGAACGAAGAGATGGCTAATCATTTCGACAGTATTTTATTTATTCTATASTGTCTTCTGGACGGTATCTCCGTTCTTTGATAAGGATTACGAAGATATTATGGTTGATAACGAAACAATGAGGGTCGACAAGCCTCGTCTTATCGTGGGTGCTTGGTACCCATTAGATCTAACCAGTTCGCCTGGTTATCAAATTGCCTTCTTGTATCAGATGTACTGGGCATTTTTCGGTCCAATGCAAGTGCATAGCATCGATATATTGTTCTGTTGTATGCTAGTACACGCAAGTGAACAACTGAAACATCTTAAAAAGATTCTGATTCCTCTGGTTGAACTGAGTTCGAATCCAGATGGTAAGAGTGTAACCGAAAAGTCAAAATATATGGGATTATCTCAGCTCAGTCTCCTAAGTGAAGGCAATAATTATATGTCTGAATTACCTAGAAGACGCCAAATGGCTTGGTCTACSAACAGAATATATGTGGACGAGGTTCTTAATCGAGAAAATGTGATGAACAGTGGTGGAGGAAATACACCGATGGATGCWGGSGGTGCTGCGGAGTTAATTGAAAATGAGAAAGCAAGAAATCTTCGTTCAGCGATAAAGTACTGGGTGGAGCGACATAGGCAAATTATGAGGTTTGCTTCAGACGTTGAGGAGATGTATGGTTTGGCATTGTTATTCCATATTCTGTTGGCATCATTAACCCTRTGTCTTCTAGCGTACGAAGCTAGTCAGATTAGAGAAATGAATGTTTATTCTATCAATGTGTTGGGATATATGATACAGAACCTTCTTCACATCTTTGTTTTTTGCATCGAGGGAAATAGCCTTATAGAACAGAGTTCTTCACTTATGAGGTCTGTGTACGACAGTTCATGGTATGCCGGGTCAGAAGATGCCAAGGTCTTCATTCAAATAGTATCTCAGCAATGTCAACGACCACTAAGCATATCTGGAGCCAAATTCTTCACTTTGTCCTTTGATTTCTTTGGATCGGTCCTGGGTGCTGTAATTACGTACTTCATTGTCCTCGTCCAGATGAAATAA

>TdomOrco2_putative coreceptor of insect ORs_partial mRNA

ACATGGGGAGTTAATTTTTATAAGTGTTTTGTGGTTTCTTAAGTTGACCGAAAAGATTAATTTGTGCAGACAATATRATAATTAAATTTTTAATAATTATTTTACATATATGAATTAATATTCAAGAACAGTCTAATCTTTGGAAGAAAACAGCTGTGAATTTATTATGCAAGGGAGAAGCATTGCACCGTCTAGTAACTGTGAAAATATTGTATTATTGTCATCTTTCTTTCCGGAACGAATTTGTTTATTTAAAATAGTGATACAATACTAATATCCTAGCTAAAAGTATGACTATAAGGATAAAGTAAACAATTAGTCAAATTACAAATAGTACGTAATTCAGTACTTAGAAATACCTGCGGAATGTCTTTGAAAAGACTTTCATTACTTGCTTATATTATAATTGATTGTGACATTTGGGTACAGATTTATAATTAATAGCCTTAAGGATGAAGTACATACGACAAGGTTTGGTTGCGGACATATACCCAATCATAAGAACAATGAGATTTGTGGGACATTATATTTTGCGATATTACCAAGATGATGGTCCTATGAAAAGAGCATTTCGAGCTATTTATTCAGTGTCTAATATTTCATTAATAACGTTACATTTCTTACTCGGAGCGGTAAGCATTATGTTTAAAATGAACGACATAGAAGGTCTGGTGGCCAATGCTATATCAACCTTCTTTGCTTTCCACGCTGTAACGAAAATGATCTATTTCGCCGTGAGAAAGAAAGCATTTTATGAGACACTAGATTGTTGGGATGTCACCAATAGTCATCCAATGTTTGCCGAATCAAATGCTCGTTTTAAAATGAGTGCGATTCGAAGGACAAAAGATACTTCTGCTATCTGTATCTGGAGGATGTGCGCTTTTTCATCATCTTCTGTCTATACGGCCATTTTTGTTGCACCGTTTCGTACTATTATGGACGGCAATGAAACGATAGTGGTGGAAAATTCTCCATTGATAGTGGATGCATGGTATCCGTGGAGCCTCAAAGATTTTACTTTCTTTGCAGCATCGTACTTCTACCAGCTGTATTGGTTGATATTCTGCATTTTCCAAGTCAATTCCATCGACGTATTGTTTTGTTCGTTTCTGATCTACGCTTGTGAACAACTAAAGCATTTGAAAGAGATTATGACTCCATTGGTCGAACTCAGCGCTGGAAGAGATCCCGAAGCTTTAAGGAAAGCCGAATTGTGGCCAGAGATTACWGCTATTGACAAATCTGCTTCAAGATTGGATGGCACACCTCCTCCATATCAAACAGCAWCAAGGAACAGGATTTATCCAGAAACGTTAGGAGTTGATATGGAACGATCTTTAGTCCTGAGTGATTTTGCTCATTTGAAGGAGCCAATGGTCACCTATTCAACCGATGAAGCGAATATTGGAGAAAACGTTCTTACCAAGAAGCAACAACTTTATGTGAGGTCGGCGATTAAGTATTGGGTGGAGAGGCATAAACATGTCGTGAGATTTGTAGAAAGCGTCGGAGACACCTATGGTCTGGCATTGCTACTTCATATGCTTACGAGTACCATCACCCTTAGTCTTTTGGCATACGAGGCGACAAAGATATCAGCTTTCGATATTTACGCCATGAATGTTATTGGATACCTACTCTATACGTTACTTCAAGTTTTCCTGTTCTGCATCTTTGGAAACGATTTGATTGAAGAGAGTCTCTCAGTGATGAAAGCTGCTTATGAATGCCCATGGTATAATGGATCAGAGGAAGCTAAAACTTTTATACAAATTGTTTGCCAACAATGTCAACGAGCGTTATCCATCTCAGGAGCCAAGTTTTTCACAGTGTCTTTGGATCTCTTCGCATCGGTTCTGGGTGCAGTAGTAACTTATTTCATGGTGCTGATACAGCTGAAGTAAAGATGTAACTTGGAGAGAAAATAAAACACACGAAGAATATGAATATTCCATGCATTACCAACTACGGAAAGTAATCTGTCAAATAATATTACTTACGTCATAACAGAATTAATCGAAATATCAACTTGATTAGGCTCCGTAACTGTGTTTATTAACACTTCATGTACCAACGATGATYTACTGTAGTTAAAATAATGGGTTAACCATAATGCTCTCTAAGATAGTTGTTAAAGGTGTCATATTGNCCTTTTTAAACATTAATATTTGTGATTCAATTTTTAAGCAGGCATTTTATCTACTGGCATCTAATAATCTAANGGTGACATATCCATCTCNATTGATTATCAGATTTTAGAATTACTTTAATTCTATCCGATGATGNTTCAGGTATTCGAGAATTTCCTTAGTTGTTGTGNCAAGGAATGATCAACAMACGTYCAGCAAGGTTAANCARGACTNGCCTNGCTAGTGKTGSCAGATTTTTCARAGG

>TdomOrco3_putative coreceptor of insect ORs_partial mRNA

ACATGGGGACCATAACCTCAAATTGTTCGTACTCACGAATACAAATTGTTTCGGTCTTGCCCTGAAAAACTGTCAATTAGTTTACGAATTGCCCGTTAATGTAATTGGAATATTGAGTATTATCTAGACTCCAATCAACTGGATAGTGTGTTTAACATAGTATCTTGATAAGGCAATCAGAAACAAACATGCTTGGTTTTCAAAGTTTACAAGTGTGCCTTCAGTTCAATTTTCGCTTGCTAAAAGTTGCTGGCCATTGGATAACAGAACCAGCTCAAGATAATAATAACAGACAAAATGCAGCTCTGCATTGGTCGTACTGGTTATTACTTTTATATCGTGTTTTCATAACGCTTATCACTACAATTCATGTCATTTCAGTTGTAGCAGGGTGTCTGAAAAACGCGGATAAATTTAAGGATCAGCCAATGATTGGTGCTATGGGGTTGTTTGCATTTCAAGCTCTTGTGAAGTTAGTTTATATTATTCTCAAGAGGGCGAAGATCAAAAATGTTCTCAATACTTGGAACGATACATACACACATTCATCGTTCATTTGGTCTCGTATCAATGCCATTGAATCATCAACGAAATCATCCAAAACGGTTTCAACTTGTCTCCTGGCTTCTTATGTAGTTTTGGCAATTCAATGGTGTTTAGCACCAACGTCTGTTTCTAAAGATACTACAGAGGAGTATAATATTACTACGTTTATTAATGTTACATCTTCATCAAAACCACTTCCATTTTTGGCTTGGTTTCCATTAGATTTTGAGAGGTCACCAATCTACACTTTTATCTTTGCCTTTCAAGTTGTTGCAAGTTTGTATTTTGCACTTATTGTTGCTGCTTTTGATGGATTATTTTGTGCATTATTGTCACAAGCTGTGAATCAGATGGATCATTTAAGAGATTCGCTTGGATTCCTGATTGATGTTTGTATTGAAAATGAACCATTGTTGAAGGACAATATAAAATCCCCTAATAGGACAGCTATTGATAATTTGGCTTCAGAAATTGCTGGTGGGCTTTATAGAAGGTACATAACATCCTCATCTGGCAGTCGTATTAGACCAATTGAATTGCCTTATGATCAAAGGATGAATAAAAGCACAAATTTAATAGTCAAGAGTGGACGAAACAAAGGCTTCACTCATTTTGGTGGTGACGACTATTGGGAGAACATGAGAATGTCTATGAGTTACTGCATTCACCATCATCAGTATTTAATCAGATTCACAGACACATTGGAAGAACTGTTTTCTGCATCCATGCTGATTCAATTTCTCTACAGTACAGGTCTCCTCTGTGTTTTGGCTTTTGAAGCCACTTTGATCCGCGGGTTTGACATGAAGACGTTGACTCTTGTGGTATTCCTGTTGGTCTCCGTTATACAGTTGTTTGCTATTTGTTCATATGGAAACAAGATCCTGTCAGAAAGTACGAGAGTGACAGAAGAAGCGTATTCCAAGGCATGGCACAAAGGCTCCGAAGATGTACGTAATGTTCTACAGCTAATATTTCAACGGAGTCAGAGGGCACTAGTTCTCTCTGGTGCCAATATATTTACTGTGGATCTCGAAACTTTTGCTAACGTTTTGGCTGCTTCATTTACCTACTTTATGGTCCTTATTCAGCTGGGATAAGCTCTCTACAGTTGTCTGAAATGGAATTGTACACAATAATCCAGTGGATTTGTATATGTGTGATAGTTGTTAATTTAACAACCACGTCATTAACACAAGCTTAAATAAGTAATGAAGAGTTAGTCATAAAAGAGAAGGAGCCCTCATAAAGGACATTTTCTTCGTCTACACTAAAGTACTAATTAGTCAGACTATGCAC

>TdomGR1_putative gustatory receptor_partial mRNA

CTAATACGACTCACTATAGGGCAAGCAGTGGTATCAACGCAGAGTACATGGGGATTTGGRCAAAGAAGACAATTTGAGCATAGTCGTTCACTGTTTTGTGTGAAAGAAATTTTATAACTTCTTCTATTATATTACAAACTACGATATTAACATGTGGTTTAACGATACCACTATCAAATGTTTCTTTACCCAATCACGTTCGAGAAACTGYAGCGGTCGGAGTTGCAAGGAAGTTAAAGTGAAAGATATGGAAACAGTATCTGATGTGTTTTATCATGAGTTAAAGCCGATCTTTGTGGTTCTTCGAATAACCGGCTGTTTTCCAATTCTTCATACAGCAGCTGGTGTTTTTACGTTCGCTAAATGTTCATTGCTATCTTTGGTGACCTTTCTCCATTATATCGGTTACCTGGCATTATCTATATACTTCTCAAGAAGTGTCCTGGATCTTATTGGAGCTAAAGATAGGAATTTTGATGATATGGTTTACGACGCCATTAGGCTAACATACTTTATAATGCCTCATGTGCATTTGGTCACATTTTTATGTAAAAGTAAGAATATAGCAGATTACTTGAAACATTGGTATGAGGTAGAGACATTATTTTTCAAGACAACTGGGAAATGTTTAGTGCTTCACCAGAGGAGAAAGGCATGGCTGATGATACTTAGCACGCCTGTTCTCGTACTGTTGGCCGGTACTCAACARCATTTTGTGTTCGCAAAGCTGGACACCTGGCACAAATTAACTTTCTGTTATTCAATGTCTTTGAGTATTTTTGTTGACATATTGTGGACTTTAATATGTTGGTCGCTTATTGAAACATGTAAAACTTACACCGAACAGTTGAGGAGTGTGTTATCAGACAGTTCAGGATGTTTCTTTGTTCACACGTTACGTCGTTACAGGGGTCTGTGGCTTCAGCTGTGTCGCTTAATTGAGGAAACAGGAAGTGTTCTGTGTTTCCAAT

>TdomGR2_putative gustatory receptor_partial mRNA
CTAATACGACTCACTATAGGGCAAGCAGTGGTATCAACGCAGAGTACATGGGGATAGTARAACAAGTAGATGTTCATACACTGCTCAGAAATACGTTTTCTTGTCGAATTCCGACCAGAATTTATTGCAATAGGTTCAATGTGAAGGTATTGTTTTCGCGATTTAAAAATGAGTGARAATCCTGAAGTCAGCTGGAAGGACAGATTATTTGAAGACTGGGCTGTAAGAAATAATGTTTACATTTCTTCAAAAAAACCTCTCAGTTTTCTTCAGTATTTTGGTTTAATGCCTTACAGTATTGAAAATTCCGAAAGATTAGTGTTTTCTAAAGGATGGAATAAATGGTCTTGGATATTTCGAGGTTCACTTCTTACAATTTTTCCACTACTTTGCATATATTTTGTCAAAGTACAATACAACGAATCAGATATAATTTGGTGGAGTTTACGTTTCCAAATAATCGTTGGTTTTTGTTTTATGTATTTCATACCATTGTGGTATTTAGCCAAAGGAGTGCGGAAAACCCGGAAAATATGTTTTCACTATTTCGCTGATGAAATTGTTGAAACGTATAAAATGATGAGTGATCAAACAATTATGGATCTTCTGTCGCCAGCTAAAGAGGAGTGTAATATGCCATGGAAGGCAATCGTGTGTTTTATTCTGTGTWTAATCTTTCTTTACATTTTCAAGATTCTGATTTCGTTGTCCATGGTAACGTCAATTTTTGGATATTTATATGGCTTGTTAGCTTTCGTCTCATTTATAGCTTCCCAACCAGATTTCATTTATGTATTAGTTTTCGCTTATTTGTGTCGTTTCTTATACACAAGATTTGATGCATTGATGAAGAAAGTAGATGAAGCAGCGAGAACTGAAAACGTGAAACATCTTTTCGAAATTTATAGAAAGTTATGTGACATAGTCGACTACGTAAACGATACATTTGGACAGTATTTATTGCTCTCAACATATTATAATTTAATTCAGCTTGTTGCTAGTCTTTTCTACTTCATGTATACAGTGTTTACAAATGAAAAAACGACATATAAAACTCCTTACATTATAATTGCTGCAGCAACGATTTCTTTTACCATTGTCCAATTTATTAATATTGTGTCACACTGTTCTGAAGTTGTCCAAAAGGTTGAAGAAATTCGAAACAAAGTTTTACAGAAGGCTTTCGTCGAAGTAGCAGACGGATCATTTGCGGAGTTTTCCTTACTGATGGAATCGAGGAAAGTACAATTCACTACCAATGGTTGGTTTAGTCTGGACAATATATTCCTGTACACGGTAATTGGTGCAGTGATTGTGTGCTTGGCGGTATTCCTTTCATTTCAAATAGATTTGTCAAATAACTAAGTGATTTTTATGGACTTGGAACGTTAGAAAATTAACTACAATGATGGATACTCACTTAGGATACAAAAT

>TdomGR3_putative gustatory receptor_partial mRNA

ATTAGCAGCCTTGTTGAAAAGGTGTTGGTACTCACATGTTTCTTTGATATATTAAATAAAATCTCTTCTCGTTTGAGAACTTTTCGAGTAAATTGTGCAACTTGTGTTATCTGCAAATCTTAGGCACATCTAATCTTAAAAGTTGGAGGGGACAAAACAGAAACAAAATGAACGTTTACGAAACTGCGAAACGGCCTCTCCAAGTGCTCCGATGTTTTGGTTTACTACCTTACGTTGTAGAAAATTCCAAAGAATTAGTGCGCTCTACAGGATGGCTTGTATATTCTGTAGCATTTAGACTGTTCGTCTCGCTTTCTGTTGTGGTTATGCTTCTTTATAGCTGCAACTATTATATTTTAGATCCACATAAAGTGCACTTAGAATTAGACGCTGTAGGATGGAGTTTGATCTGTCAAATAGCCATAAGTGTTGCTTTTATATTTTTCATATCTATTTATGATTTCTGCAAAAATGTAACCAAAGTATTTGGACTGCTAACTGAAGTAGATGAAATCGTTGTAACTTTTCCGTCGGCATCTTTCCACAATGCAAACCCGTGGAACACATTAGCGATTCTTAATATCTGTTTGTGGTTAGTGATATTCAAAACTGTGCTTTCTATTGTGATGGTCGTGGTGATGAAATCCGGATTATTATTATTCTTTGTACTAGCTTTCGTGTCATTTGTTGCTTCCTTTCTAGATTTAATTTGTATCTTGGCTTTCTATTACGTATGTCTTCTGCTGTACCAAAGATTTCAAGTAATGAAAACTGAATTAGGTCTTGTAGTGAAAGAATTATCAACTGATGTTCAAGGAGAGGGTTTGTTTCATTCGGTTACGAAACTGAAAACTCTTGGCGAACTCTACAACAAGTTGTGTGAAATCATCGACCGGGTGAACGCCACGTTTGGGTTATATTTGTTGGTCTCGATCATTTATAATTTTATTCAGGTTGTTGGTTGTGTTTTCTATGTCGTATATGTTCTATTTCCAATACTACATATGGATAAGATGTGGATAAGATATGGAAGATATGGATTAATAATGGAAATGGATAGGATTTCTAACATTGCAATTACTGCAGCAACGACTCTACTGAGTTTCCTCATATTTTACTGTGTCGTGTTTCACTGCTCTAAAGTTATGCAAAAGGTTAAAGACATTCACAACGAACTTTTGTACTCGATTGCGTTAGAAGATTGTCAATCAAGAGAATTGGAACATCAGTTGTTGAAATTCTTTCATCTGCTCGAGTTCAGGAAAGTGAAATTTACGGC

>TdomGR4_putative gustatory receptor_partial mRNA

CTAATACGACTCACTATAGGGCAAGCAGTGGTATCAACGCAGAGTACATGGGGGAGGTGTTGACTGTTCTCCAGTAGGTACTGAACAATATGTGTGCAGTGCGAATGTCCATCGTCAGTATTGCGTTATTTATCGTATCTTGGACGATTTTGATTATTTTAACGTTCTTTGCTATTGTTCTTTTAATACGTGAAAATATTTATAAAGACAGTATATTAACGTCAACAGGGCTGATCGCATGGAATCTTGACTTATTAATTGTCTCATGTTCGGGTTTACTCTCGTACGGTTTTAGCATAAAGAATTTCTACAAAATCAAAGAAATGATTCAGTTGATGAAAGGATATGAAGCAGAACTGAAAATTCCATCAAGTAAATTCAATGTTTCACAACTTTGTGTTGGAACCTATGTTTTTGTTCTTGTGGTTGTAAATGTGTATGATTGTTTGTCATTTACAAATTTCTTTCGGGATTCTAGAAGACTTTTTCTGACTTTCTATTATTTCCTAAGATTACCGCCAATTCCCGTTGATTTGTGTGTCTATATCATTCTAACTAAACTTGCCAGATGCTTTTCAGCCATAAACTCTCGCATTTCAACAGAACATCCATGTTACGAGTCAACAACAATACGACCCTTACAGTTGGAGCAAGATTTCAGATTTGTGTCAAAGCTAAGTGCGCTATCATCCAACTCTCTGTCAGCAGCAGATGTTCGTCAAATTTTACGTAAACATGCTTTGTTCTGTCAAAATATTGAAGATTTCACATCATTGTTTGGCATTCCTATTTTTGCTGCGGTTTTATCTTGTGTTATTTTATTAATAGCTAAATTGTATTTCTTTTGGGTATATGTCTATCACAAATTATTTGATAATATCAACAGTGTTATTGCAGAAGGACTTTGGCCTTTAATTTACATATGTAGATTGTTTGTAATCACGTGGTCATGTGAGCAGGTTACTGAAAAGGCAAAACGCACTGGAGTACTCTTGCATAAGATTCGAAGTCCCACGATGGATGCTGAAACAAAAGAAGAGATTCAGTTCTTTTCGTCACAGATGCTGCATAGGAAAATAGAATTTTCAGCTTGTGGTTTCTTCACGTTGGATTTTACACTACTGTGCTCGATTGCTGGCGCTGTGACGACCTACCTTGTAATCCTGATACAATTTCAAACATCGGTTAAAGACGAATCAACATCAACGTACTCGACTGCTTCGCCAATGTGGAACGTGTCTACCACACCACTTTAGTTACGAAGAAAACCAGCTTTTTCAGGTGTGCAGAAGTAATGGCAAAAATACCGCAAGAAATTACTCAGGGATGTAATGATTAAAACAAATACTTGCACTTTTTTTTTGTAAACTTCAATATGTGAAAAGTGAATGGAAAAGCTCAAGAAAGTAC

>PsicOR1_putative olfactory receptor_partial mRNA

CGCAGTTCGATGTCCTATCCGCCACCTTGAGGGGCGTGCGGCGCGTGGCCGAAGATGAGCTTGGACTAAGAGCAGGCACGGCCTCTACTCAGGAACTATTCGACACGGAACTACTGCCTTGCGTCCAACGTCCTTCAGAAAGTGCTAATTACCATCTTATGCTTGATCGCCAAATGAAACAGATCCTTATTCACTGTATAAAACACCACAAGGATGTTATTGAGTTCGTAAGAAAACTCGATGCTGTATACAATCCAGTCATGCTGGGGTTAATCTTATATAGCATGACAATGATCGCCATAGCATCATTTCAAGCATCAGAGAGTTCTACAGAGCTTGGCGACTTGATGAAGTTCGCAGTGCTGTCATCAACTTGTATCTTCGAACTATATATCTTCTGCACGCTTTCCGAAGACATTTTACAGCGGAGCCAAGCTGTGGGCACTGCGGCCTTTTGCTGCGACTGGTGTGACGCAGGGACTGAGGTCAAAAATATCGTGAAGATGTTTATCGTGAGAGCCAACAGACCTG

>PsicOR2_putative olfactory receptor_partial mRNA

ATTTTCAGTCCAGTTATATTCTGTGAAGTTACGAATTGTCTTGCAATTATTTGCCAAGCAGGTTTTCTCGTGCTAGTGAAGAAAGGATCCATCGCCGAGTTTTTGAAATATTTTGGCTACCTTGTAGTCACTTTGTTCGAGTTGCTGTTGTACTGTTGGTATGGAAACGAGGTCATATATCAGAGCAGCATGGTGCAGTCAGCCGCCTACGACAGTGCATGGCTCAACAGCTCGCCGCAGTTTCACTCAACTGTTCAGATGGTCATGCTGCGGTCCCAGACGAAACTCGCACTCACTGCTGGCAACCTGCATACCATGTCTCTGAGCACGTTCACTGCGGTAAGCAGACTGCAGTTCACTGTGAAACCAGATCGCACCTCGCGCACAGTAACTTGCAGTGCTACCAACTGAGTCTTCTCCGTTACCTGTGTAAGCAGAAGATCCTGAAGAATTCGTACTCACTATTCGCACTATTAAGACAGATCAGCGAGAGTTGAAGCTATTCCACGAGCCATTATTGCAGAAATAGCACGTGAAGATACAGAAAAAATTTCTTCAAAGAAAATTTATGTAAATTAGTAATACAATATATATGACTACAGTGAATAAAACCAAACGTTGG

>PsicOR3_putative olfactory receptor_partial mRNA

GAGAATCAGAAATGATGAAAGGATTTTGTCAGCAGCACCAGGAAATGCTTAGATGTGCCCAGGAGCTAGTCGATCTTCTCAGAAACTGGCTTTTCTCTCACTACCTGGTGGCGTCGCTTTTGGTATGTTCGCTTTGCTACCGGCTCCAAGTGATGGAGGACATGCGCGACTTGTTCACGCAGGTCAGTCACCTGACTGTCGTCATGTTCAGGCTGTTCACGCTGTGCTACTTCTCTTCTGAACTCACTCACCAGAGCTTGCGCGTGGCGGACGCGGCGTACTCGTGCGCGTGGCACCACCTGGGCGCGGACGCGAAGAGGTGCCTGGTGATGATCGCGTGCCGGGCGCAGCGGCCGGTCGTCATGAAGACCGGCCACTTCGGCGACATGTCCCTCAGTACTTTCTCCGCGCTTCTTCAAGCAGCCTACTCCTACTTCAATATAATGAATGACTTCGGTTAACATCGTTGTACTGATGTGCTGGCTAGATAACAATTTAGTAATTGTTAGGCAAGCAAATATTCAAACGAGACGCCTAAATTTATTTTTGGAACAGCTTGCTATAGCCTTGTAACTTTCATTACTATAGTCCTTATGTCAT

>PsicOR4_putative olfactory receptor_partial mRNA

CAGAACTGGCAGCTCATCAGAAGACAGATCTTCTGACATATATGGCAAGCTGGAAGGGGATAACTCATCTGAGTTGGAGAAAAACCTTATTGACCTCGTGAAGCTGTATCAGGATATCCTGTGTAATGCGCAAGCTCTCGCTGATCTTCTGAGTCCTCTAATGTTTACTCATTACCTCACGTCATCCATCAATGTTTGCCTGGTTGCGTACGAGACAGCTATGACTGATGAAATATCTCAGATGGTGGCAGGAGTTAGTCATCTGATGATGCTGATGGGCCGCATATTTTTGCTCAGCTACTTCTCATCTGAGATCATGGACCAGAGCATGAACGTCGGCCAGATGGCATATTCTTGCAACTGGTTATCTTGCAGCAAGGATTTCAAGAGAATGCTGATGATGATGATGTGTCGAGCAGAGAAGCCTGTTGTCATGAGAACTGGTTGGTTTGGCATCCTATCTATGGAAACTTTTGCTAAGCTTATGCAAACAGCTTACACTTACTACACCTTGCTGAAGAAGTTAAATATATAATGAAC

>PsicOR5_putative olfactory receptor_partial mRNA

GAAATATTTCATCTACTTATACTTAATAGCTAAGCAATGGTGCTACTGTTTCCTGCTGATGGCCGGCACACTGTGCTACATGTTGCTGCCCCTCACGGGCACCTCCGGGCGGTCCCAGATGCCGCTGACCACCTACCACCTGCTCGACCGTGCCGACCCGGCGTCCTTCGCCGTCGAGTACTTGCTGCAGGTGTGCTCCATGTTCGCCGCAAATGCCATCATCGTGCCGTTCGACCTGTTCGAGTTCTTCGCCATGAAGTACTGCGCGCACCAGTTCCAGGCGATAGCTGTCCTCCTGAGTATTGCCGACGATCGGAAGGCACGCCCACGCGACCACGGTTCCAAGGTGGGCAGCGAGCCGGACATCGCCCCGGCACCTTCTCCTGGCGGGGACGGCTTGGACGAAACATGGCACTCACTTATCCGCTACCATCAGTCCATTCTCGAAAATGCTGAGAGACTGGCGAATCTCTTCTCTCCGTTGCTGTTTGCCGATTATGTTACCGTGTCAATCGCC

>PsicOR6_putative olfactory receptor_partial mRNA

CAAATGTTTTTTGCCGAGATAGTCTTGTAAGCAGATGGATGTTTTCAGCAACGCGTTATTTGTCCAGTTCCTCACTGTGTCTGTGTCGGTTTGCTTCTGCAGTTACCGTATCATCCAGTTGCCACCAAGTTTGAAGATGGTATCCATCATTGCCTATATGTTCTGCATCATTGGTCAGCTTGGCATCATCTGCTGGTATGCTGACGACCTTTCCGACAAGTGCCTCAGAGTTGGTGAAGTAGCATTTAGCAGTCGCTGGTACTTAATGCCAGCGTCTTTCAAGTTCTGCATTCTGCATACGATCACGAGGGCCCAGAAACCTGTGACCTTCAAAGCTGGCTATTTTGGTGTTATTTCAATGCAGTTGTTTGCTGGGCTGTTGCAGACATCATATTCATACTTCGCAGTACTGAAACAAGCACAGCTACAGTAACAGCAGCTAATGACAGATGGAATTGACACTGCAAGATGAACTATGTGCTGTATGTTCTTGCATAGCATACTTCAAAATCTAACTTCTTTTTAGAATTTATTTGTGTGTAGCAATTGTCACACTTTATGTTGAGCACAATACGTATTTTCCCTGAGCACAATTCTTGGCACTCTGTATGGTGACTGTATAAAATTAATAAATATTAGAAAATGCATTTCCTGATATGTAAAATTTCCCTA

>PsicOR7_putative olfactory receptor_partial mRNA

ACTATTTATTGTGAGGTAGAACAATATTCACAGAAATGTTTTTCCAGTGATGAAAGCAGCTCGTAATGACGAAATAAATTCAACCACAGAAGACAACGTAATATTGTTGTCAAGCTTCGTCTACCGATTTTGGAAGGAAATATTGTTGTCATGAAGAAGCATGCTGGAGGGATCATGACTCTCCCAATGACCTTGCTGGGCGTTTTTGGACTATGGCATGGCGATGGTCGTTGGGCCATGAGCGTGGGGCGATTCTTACGATGTCTCACACTTCTTCTGTCGGCGAGTTTCCAGCTTTCCTTGCTGCGACCTTACTTCCACTTCTTCGATGACCCGGTGATGGCGTTCAGGGCCTTGGGATGGCACTCGACAATCGTCCCCACTTACATCATGGCATTCTCGCTACTCGCCAACAAGGATAAGTACGTGAAAATAATGTCGAGGAAGACTGCCGACGAGGACAATGAGGAAACTGGGCAGATATTCTCTGCCGCGCTGGTCCAAGGTAGGAAGCTGACCATAGTATACTTGTGCCTAGGGACGGTTTATACGCTGTCCACATGGGCTGTTCCTCTGGTGATGTTCCACATTAAGCCTTCTCAGACTCGTGTGCCCTACGACATCTGGTTTCCCGTACCTGCCAGTGACCCCACAGGCCTCGTGGCGCAGTATGTCTTTCAGAGCACCGGCATTGAGCTCAGCTGTTATATGGTCATTGTATGTGACATGTTCTTCATCTCAATGATGCTTAAGATGATCGGTGACTTCAAATCCTTGAACTGTGCTCTCATGCAACTGAAGACGAAAGATGTTACAAGGCCAAGACTTATCTTTAAGTTATGGAAAAATTTCACCTTTCAGGGAAGTTCCATTGGCGACATAAGCTCTCTGCAGATGGCCCACAAGGAAATGGATACAAAGCTCGCAGCACTAATTCAGTACCATCAAGATCTACTCAGGGATGTAAATGAACTCCGGCAGCTAATGGATACATTCTTGTTTGTACTGTACTTGTTGGGATCTGGCAACTTGTGCGCCCTCGCTTACGAAGTTTCTAAGGTTTCAGGATTTAATCACCAGATGACATCTGCTGGCAGTTACCTGATGGGCATGGTGTTCCGGCTAGGCGTTTATTGCTACTATGGCTCTGAAATCACTAATGAGAGCCTGAAATTAGGGGAGTCACTCTACTTCAGCGACTGGTTGTCAGCATCCATGAACTTCAAACGATGCTGTGTGAATGTGATTGCGAGGTCACACCGTCCATTTCGGTTCACCGCTGGAAAGTTTGGAACACTTTCTCTGGATGCATATTCCAAGATAATCAACACAGCTTATTCCTACTTCATGCTGCTGCGAAAGATGCAGACTACTGGCATCTGAAGGGAAATCCAGTGATCATAGCAAACACACATTTTAGTCCCCTTAGTATTTGACATGAAATGATGCTACCAAAACTTATTTAGTATATCTTCACAGAAGGGCCCTACAAAAATATTACTTTAATGCACTTGCACTTTGTAACATTACATTAAAATTAAAATGTTTTGTTTGTTTTTTTCCTTCACAGTAGAACCTCACAAAAACGTTTTTTAATACACTTGGAAATT

>PsicOR8_putative olfactory receptor_partial mRNA

AGATGCGCGGGGCGGGGGCTGACGCTGGGGCTGGCACGCGCACGGCTAGCGTGCGCTGCAAGCAGGCGGTGCAAGAACTGCTGGCGTCTCTACTGCGCTCCGGGGCTGCGTCCTGGCATGTCTTCGCCGGAGGCGCGTTTCTACTACCTAGGAAGGATTTGGTACTACCTCGGGCTGGACATCTTCGATGATGGCCCCGGCAGGAAGGTCACAGGCTGGGTGTATGCCAATTACTTCTGCTTCATTTACTGTATTGTGGCATATTCTTCATATGTTTACTCTATCGTAATCGCTGACGAATTAGAAGAACACGTCCACGACATAGCCATTTTATTCACGATGTCCTACATGTTTTTTGTGTGGATTTCTGCTACTCTCCGCAGGAAAGATTATGCGGAGATAATTCTGTACCTGTCTCGAGATGTATGTAGACAGTATGTCCATGCAGATGTTGGAGATGAAATAGTTTCAAAGGCAGTTCGTACGGAACGAAGTATAGCGAAAGGCATCACGATTTGGTTTTGTACCTCCGCTATGTCCCAGACCTTGGCTCCGTTTTTCACAATGCACGTATACTATGAAGGGGGTACAGCTTTTGTGAATGCGTCTTCGCCAACAAATAACTGGACACCATTCGACAGAACCATACTCTGGCAGTTTTTAGCCGATTATTTTTTTGCTAGCGTTTTAGTTTTGATTGGTACTTATATTTCAATAGCTACCACAATATACTTGTCTATTATTATGCTTCATGCGGCAAGCAATTTTGAAATGCTTAATGCCGCGATCGAATCACTGAAGTTTGAAAATGTCGAAGAAGGAAGTATTGACCATACCGATTCCCAAAATAACGTCTGTGATTCATTACCGTCTTATTGTTCGGATTCAGTGATTCAACAAGAAGTACGCGGAGAAGCTTTTGACTTGAAAGTGTCAAGTGTGGGACGCAAAGAATCCGAATTAATGAGTCGCTTAGGACATCGTGAAAATACCAAGAACAACTCGAATTGCGTTGGCGAGAATGCAGAAACCCAGCTGTACGAACTAGTGGCATTGCACAAAACACTGAAGAGCGTGGTTGCCAAAATGAATACTTTGATGACTCCAGTACTGGTCCTATATTTTCTGGGATTAGCAATGAACTTTACTGTCCTCCTCTACGAGATCATCACGATGTTTGCCGGATATGATTCCAACTTCTGGATGTATGTATTTCACATTGGAGCGGACTTCTTTCGATTTGGCCTTTGCTGCTTCTTTGGGAACATGCTTACAGAAAAGTGTGAAGAAACTTGTAATTCGCTTTATGCGACTGAATGGTATACTCTAACACCGAAAGTCATGAAAGTCTGCGGGATAATGCTGGCGGTGTGTCAACAACGAGTAGTTATTGGCGCTGGAATACTGGGAGACTTGACAATGCCGACATTTGCTGCTGTTCTGAGTGCCTCTTACAAGTACTTAAATGTACTGGCAAATTTAAAAACTGAAAATGATTAAAACAGGCACACTTAAAAGAACAGGCACAGCAGGTTTCATCATGAGGGACTCGACGACAAAATAATCCTCAAAATTGTCTTCATTTCAGATAAGTAATGTATGATTTATGACACAAAATAATTGTACTTCACTGATACCAATGTACACTACACGAAAGATTTGCAAAAATAAGTGTTGAACATTTGCACAGCATCAAGATTATATGAATGTATGCATAAAAATCCAAAAGATCGGAAGAGCGTC

>PsicOR9_putative olfactory receptor_partial mRNA

ACTGTCATGGCGCGTCCTCTTACGACTCGTTTCAGTTATGTCGGGAACATGTGGTACTATATTGGGTTGGACATATTTGGAAATAATGACAGGAAGACCAGGATAAGGGCGTTTTTTAACTACTTTTTGTTTACTTACGCCGTGTTGGGCTATTCAACATTCTGTCACCCCCTTATTGAAATGGTAGCGCTGGAAGAGCAAATACATGATATCGCCATCCTGCTGACAATTTCCTATATGTTTTTTGTTTGGATCTCGGTTACTTTTTACAGGAAAGAGTACGCGGAAATAATTGTTTACCTCATGCAAGACACATGTAAATCTTACATCACCGACAGTGCTGCGAAGAAAATTATTAACAAGGCAATTGGTATGGAACGAATGGTTGCGAGAGGCATCCAAGGTTTATTTCTCACTTCTGCAATGTCACAGATTGTGTTTCCTTTCTTCACAATTCAAGTGTACGAAGAGGGGAACAGAACTGTTGTGAATAGTTCTCTACCAGTGCATAATTGGACACCATTTGACCGATCCATCCCTTGGCAGTTTGTAATTGACAATATGTTCTTGACGTTCTTAGTTCTGACAGCTACTTATGTGTCCATTGCAACCACAATTTACTTCATTATTATTATAATTCATGCAGCAAGTAATTTTGAATTGCTTAATGCCGCGATTTCCAGCTTGAAAGCGGAAGTACTCAGAGAAAGTGGGGGAGAGTGCTTTCAGAATGCAAACTACGAAGATTCTGATGGTGAACCAGAAACCTTGGAGAGTGTAGATGTTTCACCGTCATTAGAAGATTTTCAAAACACAACTGGCTTGAAGTTGTCTTATGCAGAAGACAAGGACTTTCAAATTTTATTCCATCCAAGAAGTCGCGATAGAATGTACAGAGACTTGTCTTATAGCCTAACTGCAGAGGCCAAACTGAACGAGTTGGTGGGAATACACCAAGCATTAATGAGTGTTGTTAACAAGATGAACGATATGATGACTCCTGTGCTGTTTCTGTATTTGTTGGGATTGGCATTAAATTTTACAGTTCTGCTCTATGAAATTGTGACTATGTTCGCAGGCTTTGAAGCTGGATTTTGGATGTGCGTCTTGCACATTGGAGCTGATCTGTTTCGGTTTGGCCTTTGCTGCTACTACGGGGACATTCTTACGACACAGATTGAAGATACATATCAGGCCCTTTACGACACTGACTGGTACAAATTCGCACCAAATTTCAAGAAAGCCGTTAATGTAATGATGGCTGTTTGTCAAAGACGAGTTGTCATAAGTGCTGGACTCTTGGGAGACTTGACATTGGAAACATTTGCTGCAGTTCTGAACACATCTTATACATATTTGAATATTCTGGCTAACCTACGAGAGTCAGAATAACTTCTCTAGAAGAATAGTCCAAGCACGGTGAACTTCGTCAATCAGCTGGATAAATTTAAGCTTGTACTCATACAAATTAAGATCACAAATAACATACTAGCATACTTTGCTTTGAAATATATCCTGCTACTATTGCATATGTAAAGTTATATGATATTTATGTAAAAATAAAATTCACTATTGCACTGCATTCCAAATGGCATAAGTTATTGAGAACAAATGTCCTCATCTAGATGC

>PsicOR10_putative olfactory receptor_partial mRNA

CAGAACTACCGTAAATCCAATAATCCAAATGGAAGAAAAGTTATCTAAACTCATCGAAGTGCATCAGGCAATACTCGGGACAGTTGATGATATGAACTTCGTTATGGGTCCAGTCGTGTTTCTTCTCTACGTCAGTGTGACAATAAACCTGTGTGTCCTCATTTTCCAAGCTGTTGTTGCACTGGAGGAAGACACTCGTGCCTTTGTAATGAATGCTTCTCAGCTTACTTTTGTAATGCTGCGTCTTGGAGTTTACTGCTACCTGGGGAACGCTTTGACATCACAGGCTGAAGAAACATACCAAATAGTTTATGGCACCAGTTGGTACAACTTTTCTGGGAGATTCAAATCTACTATATACATTATGATGGCAAGAGCTCA

>PsicOR11_putative olfactory receptor_partial mRNA

CTGTGCAGTTCGTCTTTGATCGGATATGTATGGCGACTGTCATAGCGTGTCTGATGTACCTGTCGTTGATAGCTAATTGTTTTTATAACAGCCTGCTCATCAATGGCGCCAGTCATTTTGAGTTGCTGAACGCATGTTTGTTAACTCTGGTGACTCAAGACGATGGTGACATCTTTTCCAGTTGTGAAACAGGAGAAGAGAGCAATGAAGAGCTGCCAGATCTCGCTGAAGGCACATATGGTGCAGTGGATGATCAGACTTACAATTTGCCCAACACATTGAAAGGCAGTTTCCATTATGGAAGGAAAGAAATCGTCAGCGGGGCGATTTCTCCTGGTGTCAATCGCGAGGTAGGCAGAACTACCGTAAATCCAACAATCCAAATGGAAGAAAAGTTATCTAAACTCATCGAAGTGCATCAGGCAATACTCAGGACGGTTGATGATATGAACTTTGTTATGGGTCCAGTCGTGTTTNNNNNNNNNNNNNNNNNNNNNNNNNNNNNNNNNNCTACTACCTTCCTAATGATCACTACTCAGCTTATTTTTGTACTGCTGCGTGTTGGCGTTTACTGCTACCTGGGAAACACTTTGACCACACAGGCTGAAGAAACATACCAAATAGTTTATGGCACCAGCTGGTACAACTTTTCTGGGAGATTCAAATCTACTATATACATTATGATGGCAAGAGCTCAGAGACGTGTGGCAATAAGCTCAACGATGGTAGGAGAACTATCCATGGAAGCATACGCAGATATCCTGAACAAAACCTACACATATTTCACAATCTTGAAAAATATGAGAGAGTAACTGTCAAATGTCAATGCAGTGAGGAAAATGCAATATTTACCTGGCCATGTATAAAATGTGATTTTTTTTATTGCCGCATTTAATGCACCTTTCTGACGTCAATGATATGCATAAATATTTCATATAATTTTATGCCTGGAAAAGTTATGTTTATCGGGTAGTTATATTTCGCGATGCAAAAGCACACTTTATGCTTCGCTGGGAAAATAAATTAATATCA

>PsicOrco_putative coreceptor of insect ORs_partial mRNA

TCCGATCTCATTGGTCCACGTGGGGTTTTGTGACTGCAGTACGTATTTCTAAAGGCATCGGGACGTCGATATGCATTCACAGTTCTTTGGTTTACGGTGCCATCGCCTCAAGTGTCATTGAGAAGAAGCGCATCGATCAGTTGTCTGCAAGAACAGTGCCGACAAGTGCTGATCATTTGTTATCCTACTGTCATATAACAGTGAATTTTCAAGATGCAGAAAATGAAAGTGACTGGCTTGGTGGCAGATTTGTGGACCCACATCCGTGTGCTCCAGATTTCTGGGCATTGGCTCCTCGACATTAACAAGAGTAGTAGCATGATGTGGAACTACTTGCGTACTGCGGGAACAATGATGCAGTCCCTTTTACTTGTCATGAACTATGTATTTATGATCATTAACTGCACACAACAGACGGCTGACTCGGATGAATTCACAAGCAACGTTGCTACAATTCTCTTCTTTAGCCACTGCGTATTTAAATTGTTCTACTGCGCAATGCGTCGACGCAAATTTTACAGGACTTTGAACAGCTGGAATAACACAAACAGTCATCCTCTGTTCGCTGAATCGAGTGCTCGTCACCATGCCAATGCTACTAGCAGCATGAAACGTCTGTTGATTATTATACTTTCATGCACTTTAGCCAGTGCATTTGCTTGGATAACCATTACATTCTTCGGGGACAGTGTTATCCATGTCAAGGATCCTGAAAATGACAACAACACTCTGATCGAGGAGGTGCCTCGACTGATGATTCGTGCGTGGTATCCATGGGACACCTCGAATGGTTTTATGTATGTGGTGACCTTTGTGTATCAGATGTTCTGGCTCGTCTCCATGCTTGTATTATGCAACTTACTTGACACTTTGTTCTGCTGTTGGTTAATATATGGATGCGAGGAATTAATTCACTTAAAGGAGATAATGAAACCATTGATGGAGATCAGCCATTCTATGGATGCCATAATGCCGCAGACTGCTGATCTCTTCCAGGCCGCTTCTTCTTCTTCCCATGCAGCACTGCTTAGTGCATCAACAGATGGATTGGATTCCAACATTCGTAGCATTTACAACAGCTCCTCTGAATACTCTGGTCTGCGTCACGGAATCGGCACACTTGCCACTGTGCAAGGTTCTTCAATGGGTCCGAATGGTTTATCAAAAAAACAAGAACTATTTATTCGATCAGCCATTAAGTACTGGGTAGAGCGCCATAAACAAGTGGTGCGGTACGTTAACGATATCAGTGACACTTATGGTGGTGCACTACTGGCGCACATGCTGATCAGTACTGTGGAACTGACACTGCTTGCATATCTTGCTACAACGATAACAGGACTTAACCCCAGGGGATTGTGTATAATCGGCTACGTTATCTATTCTTTTGGGCAAGTATTTCAGTTCTGCTACTATGGGAACCAGCTTATAGACGAGAGCTCTTCAGTGCTGGAAGCAGCATACAGTTGCCGTTGGTACGATGGCACAGAGGAGGCTAAAGCATTTGTACAGATCATTTGCCAGCAGTGTCAGAAATCAATGACCATTTCAGGAGCCAAGTTCTTTACAGTTTCACTTGATCTCTTTGCATCGATTCTTGGCGCTGTGGTCACCTATTTTCTGCTCCTGATTCAACTCAACTAATTGTTATTTTCACAAGAGCACGATAATTGTGCAAACATTATTCTTTATGATACTATGGATTGTTTCCAATGATCTAATCAGATGTCACAGAATGCAAGAAATATTCTAGCAAATTTTTTATGATCAATTCATACTTTATGAAAAATGCATTTAATAGGTACAGATAATTTATAATATTGCATTTGCACACAGGGGGTGAAAATGTATAAGATTAATTTGTTCATTCAAGTAAAATTGTAATAAAAATTCTCTAGCAAGAACAGCAAATAAGCAAATGTGCATAAATTAATAAATTCAAATATGTGTTGGTTGCACAAAACAAATGTTCACTTTCTAAATTATTTGTATGCACCGAAAACTGTATTTTTCACTGTCACGAAACAATGTATGAAAGCAAAATATCATAATTGTCAAGCAAATCTTATATTGTTTATTAGATGTAGATATCTGCACAAATCATCTTGATCATAGAAATGACTATTTCTAGGGGATACATAGTAAAGTAAAATTGTGAAGTATCAAAGTGCTACACTGGAGTATTGGGGAAAGGATTTAAATAAAAATATTCTCTATACTAGGTTATTTAAAAGAAAATTTCATAAATAGCAGATGGATATTTTGGTGTATTTGGTTAGCGTAACTGACTGATAACTAAATGACAACCTGTGAATGTATGGGTTTCTATTGTTATTAATATTTTAAGAACATATAATTTTGAACACAAATATTTAATATTACATTAACATTTATTAAGCGAAGCTAATTTTTACACATACAGCAATAATATATATTTTTTAAGTAATTTATTTGTCATTTATGAGGAAACAAATAGTCTGCAG

>PsicGR1_putative gustatory receptor_partial mRNA

TTTTTTAAAGTTAAAGCATTTCTTGTGAGAGAGTAAAGTGGTCATTGGAGAAGGGCTTGTACCTCTACATCAAACAGTGCTTACCTTAACCCATGTCCCTTTACCACTAGTAGTTTAGGAAGTACTCCTCTAAAGTACTATGAACGCACACCAAGTTATTGCTCCCAGCGGTGGTGTTCTATGCGCTGTACCTGCTGGTGTCGACACTGTTCCTGGATGTGGCGACCAAGTGGCCGCTGCTGGCACAACAGTGGCAGGTAGTGGAACAAGCGACAGCACACCAGGGCACACCCGTGGCCTTGGGCGCCAAAGTGAAGGCCTGCACCATAATCATTCTGGTTGCCTGCACCGTGGAGCACATACTGGCGCACACGAGGATGGCAATCATGGCCACCNNGGCAGCCTGTCAGAGTGGTTGGCATACTACTTCCATGCAGAGTACCCTCAGGTATTCGATCACTTTGCTTACTCGGCATGGGCTGGCATCCTGGTAGCTGCCATAGACCTGTTCGCCATGTACGTATGGAACTACAGCGACATGTTCATCATTG

>PsicGR2_putative gustatory receptor_partial mRNA

CACTCACTCGCGACGTCGACATGAAGCTGGCGAGCCGCGTGGATGACCTGCGCTGGGCCATCAAGCCAGCCTTCCTGACGGCGAAGCTGTTGGGAACCGCCCCTCTGACGGCCACCGACGACGAAGACAACGGCGGCGGCTACCGCGTCTCTTGGCCATGGTTGGTCTTCTCGCTGCTACTACACTCGGCCGCCGCAGGGGCGTCCGTCTCCTGCGCCGTCGACGGGTACCTGTCGACGCCCGAGGGCAGCGTCGCGAGGCGCTTCTTCGTGATTGGCGCGCTCCTGCGGGCCTGTAGGGACACCACGCTGGTCGCCGCGTCCTTGGGCTACCGCTGGCGCCGCTGCGAGGACGTCCTGTCCGAGGTCGCCGACGTGGACCAGGAGTTCCCGCAGTGCGCGCGACCATCCCGGGGGCGGCGGGCGTTCGCCTCGCAGGTCGCGCAGGTGTCCGTCATGCACCTGGCAACCGCGGCGTTCTGCGCCGCCTACGGCGGGCTGTCAGCGCACCCGTCACCCCTGGAGGCGTTCTCCGTGGCTGTGTTTTGCGAGGTGCAGTGGGCGCACGTCCTGGAATTCTGCGGCGTGCTCCAGGTGCTGGAGAGCAGGTTCACCGCTCTCAACGAGCAGGCGGCGTCGCTGGAGCGCTGCCTCCTGGCGCACGACGGCAAGGTGGCGGTCGCACCTCAGCTCTGCCGACTCATGCACTTCCACAGCCGCCTGCTCCGCCTTGCGTCGAGCGTCAACCGCAAGTACCAGCTGCAGGTTTTCGTGTACGCCGTCCTGTACGTTCTCTTCCTGACCTTGCACTGCTCCACCGTGATGGGGGCGATGCTGTGCCAGGACCGCGCGTCGGGTGACGACCAGGGAAGGGCCGTCATCTGCGCTCTCTACATGTCCTTCACGTTCATCTGGCAGATGGTGATGACGGCCAACCTCGGGCGCTCTCTCTCAGTCAAGGGGGATGAGCTGCTGATGACCGTCGCCAGGATGGAGCTCGTCAAGGGGCTGGACCAAGAGTGCACGGAGAAGGTGCGTCTCTTCTGCAGGCACCTTAGCGTGAGGAAGGTCGTCCTCAGCGCCTACGGCTTCTTCGCGGTGGACAACAGGGCCTTGGCGTCTGCCGTGAAGACGGGCGTCACCTACCTGGTGCTGCTGGTGCATCTGCAGAAGTACTAGTCGCCCTCTGGAGATGATACGCCTGCCGAAGCAATCTTTCTGGAGGAGAAAATATTAGCACAATGAACGTAGGAATTTAGAACTTTCATTCGGCTGCTGTAAAAATGAAAAAAAAAAAAAATCCTTGTTTTTCATTGATTTCTGCAAGAACGCTAATTTTTCAATTCTTTTGGTTTAACGCTACCTTATTCATTTGCATTGTTAAACGAAGAGCATTCAAATTTTTTATTCTATTTATTTGACTTAAGTGTATCAGTTGTAATTTTTTTACAAGATTAGTATTCTTGAATGTCAATTATTTAATTTGTGGTCCAACGTAGTGGTTTCCTACCGTACTGGTTGGTACTTTAAATTTCATGGTATCCAAAGCCTCCATTAATTTATTCTTGTCCATCTGATTTTGTTTTGCTGCGGTTATGTACCTTTGTGCCTCCCGATTACTTAAAGCCGTTTGAACGTTTACAACAGAGGTTTCATTTCTAGCTGCGGAGAAATTTATTTAATTACTTCTCAAACTACTTCAGTCCTGAAGTTATCCATTCTAAGCTTAACTTCATCTGCTAAGTGATTGATTTGTTGCTCCATTTTATTATTAATTCCTTTACTTGAAGCTCTCTATGATTCATGCAAATTTTGTGTCAGATTGCTCTAATTTTGTACTGTTGTTTGGTTCTGGAACCAATTGTGGATTTCCAGAAGAAACTACAGCATTTTGTGTGTCTTTAATTAGTTTATTTATGAGCTCGTGTGATGATTGTGCGATTTGTGGAATGTTTAACATCGTCATTGTTTAGTTTGAACACACCCCAGGTTATTGTTTGTCCGATGTGGCACGAGTGATTGTCTCATTTTCACCCACACGATTGATCGGGTCGCACCTAC

>PsicGR3_putative gustatory receptor_partial mRNA

CTCTTCGTCAATAGTTGTGACGTGCAGCAGCTACGTGGCGTTCGTCTTCTCTGTGTATCACAGCCAGTCGAGCCCGGTTCTGTATGCGGTTCTTTGTCTGTGGGGAGGCGTCCACTTGATGCGTCTTTGGATCACCACACACGCTTGCAAAGCAACTGCTCGCGAGGCGAACCGTGCGGCTGTTGTCATAACAAGACTCCTGACTGGGACAAATGACTCCACACACACCAACCAGCTGCAGCGGTACTCTCTACAGCTTCTTCGCCGCAAGGTTCAGTTCACAGCCTCCGGGCTGTTTCCTCTGGACCATACGTCCTTGCATAACATTGTGGGAACCATTACTACTTACATACTAATTCTG

**Additional contigs**

> Tdom_38756_putative gustatory receptor_ partial mRNA

CTAGTCGCCATCTTTGCTTCTAGTGTTTCCTAACGGCACATAGAAGGAACAGGCTAACAACACAAGTAGCATTGTGGAGAAGTTACTTCACGGGAACACAAAAGCGGAAAGCTCGAGCGAGCTTCGTCATTTTTCAAGACAACTGCTTCACAGGAAAAAAGTGGAAGTCACTGCCTGTGGCATTTTCACTATCGATCTAAAGCTTCTTCATTCGATAGCTTCTGCTGTGACGTTGTACTTGATAATTTTGATTCAATTCGACATTCAAGCCAAACATCATGTGATCATTGGTGAGACAGCTAATGATGAAAAGACGGCTTCACCTTCTACAAGCTCCACTACAATGACAATTCAACCCGACATTTCATTCTTTAATACCTCCTCATGAGAAAAATAGCATTTCTTGTTTGAAAAAAAAAAAAGTTCTACGTACCTGAAATTGTGATATAAGACATATGTTCAAGAGGGAAATATAAGATTGCATGTCTCA

>Tdom_61232_putative gustatory receptor_ partial mRNA

TGTAAGTTGTTACTGCTGCCACCACCGAGTGAAGTAGAGAGAGATTTATAGAAAACAATCCACAGGCTGTGAACTCTGCCTTCTTGTGGAGCAGTTGCGATGCGAAAAGTTGAAGCTCTTCTTTTGTTTCGGGATGCATGTCCCTTCTCAGTAGTCGATGCACAAGTACTCCTGTTCTGTCAGCCTCATTACTTGTCGATTCACATATTCT

>Tdom_87570_putative gustatory receptor_ partial mRNA

AGTAAGCAAAGTGAAATTCACAGGGAAGAATCCGTAGGCAGTAAACATCACTTTCCGATAACTTACTTGGCTTATAAATAGTTGTAGTTTCTCTCTTGCAGGAGTAGAGATCTTCCCATCTTGAAGTTTATGAACTAAAATACCAATCTCGTTGGACTTGTTCATAGTCGATACAGATGGGATAGTTAAAATAAACAAAGTCAGAATATCCATTATAA

>Psic_22013_predicted olfactory receptor_partial_mRNA

GCATTGGAAACAACAATGTTAGTACATTCATTAATTTTTATTAATGTTCAAAATTTTAAAAGTACTTTAAAATCATTTCATTTGGGCTAAATGGCTTGTACTCGTAGAGTGAGTAATAACAGCCACCTTTTTAAAATAGAGCATACAAAATTTGTTTCACCACAGCTGGAACTAACATCCTCTAAAATGTCTTCAAATGCATTTTTCATAACTTTCAAATACCCATTTCTTATACACATAAATTCAATCCTACTTCTAATTTGTGCATAAATTAGCGAAAAACTAATTATTTGAAGATGGTGGTTTTTCTAGTGGTGCAGTTGCTTCAGCATGGCGAAGAAAGAATATGATGAATTCATGAGCGCTGCATAAGCTTCCAAGGACATGGTCTTGAAGCCTCCTGCAGACAGTCTGACAGGTCTGTTGGCTCTCAC

>Psic_25058_predicted olfactory receptor_partial_mRNA

GTAGGTTTTGTTCAGCATATCTGCGTATGCTTCCATGGATAGTTCTCCTACCATCGGTGAGCTTATTGTCACATGTCTCTGAGCTCTTGCCATCATAATGTTTATAGTAGATTTGAATCTCCCAGAAAAGTTGTACCAACTGGTGCCATAAACTATGTGGTATGTTTCTTCAGCCTGTGTTGTCAAAGCGTTCCCCAGGTAGCAGTAAACTCCAAGACGCAGCAGTACAAAAGTAAGCTGAGAAGCGATCATTACAAAGGCAGTAGTGTCATCTTCCAGTGCAATAACAGCTTGGAAAATGAGGACACACAGGTTTATTGTCACACTG

>Psic_2682_predicted olfactory receptor_partial_mRNA

CTCCAATCATGCCGACGGCGTTACTTTTACTTTATTTTATAACCTCCACAACATGGTTTTGCCAGCCAATTCTCCTTGCTACAATTAAAGCTGTGGGACTGTTATGCATCTAAAAACAACAGTCACTGTCATATCTATTACTAGGCATGCTGACTACGTAGTAAAAACATTCCTTTCCTTGTCGATGTTTCCATGGCTTCTTTAAGAAGAGCTGAATTTTTTTAGCAGCGTGTAATATGAGTACGCAGTTCGGAGCAGCCCGGAGAACGTTTCCAGAGAGAGGACCCCGAACTTGCTCGCTGTGATGACCG

>Psic_31622_predicted olfactory receptor_partial_mRNA

TTGCAGAATAAATACAGTTCAACGAATAGAGCAATTGCAAGGGCAAGAAGCTTGATCAGACTTCCAAAGTCTGCAGAACTCTCTGTAGCTTGCAACCCAGTTACAGCTATATCAAACACGCTGTACAAAATTTGCACCAACATTACGGGGTTGAACACATCATCCAGCTTCTTCACAAACTTGATAATTTCAATATGGTGTTGAACACACGTTCTGAGATACTGGAACATGTGGCTGTTCAGCACTTCTTGTCGGCAATCAGCGCTTCCACCAGGAAGGGATACACCAGGTT

>Psic_31622_predicted olfactory receptor_partial_mRNA

TTGCAGAATAAATACAGTTCAACGAATAGAGCAATTGCAAGGGCAAGAAGCTTGATCAGACTTCCAAAGTCTGCAGAACTCTCTGTAGCTTGCAACCCAGTTACAGCTATATCAAACACGCTGTACAAAATTTGCACCAACATTACGGGGTTGAACACATCATCCAGCTTCTTCACAAACTTGATAATTTCAATATGGTGTTGAACACACGTTCTGAGATACTGGAACATGTGGCTGTTCAGCACTTCTTGTCGGCAATCAGCGCTTCCACCAGGAAGGGATACACCAGGTT

>Psic_31623_predicted olfactory receptor_partial_mRNA

CAAGACATCTCGCCACGTTCGCGCAGTGACTCTGGCACTCAGCTTTCTCATATTCCTCATATTCCTGTCGTGGACTGCGTCCCCGTTCATCACAGCGGGATTTTCGGTGACTGATCCCACAGGACTGGGCCCCCACCCGTTCATGTCGGCCTTCTTCTTTGACTACCAGTCGTCGCCGACCTACGAAGTGATCTACGTCTTTCAGACAGTCAGCCTTTTCTCGTACTGCCTGAGTGTGAGCAGCTTCGACACTGTGTGTGTGGCTTTCTTCATGCAAGTGGGGGCTCAGTTCAGAATCCTCCTTGAGTCGTTAAAAAGGCTGCAGGAAGTTGCGAAGGAGTCGCTAGCTA

>Psic_31984_predicted olfactory receptor_partial_mRNA

AGACTGGCGCATTTCTTTGCTGAGCTGAAGTGCATCGCACGCAGCACCGGTGCTAGTCACACCCCCGTCCTGTCGAGTCGTGGTGGCTGACGTCCGGCGGTCCGATGTGCCGTCTCCCTGTTTCAACCCCACAGCCACCTCTCGCAGGTTCCTCAAGGTCATGGAGAGAACCCTGAACTGCGCGCTGGCGTGCAGGACGAGAGCCATGCAGAAGGTGTCGAACGTGAGGACGCTGAAGATCAGCAGCAGAAGGGACAGCACCTGGAAGGCG

>Psic_44668_predicted olfactory receptor_partial_mRNA

GCGACAACACAATGAGGGGTGTTCTGAAGCGATGTTAAGGGTTAAAAGAAGATATACTTAAAGACTTCACATCACACTGAAACATACTCGACACAAAAAGTAAGTGTTGTTTTGTGCATGTTCTAAAAATATTGTAACTGGCTTTAAGGTGAGCATTGGCCATTATTACTACTTCATAAGCACATTTTGCGAAACAAAACTGTTTGTGGAAAACTTACAAAGCAGTGGTTTGCAACTAAAATGGAAGTATTTTTTTTTTCGCAAAAAAAATATTTTATGGTGAAGATGAAAAACACTAAAGCCGTCTACGGGGAGCATGACATCGCCAGTAAGATGTTGAACGCGAGAGTTCCGATGTTCAGGCGCCAGTGTCGTCCCCCTCGGTGTGCAGCTTCCACAACACCATGAAGTAGGACACTGCAGTCTTCGCCACCGACTCGAAGGTTTCCGAGGACAGAGGCACCACACCCATGGCGGTGAGGCGCAGCGGTCTGCCGCAGTGCTGGAGTACGATCCTCAAGCTAGTGCACACGTCCCC

>Psic_52266_predicted olfactory receptor_partial_mRNA

CCCGCCGCACAGGTACCACGGCTCGGCGTGGGCTTCCCTGGACACCAGCACGCTCGCCTCCTGCAGACGGTCGCCGCAGTGGCACAGCAGGTACAGCATCACCAGGTTCCCCGAGGCGTACCCCACCAGCAGCACGTACGGCAACACGCTCAGCGACACCACTATGCCGACAAATGCTGTGAAGCATAGTATGAGCAACGAGCAGAGGAACTGAACTACCCACATCGGAGAGTACAGCTTCTGAAGCTCCACGCCGAATTCGGT

>Psic_69815_predicted olfactory receptor_partial_mRNA

AAGTAGTTTTCTTGCAATTTTATTGTTGCATTTGCCGCATGATGGTAAAGTAGGTGTATGCTCCTTTGACAACCTTGAGGAAAGACTCCAGAGTGACAGTGTAGAATGGTCCAGCCTTAAGCACAATAGGCACTTTAGAGTTGATAAGTATCATAAGGGTGGTCTTTTTAAATATACTAGAACATTCGTACCATTGAATTTCAT

>Psic71173_predicted olfactory receptor_partial_mRNA

CCGAATTGCCTTTTCGTGGTAGATTATACAGTCGACCCAAATTTTTTTCATATTGGCATCCATTCTGTTTAGCGTTTCTGTTTTAATAAATTCCTGGGTGATCCGCCGACCCGAACACAGATTCCACTCTGTGTCCGGCGAAAATATTTTCTTGTTGCTTCTCCTAACGCTTTCCTTCATAACTGAATCGTCCAGGAAATCATCTGCGATCTGTACTTTCGACATATCTTGAGCGATTTCGCCAGACATTCTGAATAGCTCCGAAACCATTTCGAACCTGGAGCAGGCTCCGATCACCAGGAATATGTACACCAGCGTCAAGGCGGAGAACCAGGAGTTGACGAGCCAGCAGAAGAACACCTGAAACGCGTACGTGGACCAGTAGACGGCGGGGGACGAGCGGAAGTCGTAGGGGTACCAAGTGAGCAGCGGCAGGACCCGGGGAGCGTCCGGCTCCAAGGAGCCCATGACGACCGGGCCGAGGACCCAGACGAGTG

>Psic_7597_predicted olfactory receptor_partial_mRNA

GAGTAGGCAGCATTCACCATCTTGGTATACAGCTCCAAGGACACGACTGCAAATGATCCAGCTCTCATTTGGACCGGTCGCTGGGCTCTCATGATCATCACTGCCACTATGACCTTGGCTCTCGAATTGAACTCGTACCAACGGCAGCTGTATGCTGCATCACCGAAGGCCTGGCTCTGCGCAGATAACTGCGAACAGAAGTACCACTTTAAGAATACTACGGATGCAATCACATTTGCGAAGAGAATGTTTCTTAATTTCATGGCTGCGTTGTCATCCATTACAGCATTGTACACAGTCAGGCAAAGCGC

>Psic_78369_predicted olfactory receptor_partial_mRNA

CTCGACTGTCGCCGGCTCCGAGAGCTCACACCCGGGGGTTGCCTCAGCTCGGTGGCGTTGTACGGCAGCAAGTACTGCAGTTGGTAGAAGAGGTTGGTCGATAGCACACCCACCGCATAGTTCTTCCAGTCCAACGTGAACCCGATATTCAAGGCGGCGAACGTCTCCAGGGACATGACAGCAAAATGGCCAGCCCACAGCTGAGCAGGTCTCTG

>Psic_82237_predicted olfactory receptor_partial_mRNA

TGGTATAGGAGCATGCATATTATCAGCGAGTTGGTCACATAGTGTACGAAGAGCAATGTTCTAAGAAGTTCGCACAAATTATTTGCATTCCTGAGTGCTTCTTGATGATAGGCAGCTAATAGTCTCCATAATTGCGCGGTTTCATCTTTTCCACGAACCTCTAGTTCTATGTCGAATTCTGTTGTCACTCCGTCACATGTTTCAGTCTCTGAAGAAATTGCT

>Psic_61824_predicted olfactory receptor_partial_mRNA

TATCGCACAGACCTCAGAGATGCTAGAAGAACCCGGAACTGAGCGCTGATGTGCAGCAGCAGTGCCACGGCAGTGGTGTCGAACCCGCACACGGATGGAACGAACAGCAGCAAGCTGGCTGACTGAAGAAGGTACACTAGCTCGTAGATGGGTGACCTGCGGTGGTCGAAAAAGTATGCTGCTATCAGCGGGTGAGGTCTCTCCTGCTGCAGGTTGGAGCTATCAGCAAATACTGGGC

>Psic_31984_predicted olfactory receptor_partial_mRNA

AGACTGGCGCATTTCTTTGCTGAGCTGAAGTGCATCGCACGCAGCACCGGTGCTAGTCACACCCCCGTCCTGTCGAGTCGTGGTGGCTGACGTCCGGCGGTCCGATGTGCCGTCTCCCTGTTTCAACCCCACAGCCACCTCTCGCAGGTTCCTCAAGGTCATGGAGAGAACCCTGAACTGCGCGCTGGCGTGCAGGACGAGAGCCATGCAGAAGGTGTCGAACGTGAGGACGCTGAAGATCAGCAGCAGAAGGGACAGCACCTGGAAGGCG

>Psic_39262_predicted olfactory receptor_partial_mRNA

CTCCTCTTGGCTTCCGGGCTGTAGTTGTACCAACTGATGGCACCCAGCTCGTCACTCAGCGACACACTCTTGCTTGTAAGTCTTGTCGGGAAAAAATTCATAATAAATATTCGAACTGTCGTAGATCCCAAGAAGCAAGCGATATGGATCTCTTGAAATAATTCTTCTGTCCTATTAAACTGATACAACAGCATGCAGAGCTTCAGTGATGGTACTGCATATTGGAAGAAGAATATTGTACTCAGGAATTCACCCAAAACATTTGCAACCCTTAATGCTTCTTGATGGTAGGCAGCTAACTTCCTCAAGACAACTGCGATTTTGTCTTCCTTTCTAGAAACTTGCATCCTGACAGACCTTTCGTTATTGCCATATACGGTTTTTGAGAAATGCTCACTTTCTTCTTCCTCAAGTTTGTTTGTGCGATTTCTTCCTGTTAACG

>Psic_33458_predicted olfactory receptor_partial_mRNA

GTGCGTGGCCGAGTCGCTCCACATGTGGCTGGCGAGGCGGCGCTTCCACCGGCTGGTCGCGCTCTGGGAGGCCGCCGGAGAGTTCGACGTCGCGCACGGTCGCGAGCTGTCGAAGGTGCGACTGGTGAGCGTGTGCATCGCCGTGACCACGGTCACGCCAGCCATCGTGTACTGCCTGATGCCGGTGATACCAGGGATCCGCGAGGTGCTGGGGCGACGCCACTCCGTGCCCGCGTGGTTCTTCTTCGACACCGCCTCCAGCCCCGGGTTCGAGGCGTCCGTGGTGTTCCAGTCGGCAGCCGCCGCTCTCACCGTCGCCAGGATCGCAGCCTTCGACTGCTTCTTCCTGTCGCTGGCGGGGTTGCACCTCGCGCGTCTCGGTGGTCTGCGCCGCGACCTGAAGGCCGTCTTCGCCGACCTCGGGCCCGCGGCCGACGTGACGGCGTGCCTCCAGTCCTGGATAGTGCGGCACCAGCGGGCCACCGAATTCGGCGTGGAGCA

>Psic_10375_predicted gustatory receptor_partial_mRNA

GTCCTATTAATGCTAATATGTTACATTCTGGCTGGGTGTGCAACAACTGGGCTGTTGGTCTTGTGATTGTCGAAACTGAATTAGAATAGCCACGTACGTAGCTACTACACCAACCACCGATGTTACAAAGCCACTGTTTATCCCAAAGAAGCCACAGGCAGAAAATTGAACCTTCCGATGCAAAAGTTGAAGTGAAAATAACTTCAGCTCATTCTTGATAATCCTATTTTCAGTTTGATTTATGAGTTTATGAACAATAACTGCTGTGTTTGCAGCCTCAATCGGAGTGGAAAAAATGCTTCAAGCGCATGCAAACGTGAA

>Psic_33301_predicted gustatory receptor_partial_mRNA

CCTCCATGGAGAAAGACCTAGATGGGACTGACAAGAGTACCAACTTTGGCTCCTTGCTCTCGTCATTGACAGCGGCAGCACTGATGGCCACCAGCATGCTTCGTAGCAACAAGAAACCGAATGAGTAATAGAAGTACACTATGTCTTCAACTTTCTTCATAGGCCGGATGGTGTTATACAGCTGCAAGCAGATGAAGTAGACGTTGCTGGTGAAGGAAAGTAGAACCAGTTTTGATATGGTCTTGTCAATGCTTAGGCACAGCCATGACAAACTGTTATAGTCCTCGCGCATCTGTTGCCAGAACTCCTCAGGCTTTACCT

>Psic_40564_predicted gustatory receptor_partial_mRNA

AGGTGTTGTGAAGCAACCAGAATGGGCACTCCCCTTGAAGAAACTCCGGACCACGAGGCCGGCGGCGGCAATGCAGGCCCACGCACGGCAGGATGCTGCCGGCAGCTTCCACAGGGCAGCAAGGCCCATGCTGCTGATGGGGCGGGCCTTGTCTGTACTGCCACTACAAGGACTGCTGGCTCCGAATGCCTCCGCCCTCAGGTTCAGTTGGTGCAGTTTGGGCATGGTTTACACGTTGGCCGTACTGACAAGCTGTTGTTTCATGTTGTGGATGTCGGTCAGGAAGATTGTTGTGCACAGCTGGAACCTTGAGGATTCTTTTATTGCTCCC
