# Supplementary figures and images for: Evolution of insect olfactory receptors
Source: eLife. 2014 Mar 26;3:e02115. doi: 10.7554/eLife.02115 (PMC3966513; doi:10.7554/eLife.02115)

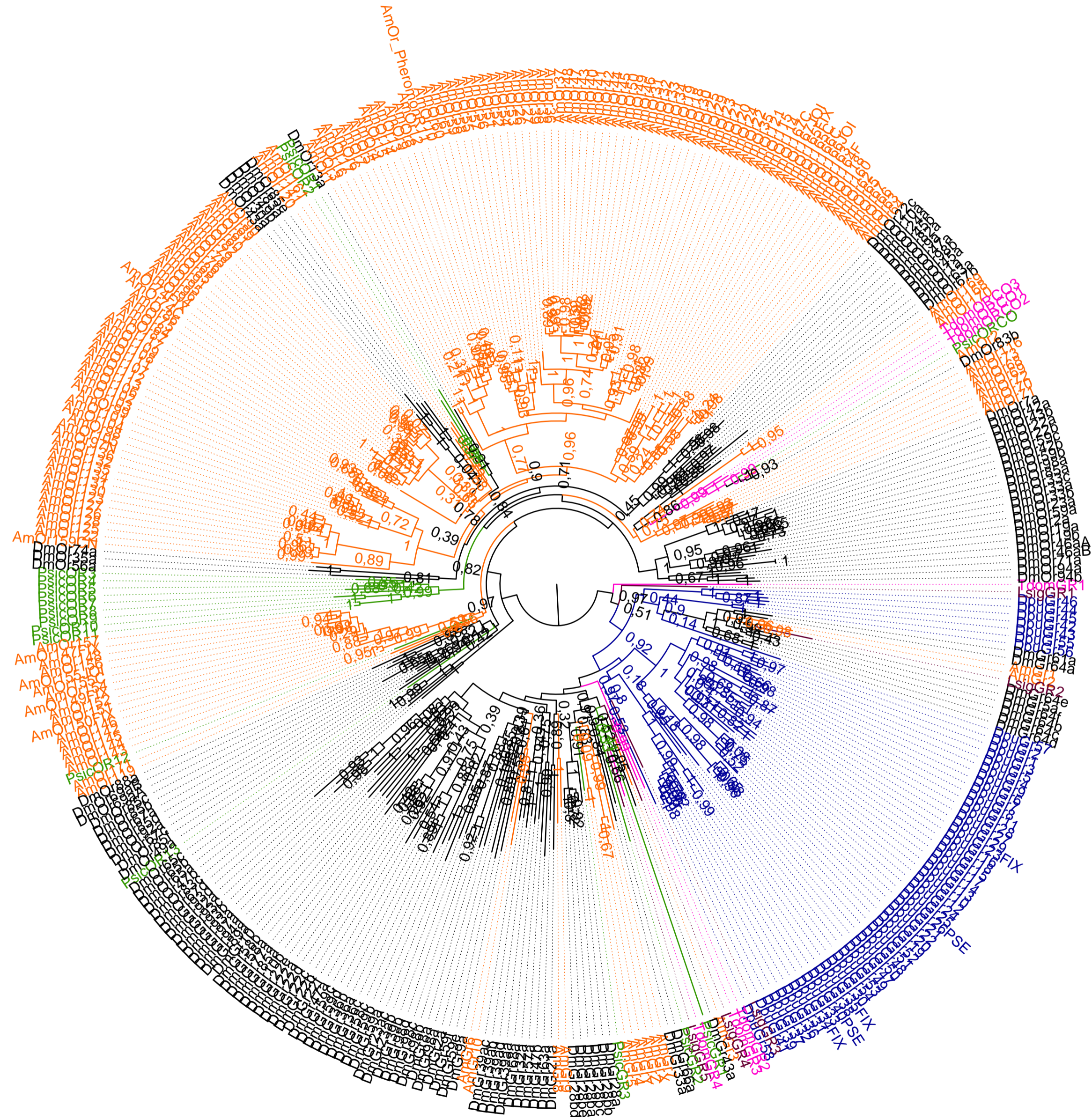

Supplement: Figure 4—source data 5. — DOI: http://dx.doi.org/10.7554/eLife.02115.014 [file elife02115s006.pdf]

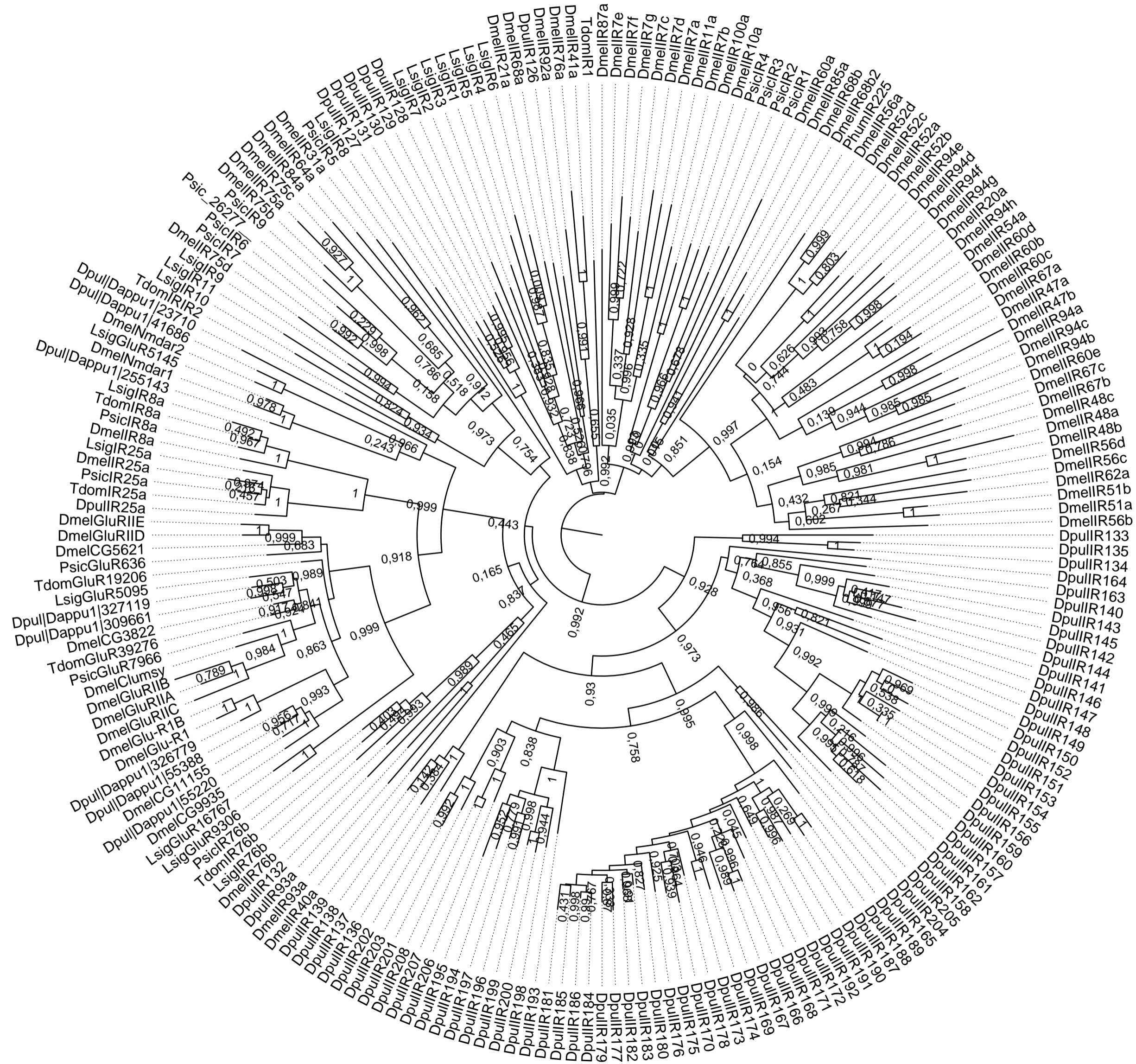

Supplement: Figure 8—source data 5. — DOI: http://dx.doi.org/10.7554/eLife.02115.025 [file elife02115s012.pdf]
