# Supplementary material for: Evolution of insect olfactory receptors
Source: eLife. 2014 Mar 26;3:e02115. doi: 10.7554/eLife.02115 (PMC3966513; doi:10.7554/eLife.02115)
Supplement: Figure 8—source data 1. — DOI: http://dx.doi.org/10.7554/eLife.02115.021 [file elife02115s008.docx]

>LsigIR1_partial_translated mRNA_

KINRNIYENQNKNVLLLFFHFRLLLVHTTRNASSQSIGRNFSFQDEYNLFNLLMKSILISDFSRDNVLCLITDTHYSLDMELLHTYKGTTLNYNVNFSDTPLSLLSWTNRQLRVCQVVIILAYKSHNVLPFLNHTHTMHLFDENTKFLVAYELGERLLQNYLFKKIANAITVQINIEKIKTKFFLESRVPFSEDIVIVNEWYSNTLSFREPLLFLDKIRNFNGETLRVETFSQPPSTIISANEYNQLKTYKGVEPGVIMLLSKAMNFSYQFQDPSDNAKWDTIKRDVEIGRAEIGMANLFIDTKSYLSMSSAYDIDCMSFVIPVPQRSLPWYSILRCFQGTMWTVTSVFFIISVLSTFVLSLCYKCIFGIPPFKLNSVISIIFYHWGIAFGYSNISSVNVRVIRVLLFGWMILSFLLTSAFMSNLIANLSTPSAVQPINSLNELAHSNLIPVSPFISGVLHEVFKNSLDTNLKMIYKRLRSVSDETKRLEGISKGKYAMMFSKNSLLYQKSTNFANKITNFQTHVMNECLVSFHLGLVLQRRSMLRQSVNNQINKIIASGLYSKMKWDVLFPNISYNFRRPIEIDTSAKPFSLKHVQSAFAFLIIGLLLSLIFFVLEVFKARKIYRQTSNYE*

>LsigIR2_partial_translated mRNA

MIFDIACTTFMVPHIRNEYKWYSILTIFDICTWAAILFTVVMCAVVFYLLLRAQTNDPENKTASYVFSLFHSYSRLLGTSVIKKLPSRQARMFDTLWIFISLVLASVFVSKLVAVLTHPSAAHQINTLEKLLNSGIQPACQATMFLRHILATRLDVHAKQLSNKMVGFNNVSQALKLVSDGQLAYLDLSSYLEYEMNSQFSNSGKISVHLMKTCATTSMLSFILKRKSVMRNTLDKIILRTVNSGLYVKWQTDTKSVVQLSSSYFTDNSETANILSLQHVKSVFVILLVGLLIATLALVLEVVNSKKFKN*

>LsigIR3_partial_translated mRNA_

SNILARFLIFYEGECLWLNDPIFKKVPNIAVAITREDIGFSQIDIKTRAIFSKTVYDSVIMDIESWDGKSFTKNCSTLYEDKTSNWYGKHLRVTTFHSPPDVIVSETHPKIYSGMEMEIFNIIVKTLNISYTFQGPPEGWLWGWKLDNGTWTGTMGDLVYNITDIGLVSFFVDKLTIEALDCTTPYDGTCITFLTPAPKEMLHWWSIYRSYHLETWCLIGVVVLIFLGFDYSVHNHLWIQSTLSSFGYSVVHVFGFITGSSGNFNSQFKSMKCLNVFWSFIFTVFTAAFVSKFISLMTTPIPESAINSLEDLLTSGLPYGEIESDYYTQILLESTDDVTKKVAGNMLVVQKDVDGALQGVSDRIISVMESSTLLEYYASNHFTDGDGHCSVHLMKECLIYLGVSIGLPRKSLLKYNADIIITHLVHAGMVSKWQWDIKYPKLPYKSAVPRNNTSHPRSLNIGHLQGIFMFWSIGIIFSIFVFILEYQLNLRKQRKISQYIVSHSGPTEEPQ*

>LsigIR4_partial_translatedmRNA

YAFRLTNPSKFECYVFVTPVPQRKFYWYSLFTSFSLFLWLVSCLVFLITSLFIYFISNWNTIFKSSTIKIISFGDSLFQCIRILLANYSDLTIFRKPTRIFIFCFQVFCLLLTTGFLANLKSILTTPTGELPINSLKELSESNLNYSSYLQLPFQIFNVSSDKYVKSIASKLNYIDPKDVIHLLLEGKTAVIMSYYDFVYFVLFSKSNVNIWERIHFMDGCIYTMPYTYIMPLKSIFVNTVNGRINNLHSSGVLQKLVREEILSSENELRK

>LsigIR5_partial_translated mRNA

AKKLILKTRNELFTSSLLNPKIAVVHEWSEHSSLSHKNTLFLEKALNLDGQHLNLVDVIHRDKADHKIWTILAKNMNFTFTVKPPTHSKGHSGAVLDLILGISEAYVSSVMSEPALYFIIQNTLPNSKRCQTFVTPVPKRTIHWYSIFITFDMYSWVATGLCLIFTTISVCIISKLSPKQPNSTLAHIIKFVRDILFTCVGILLQNGFSFTKLTRPARVVLLSWVILSILLTTAFVANLKALLTTSTGGSPINSFEALSKSKLKILTYSDYFINNLNASSDPNIKDIAKKMTKINKVEMIPQLLFEGNTALLTSLNEIVELSNFYNSSMDILGAV

>LsigIR6_partial_translated mRNA

MFLVVTDSSNCLLDRIFLKVPNVVVAGRNAESISLKLQTRDLFPSSSSVNSSILTVKHWNGTIFTEDNKVTFAEKGSNFNNHTINIVEASNFNRKLDKKIWSIVSQSMNFTIQYKYPKYGDTIIAAMHDLIFGISDGTATTAFINTQAFYLIDISIPMSIKCYTFVTPFPERDLHWHSFLKSFDMYSWIATLTSIMLASIVIYLIVILMQRLHGTHRRISFADSFFGITSLILQNDSNFKPTQSSTRTFIINWAFCSLLITTAYQANLKAVLTTPTGQLPINSPKDIARSGLNCVAYQQIFRELLNHSSNKYLNVIGQNLQIKNRSESLKLLFEGHAALISTAQDMPGQVEALASVHFMDMCLYSPVFHFLLPLRSILKNVVNNKLRHLLDSGIIRKLEWDYLGFDKVPYGTIFQSGEIKSPEPYKLKNVLGVFFIWAFGISVAAIAFAVEIIYVTTKSQVNALIIQRNKK*

>LsigIR7_partial_translated mRNA

GKQWYQRRVFECGIYSDVYKIGEWNGTQFKPSLSQLFSHKICNLRGQELKVCVSNWPPFLDVKRDEFGRRTYDGLNVQLLEYLSNKLNFTYSYKLPTNGNTWGGMKENGIVPGILGDMYHEFSDIGIGGLYLDHLGYNFSNIIIYEGDTFVFVTPTQKEIVKWYSFVLPLTYEVWILIFLFMIVIIVSVLVFTVIYSKSIYSNNLPSTHKQDIIFISLSVLVNNVFKVQLRGNSTRIVIFCWVYASVVLSTGYISKLVSVLTISKSPPQLDNLEQLYLSNIKCATSDVKYFGAILKGMNNAFAEKIRRNLKQVNHLSEGVQKIAEENIAYLESCSVLRYYSKLQYPYKKFHIMGQRFGFFYLGIGVKFKSVLLYDIEAFIRKLVESGIMTHLKKKKKKKKKYSALIPLLAL*

>LsigIR8_partial_translated mRNA

MKVFHITNRKDNLLEKMAFQANFLTIFEIIALYTYTVCGNVEISDAYNGSFSQRYMVKFLLDFAPLWKYDSVYISVHISGKHLEREIGNNIRKMSKLYLPSMGIQLPVTNCTTRASEILQKTTMIVWSGVEELETIQEVSNCITSSGRWIFILNGNQNVNQIFQKAKLPYKSQVFAVQLHNGKRDKYQIDEVYRTHYTNSLAVNRVGMWSPDNGVFLNSKRVLKRYANLRGAPFKVSSHGYYPYVVKQNETIEGISGREKVIWDILQEAINFTYVYVPPTDAGFGSLKDDGHWSGIVGMLERREIDIGMTILSLKRSRMEAIAFSTPLAATRYGFLIREPTFLYLWDGFIYPLSGMFWACLGIIIIVITFITYATYILRQCTRIRNDTEIRNLGFTGILFNILGTVCYQGKYRNAQLKYKMSVK*

>LsigIR9_partial_translated mRNA

SLSREAKLPFEICVRLIVFFQTCRSCFLFRQPSLSSVSNIFTLPFTLFVWLCTISLLLVIAFALFLSVNVEKHYSNEDQEADYSWNEILLVALGAICQQGSPWEPRSFSARITFLFLFVLAVFLYNSYAACIVSLLQSTSTTVRSVKDLIDSPVKLAIHDTNYTRMYLETSNDPVAKRMVKRRKPRYLDLSDGMNRVRRGMFAFHVATIDGYTEMEQQFTDDEKCGLLELPLYRAEMLALPLPRNSPYKEFFNQGLLRIVTSGVRSREFTRWVSQRPNCLQDGGGYVSVGFLEFKPALAALGYGILFSFLFLVGEVLFQRRHKILQCKFLERSPPSQGELFRRLPSPKQFARSYQLPYP*

>LsigIR10_partial_translated mRNA

TWGSLLTFAEDTNVAVRNGQPQDDFAKWRFPPDLLKSIHTFAFINKFCILLPQRYFEDSRLMLHLKDVARLLSRWKIPFTFQSVSNGTADKINYHTTPNLGFLVYEEESHLSQNLATAIESTTTSPKWIFTMAEETEVRSYFDGVYITLRSNVQVFQKQDGGPGYNIFSVYRVDKSHQLRIIELGEWISSNEFNCDLLHIYELRHNLELVDYKGLQRTISDMPDEIRKHYISNSVGLALMLMETFNITIYSSAQLQFYETMGKRDSNGKWDGAYGLMQSGESDFMTTPMPLTKEVYEFAAPACPQLYLSYRLYIKKPSTSFHDWISIVKPLSRNLWTAFLANSFLVAVCLAVVKMIFQKIHIEDTEGSTMSTITYSILSILAGWTGQGVESLSKSATIRILVFSNLIAAVVIYIAYSSELLSNLSVLTLQLPFYSLEELVELSKRHGRRT

>LsigIR11_partial_translated mRNA

KVCFKFALTPVFHAWNQQNIQAIVSADSFGLPLGNGSYDGGMGMLERGEGDMILQAVLYNGDFVGSIDYTSTVFKGWYQMYIHTPSSEFGWATGFVNEFSVGLWRSYGLLALVLTVSLAAATKIGRALKVESSISSQNMLESLTCVSGATCNQGPDIQLQALGPRIILFSTFLAFSVLYLAYSSELMSTLTSKTIDIPFHNLDDLVNLKKGRFVLVMENDSLPHNKYMKDNSNVFKRARENLETKIVEDLTVTEETCSSKRNVLMYVFDLRQQAAIIGCDLDKLPKKYFENPAVFPVRKNMFWLPVMNRMSSNISQE

>LsigIR25a_translated full ORF

MIVRGRLTLLQQMLLVCISLLLFVIHPIHSAQTINVLFVGEKKDTNAEKAFDAVLSQFRSDPKLEVTVGKEARVYADSANTTILADKFCKEYDALIDGDQPLHLVFDSTTAGLTSEAIKYITKTLGLPTVSTSFGQENDIRQWRNLDQEANKYLVQIMPPADVIPMIVRSLADTLNLTNAAVLFDESYVMDHKYKSLLQNLPTRHVISTIDDDVKAQLWNLRRVDVVNYFVMGNAASAQKVLDSADDMKYFGKKHAWYIFTQDSRDTVQCKCANASLVIGRPEPDPGSVNNLQLLKQNYNLGGPKPDIEVAFYFDVITTAFMAVRNMLNREEWPAAENMKFVKCDDHNSDTAITRTGLDLRNALKQIQRPSNSYGKLKIDENGQSYMEFQVKLERLDIIKGEPKGGKVAGTWQVGIPGTLTYADGQDLTELASKTTYRVVTVVQPPFVMAIGEGEDKKFVGYCIDLLNDIKELLQFDYEIYEAPDKKFGAMNDQNQWNGMVKELMEKRADVALSSLSVMAERESVIDFTVPYYDLVGITILMLKPKVPTSLFKFLTVLENDVWLCILAAYFFTSFLMWVFDRWSPYSYQNNREKYKDDEEKREFNLKECLWFCMTSLTPQGGGEAPKNLSGRLVAATWWLFGFIIIASYTANLAAFLTVSRLDTPVESLDDLSKQYKIQYAPVNGSATATYFERMAYIENKFYEIWKDMSLNDSLSEVERAKLAVWDYPVSDKYTKMWQAMKESGLPANFDEALARVRKSKSSSEGFAFLGDATDIRYQILTNCDLQMVGEEFSRKPYAVAVQQGSPLKDQFNNAILQLLNKRQLEKFKEFWWRRPKNLSECTDDDNQSDGISIENIGGVFIVIFVGIGLAIITLAFEYWWYRFKKGGSKVVDVQKINVREFPTNKDNFGGAADATAFNPRPPPATLQDEFRGVGVRFPGNPF*

>LsigIR8a_translated full ORF

MKLHFMCLFCIFLHLSQGQEEEEPAAPAADAKEIKILVVLEEQQQVIADHLTSGFQKAENTVNMNGMKVTIKPIAVSREEEETGFSEVCKDLTTNYNLILDFTWGGWWRVRNMAEGSGMPYLRVDSTIRPFVQAADDWIRTRNGTDAALIFQNEGELDQALYYLIGNSIVRVIVLDRLPGNTTDSLKTMRPSPSYFVMYAKTQEMQKLFSIALKGDIVTRDSRWTLVFLDFEYEGFATSDLPVNVFFIKMQKKVCCQLLDQGTSCSCPADMEIVPNFLLRATSVLTSTLVTLKGEMPDPDPIDCKSVNPEAAKNDTRKRFHEILDMEGKKNPAVGFDPEILLLYYQSEMDITSYTKVNKTEVATWTTEDRFVLRPGYNLTAARRFFRVGIVEAMPWTFKKKDENGKTMFDEKTGMFVYDGYCIELLERLSQTMGFDYEIVLPRDGEYGEKGSKGWTGVVGDLARGETDIVIAALTMTSEREEVVDFVAPYFDQSGISIVLRKPVRKTSLFKFMTVLRLEVWLSIVGALTVTGLMIWVLDRFSPYSAQNNKELYPYPCRMFTLKESFWFALTSFTPQGGGEAPKALSGRTLVAAYWLFVVLMLATFTANLAAFLTVERMQTPVASLDELSRQSKIKYTVVKASTIYQYFENMDHAEKELYKVWKEITLNSTSDQTKFRVWDYPIKEQYGHIKSAIENTGMLDNATEGFTKVLANEKGEFAFIHDAAEIRYEVYRNCNFSEVGEPFAEQPYAIAVQQGSHLQEEISKQILDLQKDRYFEGLSGKYWNSSRRGLCPNLDDSEGITLESLGGVFIATLFGLVLAMFTLAGEIFFHKRKIKHQVHNITKSNALTLASDFRGTEKSVPRVSYISVFPRS*

>LsigIR76b_translated full ORF

MAGSLNFLKTVMMSVCLAGAGKDGTIDPATVTEEYLTEQCNMNRLPKIDFWNLKLGPSKELNGKHLLVATLQDPPLHYTEMDEQGKLVGKGIAFDMLNLLQDHFNFTYEVKEASENYIGSYDAKLKTWNGILGMLYRKEINMSVAFTPITVTAMKDIDFSFGMGEMKWVLMLKRPVESVGGDGLLAPFQTGVWYLILLSLVIMGPLIYGIILFRVKLCRGDARLTRIFPLHQCIWFVYGGLVKQGSTLSPVTDSSRLLFATWWIFIMILTSFYTANLTAYLTLSKTTLAIETKKDLTGSSTKWFVRKGSAIETVITHEKGLEDLAKMVSKGYGSYARADLTDVEIMAMLEKRSVTFIQRANSIERMIFRHYLNQTKNGVPEPKRCTFVYSSSPILKLEYGFGYQMGTPLKRLFDPMVSYLVESGITKHLLQLDLPSREVCPLYRGFQERPMRNEDLFMTYTIVGSGYGLGFLSCLVEMFLLYIWYPKFSKKKKKKKKKSTLR*

>LsigGluR5095_partial_translated mRNA

RTRLVPYFENVPLNNSFKATKHVCSILRQSVVGVFGPQSEELSGPIQSVCDAVEIPHFENHWQIQDTRECCRFNLYPHYEALSRALVDLVTEWEWKTFLLLYDDDDGVVRLQGLLKSLANDKQPFIMRQLPEGNDYRHLLREVMHTGEHNIVIDCSADKIFNVLKQAQQVGMMTAHHSYFLTSLNLHTVDLEDFQYGGTKIVGLRIIDIDDEDIIRTVRHWRYSEMRNSRILNLSAASLKTETALMYDAVHIFAKALHELDNSQFVTIQPMSCETPDPWHYGSSLMNYMRVGEHRGLTGLIRFDREGFRSDFMIDVVQLYENGLRKIGTWNSTEGANLTNIEHKVIQLGPDSLRNKTMIVSTILNEPYTMLKERSEKLTGNDRYEGFAIDLIHEISLILGFKYIIKLAADGKHGSYNDTTNTWNGMVGDLRRGEADLALADLTITAQRESAVEFTMPFMNLGISILYMKPRKQPPNLLSFLSPLSMEVWVYMMVAYVGVSCVLYSLARLSPYEWDNPYPCIEEPEELENQFTLLNSLWFTIGSLMQQGSDIAPKAVSTRMVAGMWWFFTLIMISSYTANLAAFLTVENLESPINSVEELAAQTKIKYGAYKGGSTVKFFQNHPLEFYRKMFEVMKEAKPSVFTSTNQEGVERVEKSNGLYAFFMESSSIEFNAERKCEITQVGGLLDSKGYGIALPKGSQYRSAVNGALLKVQEQGKLHILKSRWWKEKRGGGQCTDDAGGGGAAAELGLANVGGVFVVLVGGMFTACFIAACELLWTTRSVAEEEGTSFGSEVLAELKFVVSCHGSTKPARKKPQEDVNSFLPLNQYQDYGFPNGDEKSQN*

>LsigGluR5145_partial_translated mRNA

MTLEAISIKMEANPIRTALNVCKDIIARQVYAVVVSHPQTGELSPAAVSYTSGFYHIPVIGISSRDSSFSDKNIHVSFLRTVPPYSHQADVWVELLKHFKYKKVVFVHSSDTDGRAILGRFRDTAQSLEDDTEITVEKVVEFEPGLTSFSEHLESISVRSTVYIMFAGKSDAEVIFRDASLLNITGAEYMWIVSEQALAAQNIPEGTLGLSLINATSEAKHIKDSLYVLASALREMYRKENITEAPGDCNDSGAVWETGRQLFEYIKLQVLEGGETGRVAFDDNGDRKYAEYDVVNVRKDGERVVGKYHYDKELKRMSLQLNTSSIVWPGGLTVQPKGITVETHLKVLTIEEKPFVYVREINPSLGEACDASQGEIPCPHFNNTDYEFRLFCCRGYCMDLLRELERKINFTFDLALSPDGQFGSYGPIRNSSGVKEWTGLIGELVKKRADMIVAPLTINPERAQVIEFSKPFKYQGITILEKKPSRSSTLVSFLQPFSNTLWILVMVSVHVVALVLYLLDRFSPFGRFKIGNTEGTEEDALNLSSAIWFAWGVLLNSGIGEGTPRSFS

>LsigGluR16767_partial_translated mRNA

RGVAINGGQWFCRNCQVSMDHFNGYFVVAVAFLVLGPNPSLQQYGRGVAINGTTLRVATLLTTPFCSQNKNGQYDGFVPDVLSELEKQTGASFSLKKASDGKHGVLDKRTNIWNGVIGDVIGGDADIGFADITVTAEREKAIDFTVPFMEADLTIIYRSVLEEPVAFGWLLSAISLPFWLSILACYVIVSAVLYAIGRITPKKGKPEKDTPTDLTISDSFWATTSSVFLRGTDVPVDALSSKLLVSGWWFFSLGVVLILIVLEVSMFTSGAAFTPAAPNFFAHVGSATVGVLGGGSSHRLLLARPDLAPVLRRLEAAGSMGTPSTVAEGVQKVVDGELDGFVIESPSADIMKASHCTLETINEALDMRSFAIAVKQDSTLREVLSIELLKMRESGKLDAIRNRWWPKDRCLDFDQTTLSPARLGTYYMLFVVLFIVIAIALLAALAERYMHSKNNS*

>LsigGluR9306_partial_translated mRNA

SGGSFEDKMIRLKGASLISVVLVFLVLAPKPSILQQIPKALRIGSLFLEDDRGLHDTFRTAIDALKRRPDLIPSTRVDFDIQYANPGDHFHLGKRACLEMQYGVMAIFGPFDNSLDMHLASVTSVLNMPLLSFAPGGPHGSLSISLYPSQYDLASATKDALKFLKWTETGILMDDNGPISSSDLLETIGSLDLSLVPVQAGEDLRESLAELRDRHVHHIIINLAAQRTEEFIEAILGTGMVDEDYQYFFTSLDAHNTDFEHLRFVKAQFYSFRLAPHKDSSFYRHITRESPGLLNSTTQLLAVDALELLARGIRIAADKGADMVTPPRVSCSGEQTWSAGLALHSALQQVKFRGVSGPISFEDGTRNSVDLDLLQLSIHTNVTSFERLGIWSIRPEDNAGSLNITGGFPHAVNRSTLIVTTVLDSPYCQQGDNGEYKGFIPDLLAELERFVGASFQIKHAADNRYGSYVARTKSWNGIIGEVMQGKADIGFGALTVTAEREKAIDFSTPFLEADLTILYRSQTVEPVAFGWLLTPVSWPLWLSLIVSYMLVSIVLFLVGAISPYQRQVAPGSGRCCGSGLSFANSFWVTTSSLFLRGSDVPIVATSARLIVSAWWFFSLGVILILIVLEVFVFTSGAAFMSAVPDFSGHSGSALVGVLRGGSSFRLLQTRQDLMPLLQRAESSGHTGFPKTIDEGIESVLNGNLDGFVIESPTAELAKASHCNLHTIPQVLDKRAFAIAVQQGSPLRDKLSVGLLNLRENGKLELLKTKWWPKGHCKDYEHTSLSPASVEAYYMLFVVLFSTLFFALLVAVAERIFHRRVFSAK

>TdomIR1_partial_translated mRNA

LGAICQQGHYRPPESISARAVFLLIFLVGVILSSVYSSTVISFLTVKKLELPFRNLEELLRLRNYKLGILSGTAAYYILQSTNDAVFNKVMGKLHETEQPLTIPEGLNRVCNERFAFIMSTLSAAMTLPSASCPIVDTSTDYFKTTVSLPMKLRFPYRKIINYYLKRMKEDGVLYRLIITRGLLRTEDSVTEELTSVSLLDVIPLLLLLLLGIILSIFTLAGELSHWHIRSCTDRHIVKPFID*

>TdomIR2_partial_translated mRNA

ALTTILGIVLALWIMMYLQSVLLKRGLIVNNRYNFIELAFNSFRAMFFGHKRPGHPLQESAYPLPSACWFVLLLIIFAYCTGNTSYLTLPIFSDSLHTVDDLMKTNITKGQTKHTSLSYVLRSEDSSVAKFANKFQKEAGIVHRDTKTQENNYAILVKTVGQSYLAGAEDLEEQSHSDLELMGECVSHFYVAFGLQKYSPYKRLFDRALTRFFESGITQFWEKDVAKDLGYHYMREFFSDIVSK*

>TdomIR25a_partial_translated mRNA

VYVETKESGEVEYKGYCIDLINDIKDILKFDFQIRHVDHFGNMDDNGNWNGMIDELVKKKADIALGSLSVMAERENVVDFTVPYYDLVGITILMKKPKAQTSLFKFLTVLENDVWLCILAAYFFTSFLMWVFDRWSPYSYQNNREKYKDDEEKREFNLKECLWFCMTSLTPQGGGEAPKNLSGRLVAATWWLFGFIIIASYTANLAAFLTVSRLDTPIESLDDLSKQYKIQYAPVNGSSAMTYFQRMADIEKRFYEIWKDMSLNDSLSDVERAKLAVWDYPVSDKYTKMWQAMKEAGLPGTFEQALERVRASKSSSEGFAFLGDATDIRYQVLTNCDLQMVGEEFSRKPYAIAVQQGSPLKDQFNNAILQLLNKRKLEKLKETWWNQNPLKKTCEKEDDQSDGISIQNIGGVFIVIFVGIGLACITLAFEYWWYKYKKNPRVLDTTVVHVREQPPNKETLQFNPRYNYQTDFRPQGLNMAGVTNPW*

>TdomIR8a_partial_translated mRNA

MKRDSSGQPIKDQNGKEIWQGYCVDLLDKLAEKMDFDYELVFPESGTYGKRYENGTWDGVIGDLARGETDIVVAALTMTSEREEVVDFVAPYFDQSGISIVIRKPVRKTSLFKFMTVLKLEVWLSIVGALSVTGVMIWILDKYSPYSAQNNKEMYPYPCREFTLKESFWFALTSFTPQGGGEAPKALSGRTLVAAYWLFVVLMLATFTANLAAFLTVERMQTPVASLDELARQSKINYTLMAGTSIEQYFLNMAGAEQELYRVWKEITLNSTSDQTKYRVWDYPIKEQYGHILLAINTTGPVENSSVGFQKVLDSEDGEFAFIHDAMEIKYEVYRNCNLTEVGEPFAEQPYAIAVQQGSHLQEDISKQILDLQKERYFETLSGRYWNASQRGSCPNTDDSEGITLESLGGVFIATLFGLALAMITLAGEVFYHKRKQKNTVKAMNNKPVTIGNEFKPADKMPRVSYISVFPRN*

>TdomIR76b_partial_translated mRNA

MALNKKLLEMLFLICLKQPGKPPTEDAAELESWLDHECYNDELKEYYWIRNHNYTVAALQDNWPLSVVTQDAQGNLVGEGVAFDFLNILKKKYGFNYKIKTPKEDIIGDKTTGIVGMLMRKEVDFAAVFMPVYDDYRDEIEYSVNIAEDRFAILMKRPQDSATGSGLLAPFQTEVWFLILLSLIVMGPIIYFIILIRVKLCKGSARLTRIFPLQSCIWFVYGALLKQGSTLNPITDSSRLLFATWWIFITILTSFYTANLTAFLTLSQFTLPIKTAKHVHEKHMYWVAKTGGPMEHWIKFDETFDYLNNSRYGVFASSPDPMIMEKEIMKQGRVYFRENRIIQDLLYRDYLNKTRRNVDENKRCTFVATPKPFLSRPLAFGYPKGSNLPPLFNPILLSLVEAGIVHHKLHELLPGTDICPLNLGTKERKLRNSDLYTTYVVVCGGFSSAAVAFFGELILKLIKHCKNKDKKKVPLKDDVLYNPKHQFVYGGTTRDLEMGEDYKSKQINGRDYFVINAKDGGQRLIPVRTPSAFLFQYSA*

>TdomGlu19206_partial_translated mRNA

DNQGFRTNFALDIIELSTDGLQKVGTWNPSKGVNMTRTSRPAPTEESSMFNRTFIVTMALSDPYCMVKEASTMLTGNDRYEGYAIELIHEISQILGFNYTFKEVDDKRYGNWDNKTKQWTGMIKELLEGTADLAITDLTITSERESAVDFTMPFMNLGISILYRKPTKEPPSLFSFMSPFSMEVWIYMMAAYMGVSMLLFILARCSPDEWNNPYPCIEEPEILENQFTFKNSLWFTIGALMQQGSDIAPTAISTRMVA

>TdomGluR39276_partial_translated mRNA

MYDAVRLFAKALYDLDSSHPINVMPMNCDGTTQWPHGRSIVNYMKIVEMEGLTGPIILDNNGYRTDFSLDIIELYKEGFRKIGTWHSLSGVSLIRTYNDTLVEIGENLQGKILHVVSLPSKPYCMEKNETDANGNIEYEGFAVDLINEIAEMLNFTAQFSIVADKKHGSRQENGEWNGMIGELLKRRADLAIADLTITYVREQAVDFTMPFMNLGISILYKKPVKQPPNLFSFLSPLSVEVWIYMATAYLGVSVLLFVLARITPYEWQNPHPCNSDPEELENQFNLLNCMWFAIGSLMQQGCDFLPKAISTRMVAGMWWFFTLIMISSYTANLAAFLTMERMDNPIESAEDLAKQNKIKYGSVKGGSTMGFFKNSKFPTYQRMWSVMDSERPSVFTSSNQEGVERVKNEDGTYAFFMESTSIEYVVERNCELQQVGGLLDSKGYGIAMPPNSPYRTLISGAVLRLQEQGKLQQLKNKWWKSEGTCKDDKKSATSTSELGLANVGGVFVVLIAGMFAALVIGICEFVLKSHKTAVEERTSWWEEMSSELNFALRCRGS

>PsicIR1_partial_translated mRNA

TEVVFSLLSAAELTNVNVLMVNVGAPEQLGLEDLDLKNIHSTPVPVLTTSSSFHGEKFRFFERGDTTANSIAYVFVRKHLTELCFVESVSTSYKWSSRAYFVLIVTFPLRDARARISAMLRHCWESCNILNVLVVFHGETSDKNMLSSQKRHFQMYTYNPFKSNGGAGDKLKALTPETCMDACRRKLNNANGYVFDVSASPHHPLLWELVDASGRQTLGGPDGEIMSAVIQKLNGSLRFFRTAKDFFSDSKRHTKVLMANERVVLRSMLRNSIYPRQQACITCIVPKATRVPSYMSIAMPFSPGTWLLCLSSGVVSGAAWKLVTRHDRREPMTRTFVDVVRLVVLGVAGRLPVSDSQRLLVLWCLVGNLVMTSLYQGFLG

>PsicIR2_translated_full ORF

MKKSSLVEYALIVLMHSGIVLSMNSAIFNRRDFGLVEALVDYAKQVFDEHLRRRLLLVNYDSSTRTEVANEIVRYFNNVDQPLITTMVPPKTGRNDEVRTSARTLASHSRQESSPLYRHDNVESSISFVVFRDTLTGTCFANEAWIRTPVWNSRARFLLIITSPSAENFTEITELFRECWKKANLLNLAVVYLASPSVCNGGEQYSIRAKELNPFIDPNCVMNVTVSGTRRKREHDMFQEKLGNLFGYEIPVFFNHSHPFLEVRYEQGGELAFDGPDGHIFNTLLAKFNFTAMPATNISSDFRIEDLSPEEKHYYKNGLNANEEALLPSNINRSSYPFYHDYISVIVPKAFPLPRYMHAILPFTQGMWALYIFSLISLCAAGATLNYFDIHEHVQVRSSVFEAFQLLITGTANIRRLSFVQRLFLTVCFVFTIVVNNAYQGSMTSVLTKTLYQPDLNTYEDILKSDLRIAVFFDSSYAAETSKDLETNDSDDDDFVERVVDKMEFIADDHEALMMAAIHRNVCRPTYRFLAEILIEHEDFYNAGEPLLHIAEEKSLPLLEVWSPKHRDSVFLEKISRISTNLVTGGMIEKWRSDMQYAELISRHRRKTSFRTGIKLTLEHLQAAFFILGIGLLIAVLMFVAETVFSLTDVQLCFVTLKRFLSSSK*

>PsicIR3_partial_translated mRNA

VAVYVINERTTWPASMPQESTAFVILSQSGEVDVYMNHLAQVCRLPLWTSWSQVFFIDANLKDNHKDISFALLKASWKKFEILNCLVIYSATCKDKSRKLSVAKSLNPFVSPGRTINVKYKDDLFEKFPFDCMGYPVKCTAFNYPPFVFEETTQNGIKKLGGMIGTIINIIAMSANATTTTDVTTLKDTSKWVLPYSFYNTTDVIYPFAPLQTSNLKESSYPFLHDKLVLLVPKAEQIPKRMCAILPFNYEVWICYLGVYVLLVLLGKVYSRYTRGNTLALFSSLEIFRLFFTGMSQQRVTAIGLRVYLASVMLFNIVLNNAYSGSFTSYLSIPQYYSDINSFADIDRYCLSIGLSLKYGTDAALDFSSIVSQLDTDDKMTAKFLYRLEMVESLEEGVHRVKTERNFCLLHFRTGAKFEISKPENIAGNRPLLHIAKQELLPLHAVSIMRFNFPFLYSSKSRQRLVIESGMVNMLLVNFDRELLKTHENNDVVVLNLHHMCIFFIILFSGCLISIIVFACELIMGKIAFKNIFSNGNKRT*

>PsicIR4_translated_full ORF

MQLVFFVLLLLVVATLNGSVITGRNRYERRAQQFVMEATSVIHEYLRDVAGTMVLYTEDHQGTTQDLIISYLYSTTKQPLLVFKNTAQCCPPIASQSAGFIILSARRDVHAFIQSLVHASGLPVWRSWSPVIFVDTNLNETRSDITFPLLKTSWKKYETLNSLVICVSANSDDSYRHVVVKRFNPFVTAGKTVTVTNKQLTTQKLFFNARGYPIKCPAFNYRPLMYEVTRPNGEKALGGIIGIFADMISELGNFTTTTQQDTLKNQFQWVLPESFFHERFDVKYPITILQPDILNNSLYPYMIDNLLLIVPKAQQVPKWMTAVLPFTYTLWILYLSCYPILVVLGTIYSRISINKSKGSFHALEIFRVFFLGMVHHELKFVWFRLYLASVMIFNIVLNNAYTGSFTSYISIPHYYKDINTLEEVFHQGFSVGIAVKYGSNVNFNYTSMISDFDFNDELTYMLMSKSMTVTSTEDFLISAITNRNLSLFHASVAGMYEMSKPIYFRDGRPLLHMIPQSFLSLYLVFEHRKNYPFANILDRNLQLVVDGGFITKHISDSIEMVHTSFHSFSKDFTSLNTEHLQSFFYLLLCGYIISTVVFVVEVYYL*

>PsicIR5_partial_translated mRNA

MGHILWHQNDQPNKRLNDDYNFSNFEETVGFPAHDSNMKAFQYIQELCRFKTTLKITEELASLSVTDNDGIVGMLQREEFDVYATLSGILPVTFQYVDFLQPITQFQPVIVFLQPTVPTLRNVFLLPFTRNAWLVLVGTVVLSTGALLVSTTLADYGQHWGVSDVVLIVLGIICQQCMEQLPRSLAARAVLGVLMLSTILVWGTYSGVVISLF

>PsicIR6_translated_full ORF

MQLRDVYRPGPGVPHVDRPAGMWTRSRGLVHELTAYKYLRREDLAGLRLRTGITINAPLENPEVNLLKLENRQLDAMATYNYGLYIMLQQQFNFTMELVVTDKFGFVVEDGRYDGLTQLLGERQVDTAISTLLMNRPRMSFVDYSTGYGWEFHICAIFRHPSVSGGEDALIKPFSGSTWLCTFLMWLVIAAFLKLLAWIQPHFADVTGEDHEEIPSWSDLMLLVVGIVGEQGTWLDSKWFTWRFVFFMMLLLTVLLNTFYAAIVVSTLLNQQAQTINTARDLLDSHLHFGAEDIIYNRPYFEVNSDRLIQELYKKKMAGHTSYFPMEKGVAKVLNEEFAFHTEAVRAYPVIEKMFPDEKKCALKEIEFFPVEMGFFAFPFMSPYKKMFTYGLRKIAQTGLWQHQNQEWRSSRPTCASTGAEVISVALPSLAPAFGLLALGCALSLAVLVLENLQHASRHRPRFRTSDKQLT*

>PsicIR7_partial_translated mRNA

PFSGALWLGMFSLWLLIVSFLRLLAWLQPQYADITGENHEDIPTSSDLFLLVVGIVGEQGTWLDSRWLTWRFVLLMMLMLTVLLNTYYAANVVSTLLNQSPFTIRTLKNLIDSSLQFAAEDVIYSRSYFEITNNSLIRKLYTKKMAPWGGNYISMEKGLERVQNGDFAYHMEDVRAYWAIEQLFPDSKKCALREIELFPGEMGYYVYPFNSPYKKIFTYGLRRVVEVGVWD

>PsicIR8_partial_translated mRNA

QGSDLDSKWITWRLVFATMLMLTVLLNNYYDACMVSWLLSETPRTITDLEKLVHSPLHFGCENLSYIIPYFVNNDDALMRELYTKKMLRERGVEGAFYPPQVGVRKMLEERFAFHADPNHMYGMIEATFPEDDRCDLTELQIMPPRSHHLALPSRSPYKEIFNYGVSRLKETGVMDYTWKHTRPTRTKCYSLPRVRSMELSYTAPGFTMLLIGALLSLVVFAGEFLWHQREKHRDSKIKLFIETSD*

>PsicIR25a_translated_full ORF

MAAMYAFLAGLTLLGVAYAADINIMFVNDEGNVVADKAVEAALEYLKKNGGGNFKVDKVSGNGTDAHDFLDKLCKVYNDSLEASQPPHLVLDTTISGVPSEVLKSFTYALALPTISASHGQEGDLRQWRNLDPEKEKYLIQIMPPADMLPEVIRSLAYTQNISNAAVLFDDSIVMDHKYKSLLQNMPTRHVIVKAGENMGIKEQLAKLRGLDLFNFFVVGRLSTIKKVLDFANINKFFATHFAWHAITLESGNLKCSCADANILFLQPEPEADFKDRVVQLKNEFGLDEKPEITAAFYFDFAVNALSAIKSMVDGGTFPADMKYVTCDEYTEETAPNRTGVELMEALRSVNKAPSYAPVRLENNGHSFMEFTLRITKANILNSKLETSEEIGTWKAGFDEPLQVTDEGILSNMTAITFYRIVTVEQKPFIIKDGVDEKGRPKFKGYCIDLINEIRNITEFEYEIFESPDGKMGNMNEKGQWDGMIKELMEKRADIALGSLAVMAERENVIDFTVPYYDLVGITVLMKKPKAPTSLFKFLTVLEKDVWLCILAAYFFTSFLMWVFDRWSPYSYQNNREKYKDDDEKREFNLKECLWFCMTSLTPQGGGEAPKNLSGRLVAATWWLFGFIIIASYTANLAAFLTVSRLDTPVESLEDLSKQYKIQYAPLNGSSAMTYFQRMADIENRFYEIWKDMSLNDSLTDVERAKLAVWDYPVSDKYTKIWQAMKEAKFPNNMEEAMDRVMASKSSSEGFAFIGDATDIRYLVLTNCDLQMVGEEFSRKPYAIAVQQGSPLKDLFNNAILQLLNKRKLEKLKEIWWNQNPEKKTDCEKSDDQSDGISIQNIGGVFIVIFVGIGMACITLAFEYWWYKYRKDPKVVDVTAQPAVIRQSVAPSKIDTGITMGFRPRQPYPDNFRGHGMPLAGVANPW*

>PsicIR8a_translated_full ORF

MLPYVVVYALALELVTAQKTFKILVVTEENQEQVLDMVAAGLKSAEQAYDGVRFDQVPVPVDRENEDESFEQVCKQLAAGVSAVLDITWTGWDKVRTTADSAALPYFRADVTPGAFVDAVDAYLGRRQATDAALIFQNEQELDQTLYYMIGRSIIRVIVLDGLEGNATERLLAMRPSPSYYVVYATTTNITKLYDKAVEGNLVTREERWLLVFTDFSHQQFDRSSLQLSAVLFTMRTDVCCRLADQPAGCRCDNHLQVLPSYLRHVVLFLGRLASHLDQHGVEVGPRPVQDCAAHAADTDAANTKFYSNVSQVMATMGERRAIGFAPGERLLYLRGDMEITMANSTHQQRQGTFSRENQLVLEHNVTLQSGRRFFRIGTTESIPWSYMARDETTGALLLDEKGRPTWDGYCIDLLRDLAETLQFDYEIVPPKDGSFGSRRPDGSWTGMVGDLATGETDMIIAPLTMTSEREEVIDFVAPYFDQSGISIVIRKPVRKTSLFKFMTVLRLEVWLSIVGALTVTGIMIWFLDKYSPYSAQNNKAMYPYPCREFTLKESFWFALTSFTPQGGGEAPKALSARTLVAAYWLFVVLMLATFTANLAAFLTVERMKSPVASLEQLAKQSRINYTVVKDSDTHEYFKNMKNAEDVLYNVWKDITLNSTSDQSKYRVWDYPIKEQYGHILQAITQAGPVENITVGFQKVIAEEDGKFAFIHDAAQIRYEVSRNCNLTEVGEMFAEQPYAIAVQQGSHLALEISRRILDLQKDRYFETLSGKYWNSSAKGLCPDTDDSEGITLESLGGVFIATLFGLALAMVTLAGEIFYYKRKKNTAVSVQKPGNFKDALSAKKQVTIGKEFRPVVGDKVMPRVSYISVFPRNQLY*

>PsicIR76b_translated_full ORF

MQSLIPLLMMNVCSNYIGENTTKVQDADDDEEVVDPGCILRDPPLLTHIHLKIATITDHPLSYVVEENGRKVGKGVVFDIVEILRSKFGFTYEVVQPRENSIGDNNTGLLSMIHRGEADMAAYFLPIVWEKNHGVRYSFSLGDVDWVVMMRRPTESANGSGLFAPFDTTVWLLILVSLILTGPVIYLIILVRVRLCKGSERLTRIYPLDACIWFVYGALMKQGSTLSPITDSSRLLFATWWIFITILTSFYTANLTAFLTLSRFTLPIDDAYDMAVYRYRWMAQKGLTMHEVVRYDPGYYYLNDSLKAGRGEFLRGDNAKMMAIVQKENKMFLRERNVVEYLILRDYVTKTHKGIEETKRCTFVATPKAFMERSIAFAYHPKSTLYKLFDPVFMGLVEQGIVKHLLRRGLPKNEICPLNLGSKERQLRNSDLFMTYLIVMSGYAIAISVFVGELIVRAAKKFNDSRLINTHDNGYVPSKSHMFPPPYSTVLIGVDAEGGKKQSINGRDYLVVNAKDGDRRLIPMRTPSAFLFQYSA*

>PsicGluR636_partial_translated mRNA

VKLTPGGLKQVGNWHSTEGLNISATIEEEPLVTDDNTLRNKTLIVLLSLTKPYVMTKISKDALFGNERYEGFCIDLIKELAGMLHFNYTFVLHYDSNYGGIKNGEWTGMIRRIMDGEADLATTDLTITAERESGADFTMPFMNLGISILYKKPQRAAPELFSFMAPFSMGVWGCMLSVYVGTSVLLFIMGRICPYEWANPYPCIEEPDELENQFSLGNSLWFTIGSLLQQGSEIAPMAVSTRMVAGMWWFFTLIMVSSYTANLAAFLTVETTFSPFTDVKSLAEQKTVKYGAKVNGATFAFFKESTNPVYQKMYKYMEENKADVMLMTNEDGEERVKSEGEDYAFLMESSSIDYTVERNCELTRVGGLLDNKTLIIPVEHSCSHAQTPVSMLEVVELHRKNKAALHFVVFTPDFIHVAQANVMK*

>PsicGluR7966_partial_translated mRNA

KDILLELMEVSQAKLRKIGNWTVYGGITQEKDYSEQVSLEARQMMQNKTFIVASKIGPPYLGWKNRSAVGNERFEGFSLDLIDAVAKFNNFKSYEFVIVADNQHGTQDAETGQWNGIMGEVIGRRADMGICDLTITYSRGSAVDFSAPFMNLGVSFLLTKPTKDPPEMFSFFFPFSFDVWIYMATAFLGVTLILFVLCRITPHEWDNPNPMDDDPEELENTFNLMNCLWFSIGSLMAQGCDLLPRAVSTRMAAGMWWFFTLIITSSYTANLAAFLTNNRIDDNIQSAEDLADQTSVKFGSLRGGSTAQFFASSNYTVYQRISTMMKQAKPDVFTSSNQEGVDRVLKEKGKYAFFMESTSIEYETERHCELTQINGLLDSKGYGIALPFNSPYRTFVSEAVLKLSETGKIKDIKDKWWLVKDGTGCSEIETEKVNNDELKMANVGGVFLVLIVGCFAAFFVSILEMLWNCRKIAVEEKITPCEALISELKFAVNLSQTTKPNRKKKSRKSGGSSSSSVSGSVLVDASHD*

**Additional contigs**

>Lsig106170_putative variant iGluR_partial_translated mRNA

KFVVCLTSNSSGKEELQELKFWKIVNSVVVTATSCDGDQKFVFQLLSYQPFPKSDESSLYVVDHCTVENEDVTFESQNPVRNTQYFPSKTSNLNGHEVYISTLNHLPYMKWGNTDKPTAGVEFNLIETLGKQLNFKPKYTTQSDGLKWGSELKNGTLIGMMGDVSYQRADV

>TdomIR25a_2_partial_translated mRNA

MWSSFLFLLVLHVAWVHSQQNLNILFVYDEKNTIAEKAVGVAQDYLRRQNKYGVTINNFDTVVVTNSSDARGLLDKLCKAYNDSIAAGKPPDLVLDTTMTGITSETVKSFTNALALPTISTSYGQEGDLRQWRDLDSEKQKYLIQIMPPGDLMPQVVRSIVAAQNISNAAILFDDSFVMDHKYKSLLQNMPTRHVITMVDKDPKKQLTRLRDVDIVNFFVLGNAPTIKKVLEAGAYKGYFDRKFAWYAISKDETALECNTCQNASVGVLKPVINPDYKNNFGELKSEYKLDMKPEIDAAFYFDVAVRSFIAVKTMKDRGEWPTDMKYITCDEYDDNTAPQRTNIDLKKAFKEVTDPKPSYGNLKIDRNGFSFMEFTMPLRKKRFVGGQGQTAADLAVWEASIEGPIKIK

>TdomIR8a_2_partial_translated mRNA

TDDSEGITLESLGGVFIATLFGLALAMITLAGEVFYHKRKQKNTVKAMNNKPVTIGNEFKPADKMPRVSYISVFPRN*

>TdomIR8a_3_partial_translated mRNA

MASGKYPQILIGLLLVCCVRCQSPIRFVAVTESHQQAIANILGQSVKKAEESHDLKLDQVTIPVSREGEDEGYENLCQETKKGVSVILDFTWTGWYRSMKLADDAGIPYIRLDATVRPFVEAMEAYLKKRNGTDAALIFQNEAELDQTLYYLIGHSILRVIVLNRLQDNTTLGLRKMRPTPSYFVIFADTDNMKELFNTAVNGQLVTRDSRWNLVFTDFNYDKFDK

>TdomIR76b_2_partial_translated mRNA

LIDREIKRWFILKPLCLNGDAAVFNSIDLQDVIPALYLFGCGALAAVIIFLLELILHYSIILHQIISNRRSHKDEDLHLRRITTLNREKPKQTKIKPSKRMQFTKQRYGNQ*

>TdomIR93a_partial_translated mRNA

LSRVLLFMYPFTTDSWLCIAVSVILMGPILNYVHRRSLNYRFQCKKTSGGLFRITNCYWYVYGALLQQGGMHLPETDSGRLVIGTWWLVVLVIVTTYCGNLVAFLTFPKIENPVESVDDLLKRKGTITWGLLKDS

> Tdom_21471_putative variant iGluR_partial_translated mRNA

STNDAVFNKVMGKLHETEQPLTIPEGLNRVCNERFAFIMSTLSAAMTLPSASCPIVDTSTDYFKTTVSLPMKLRFPYRKIINYYLKRMKEDGVLYRLIITRGLLRTEDSVTEELTSVSLLDVIPLLLLLLLGIILSIFTLAGELSHWHIRSCTDRHIVKPFID*

> Tdom_34097_putative variant iGluR_partial_translated mRNA

LMWNQDSSDFKPVAKEILKDMFRSYRCVHLLTDKGTMSSDIMAATYLSVSVPVINIEVTPRSDGSPKEWIDLITEGQSENCNGYILALKNPRTLAMEYGRNFFLTPGNRVVAFSDEDIENKDVSVGCPAFYETSEVITVSRKINSTGTTFTVSLPTKQDTPVGFWRKETGLVTYGSLFPREPTDLRGRPIRVSGFHCPPFVIYKGSGKSRILQDGIETRFLSELAKTFNFTWKIVHERDEEKWGKTLPNGTITGVTGDLIR

>Tdom_39421_putative variant iGluR_partial_translated mRNA

TYDTKYNKTFYGLLSDDNYHVGIPSSYIRFDVLENFDGSVPFLETSLTFLTPKPGKKSSILSIFEPFNEVLWMALGCL

>Tdom_ 42947_putative variant iGluR_partial_translated mRNA

LELSVNKNETNEFARELYRKKMEPHLSNVFFEKEEGLQSVVKGQMAFITDTNEAITYLQETLTEEEKCSVAT

>Tdom_64008_putative variant iGluR_partial_translated mRNA

LFTWIILSTAVYCISTLNYKIGSNKARVVDLSEAHLIVLGNLCSQGSPIHATFWSGRIVHITAVLFGYLIYNSYSSIMMSKITAK

>Tdom_71755_putative variant iGluR_partial_translated mRNA

VGAFCQQGTARDPVTAAARILFIALFVLAVLLYTAYSGNVVSLLSATGSVTNTQQQIINDKMMVGAEKTEYFLSYFKVC*

>Tdom_82603_putative variant iGluR_partial_translated mRNA

AVNESMRHRLLEQLLTQILRFMDKLSCLCIITDSIYSSLLHASFFATLEYRPFFKVAVKDNEDLINPHYTTLAAIEQVRKDGCNGYIILIAN

>Tdom_86455_putative variant iGluR_partial_translated mRNA

MDTNSPDNILILADSLQQFVSFFNKFKVIDVHSKIIIIFTDETIDKTKIRIILELMMKLRLVNTIIAISTNNETLWYTYFPYSVNFCEEYVEKILMGYCKNGYYHSNGKLFPDKILNMH

>Tdom_88427_putative variant iGluR_partial_translated mRNA

YNLSWNLHFTNPDWGTSEDLSSGETFYSLLLDTKFQAGIPSGYILAPALEYYDVSIPFLQTSISFLTPKPQRKSFLLSVFQ

>Tdom_89771_putative variant iGluR_partial_translated mRNA

IIATPATNVTDTKNLTYKVQFSTFTVDDYLNPGNPFRIDVWDSGSFINNVDLFVDKLSNLQRHIILACFMEYEPYTILLNETIHEGLDLRIMK

>Tdom_93549_putative variant iGluR_partial_translated mRNA

QCGKAIRVQLLNTCQEIYRERTTIFPDKTRNMGGCPLVVGTAEIAPQVILKQNDDNSTRVSGIEGKLIRIIG

>Tdom_94268_putative variant iGluR_partial_translated mRNA

CGYVTDLVILRECTKTRNFKESMQLESEFTNLNKCPLRLSTFDFLPYTQINDKRTKELGGFEGKIITSLSNRMNFTVVAKPPKDGMKWG

>Tdom_96733_putative variant iGluR_partial_translated mRNA

RIPLLKLIFLMWVLGSFILNYCYHASLFGFVTKPKHPPPINTMNDLLQSGIPFGGFAVSRRYFSDPSDYVMKNILKKWKDFSSWFVLTEA

>Psic_11602_putative variant iGluR_partial_translated mRNA

PFLHDKLVLLVPKAQQIPKWMCAILPFDYEVWICYLGVYVLLVVFGKVYSRYRRGNTLALFSFLETFRLLFTGMSQPVTAISLRVYLASVMLFNIVLNNAYSGSFTSYLTVPQYYSDINSF

>Psic_ 12275_putative variant iGluR_partial_translated mRNA

MFSIIVTACYTGSIIAFVTLPLYPQLIDSARQVYEGGFKVAMLDGEGWQDYFNNSHDPIATKVLKKVEHLVAIEDGLRNLTKWRNSRYAVFGSRDKLDFILRANFTHGESSKRAFLHIAKQCFVPFHVAIAFPRRAPHSDMLNQVLLRAVQSGLVLKLKREVDWEMRRSTTGRFLQASSIAAKVPTK

>Psic_13734_putative variant iGluR_partial_translated mRNA

IGDMYSANLTSLLACPGRETPIDTLAKLEQAMREDGYQLLVESHSSSYGILENGTGIYKKLWKMMSHQPYSLISS

>Psic_35409_putative variant iGluR_partial_translated mRNA

WGYMREDGNFTGLVGLLQRKEIDIGGIVSLMTTDRMDVADYTADTVTFRPRFIFRQPSLSSMSNIYVMPFSEGVWATYIATVVVLTVVMFKVQWIKGRSSSLEPHSRDWSEIFLSSLGTTCQQGWHKTPTDI

>Psic_35410_putative variant iGluR_partial_translated mRNA

RFSPFISEYFVSNKVSQVTVFSCQGNDEYLQLSQRLVAAGFLVQAHTSPVAGQLAAILAVDYYRLGVVVDMDCPHMAALLQEASQQRLFSRLHWWLVLVDGASGLPLALSSEPPLDSHMTCVREKGGLYHLVEVWCVAGNPVTSSAEVVWRPGAEMPRQAPRDNLSGASLRTGIVAVEDSWNHRSDLRNKHLDTWNKFSYILATHVAQMMNFRMNETEVDSWGYMREDGNFTGLVGLLQRKEIDIGGIVSL

>Psic_39861_putative variant iGluR_partial_translated mRNA

LNHLTDLENKHIDTISKLGYALAVHAVEMMNATMELRHDPSWGYLVNGSWDGIVGYLERDEADMGSTAVLYTEERAKVVHYLIMTTPTKLG

>Psic_40191_putative variant iGluR_partial_translated mRNA

VLFRSEDTFPDEEKCSLTEVDVYQTRKGYIIVPFQSPLKEILTCALMRVGEAGILSYMYLYMLPQKPTCSSVEEMVSVGAQYVVPTFAILSIGMALSLVAIALEHIWHVCSRETEEI*

>Psic_49669_putative variant iGluR_partial_translated mRNA

KFDIQEFLSSGVSHGVVNILVVSPGIRRNKEPKLVLRTHDLHSGGTIRLLTSWRRGGLTRDVNLFPDKMRRGFNGQSFIVAASDQPHYVIH

>Psic_50572_putative variant iGluR_partial_translated mRNA

RISVLFINKGTDLNSRWITWRLVFFMMLVLTVLLNNYYNACVVSSLLMKAPFNIKTLRDLIDSKVQFGAEKNHYINKFFELDSSPLVKELYQKKMMRPNNQKPIYSPQEEGVRKVLREEFAFHLETINIYGLIEDTFPDVEKCSL

>Psic_51909_putative variant iGluR_partial_translated mRNA

SRGSTGELRVTELYSTGQGAPVLETAAACWSATTGLRYLLERYKYLRRGDMQGQSVRAAVVLNSALKNPEVDLLKAENRFADAMAVYNYHIFLILQKMFNFTLDMVVTSKFGVVVEGGTYDGVVQMLHEGRVDTAISTFVFVHDRMKYIDYR

>Psic_58358_putative variant iGluR_partial_translated mRNA

RMKLAVHDMVYNRYFFERADEPIRRAIYQKKIAPPGVPPRYMKLTDGVERIRKGMFAFHFEVGTGYKLVLDTFDEDEKCGLTRIPYIQVVDAYQVIQKGSVYKEIMAIAYNKLIERGFQKRNWNRY

>Psic_61434_putative variant iGluR_partial_translated mRNA

IPSRLIFFCLDLLSIFFFSSYSAIVVSLLQSPSSSINNLEELVKSPMKIALYDIIYNQNINNYMNISASPVAQRLFRERIHSRPHYEVYLSQEVGMELVRQGQFAFHVEIGAYKFIADTWQEAEKCDLVELYMLPPCTTSLIPVQKGSPY

>Psic_64649_putative variant iGluR_partial_translated mRNA

WTGVVGEVVRGNALIAAAGMLLYRERQEVMDYAVMTTPTKLGFVFRQPPLSSVSNVFTMPFSRTVWLCSAALLVLCGLLLLGALHWERTAADRGYRLAAGVRERVGWTDVILLSVGALCQQGSPVESRGTPGRIVSLQLFIFVMFVYTSYSA

>Psic_69026_putative variant iGluR_partial_translated mRNA

GRLRWLREVGLLERERKRWLVGKPSCASSGAVFVSVGLQEFGPALGALLYGYAFSIAIFVMELLWRRARCLALAPLCITYAR*

>Psic_72009_putative variant iGluR_partial_translated mRNA

KGSPYRKMLTLKLRRVRGVGLLAREKVRWGTNRPRCQDRGAVFTSAGLQDVQPALLVLAFGVLLSASILVLECAHRYLSRI

>Psic_74229_putative variant iGluR_partial_translated mRNA

NLTFHGVVFEVMSALARNLNFSYHVKILSSPSKGQPSNTSSKHVDEFGGETPDISAVSIPWTSLADAVQQKRAFLAAVALPATDRRRSLLNFTTHLSIQPY

>Psic_79628_putative variant iGluR_partial_translated mRNA

PPGWVLTPRSLGSAPPQQFTRQGVFLDHDCASGRRLLQKSSENGLLNGTYHWLVWSEVEDPAARHAVLRRGLRLDSEVAWASRGSAGELRVT

>Psic_83061_putative variant iGluR_partial_translated mRNA

DPLVHELYVKKMLGQHRTKPEFLSREEGIRRVLHERFAFHSEPNNMYPIIETMFPDQKKCSLPEIQIIPIEVCYLPV

>Psic_83400_putative variant iGluR_partial_translated mRNA

GRPRWEGYCVDLLQELADGLRFDYELVEPRGGGLGVRRPDGSWDGVVGDMVAGVSTHQQIASSLLPHLPELFYLHQG
