# Supplementary material for: Evolution of insect olfactory receptors
Source: eLife. 2014 Mar 26;3:e02115. doi: 10.7554/eLife.02115 (PMC3966513; doi:10.7554/eLife.02115)
Supplement: Figure 8—source data 2. — DOI: http://dx.doi.org/10.7554/eLife.02115.022 [file elife02115s009.docx]

>LsigIR1_partial mRNA

TTGAAAAAATCAAAACTAAATTCTTTCTCGAAAGTCGAGTGCCATTTTCTGAAGATATCGTCATTGTGAACGAATGGTATAGTAACACATTGTCCTTTAGAGAACCATTATTATTTCTTGATAAAATACGAAACTTCAATGGTGAGACTTTACGAGTTGAAACTTTTAGCCAACCACCATCAACAATAATATCTGCCAATGAGTATAATCAATTAAAAACATACAAAGGTGTTGAACCAGGTGTTATAATGTTGTTAAGCAAAGCAATGAATTTTTCTTACCAATTTCAAGATCCCTCTGACAACGCAAAATGGGATACCATAAAACGGGATGTTGAGATTGGTAGAGCTGAAATTGGTATGGCTAATCTGTTTATAGATACCAAAAGCTACTTAAGCATGAGCAGTGCGTACGATATAGATTGCATGTCATTTGTAATACCGGTACCACAACGCTCACTTCCGTGGTATTCGATTTTACGGTGTTTCCAGGGTACAATGTGGACAGTAACATCTGTTTTTTTCATAATTTCTGTTTTAAGCACATTCGTTTTAAGTTTGTGCTACAAATGTATATTCGGAATCCCACCATTCAAATTAAACAGTGTTATAAGTATTATCTTTTACCATTGGGGTATCGCCTTTGGATACTCTAACATTAGTTCAGTGAATGTCAGGGTGATACGTGTTTTATTATTTGGCTGGATGATCCTTTCATTTTTGCTGACATCAGCTTTCATGTCCAACTTAATAGCAAACCTCTCAACGCCATCTGCAGTTCAACCAATAAATTCATTGAACGAGCTTGCCCACAGTAATTTAATACCTGTAAGCCCGTTTATCAGTGGTGTATTACACGAAGTGTTTAAAAATTCTTTGGATACAAACTTAAAAATGATATACAAAAGATTGCGTTCAGTTAGTGACGAAACCAAGCGTTTGGAAGGAATATCAAAAGGGAAATACGCAATGATGTTTAGTAAAAATAGTTTGTTATACCAAAAGTCCACAAATTTTGCCAATAAAATAACAAATTTTCAGACACATGTCATGAATGAATGTCTCGTGTCGTTTCACCTGGGATTAGTTTTACAAAGAAGAAGTATGCTGAGACAATCTGTGAATAATCAAATAAATAAAATTATAGCTTCGGGTTTATATTCTAAAATGAAATGGGATGTACTCTTTCCGAACATTTCGTACAATTTTAGACGTCCTATTGAAATTGATACCTCTGCAAAACCATTTTCGTTGAAACACGTCCAATCTGCTTTTGCCTTTCTGATTATTGGTCTATTGCTCTCCCTAATATTTTTCGTTTTAGAAGTTTTCAAAGCAAGAAAAATATATAGACAAACATCAAACTACGAATGATAATAGAGTTATCCAATTGTTTTACAAATGTATTGTCAAATGATGATTTTGCTTCTGTATTGTTGTGACAGTAATGTTGTAATGGATTGTGATGTTAACATAGTTTTGTTTGTTGTGGTAAAAACATTGATGTGTGTAAGTAAAATAATTTGAAAATARRAAAAAAAAAAAAAAAAAAAAAAAAAA

>LsigIR2_partial mRNA

CTCTTTATATGTTCACACTATGATTTTTGATATTGCGTGTACCACTTTCATGGTTCCTCATATTAGGAATGAGTACAAGTGGTATTCAATTTTAACAATTTTTGATATTTGCACATGGGCCGCAATTCTATTTACAGTTGTCATGTGCGCTGTAGTCTTCTATTTATTACTGCGAGCACAAACCAACGACCCCGAAAATAAAACTGCTAGCTATGTATTCAGTTTATTTCATTCTTACAGTAGACTTCTTGGCACATCAGTAATTAAAAAACTGCCTAGCAGACAGGCACGAATGTTTGATACTCTATGGATTTTTATCTCATTGGTATTAGCTTCAGTCTTTGTAAGTAAACTTGTAGCTGTACTCACACATCCTTCTGCGGCACATCAGATTAATACATTAGAAAAACTTCTCAACAGCGGTATACAACCTGCTTGCCAGGCAACTATGTTTTTACGTCATATATTAGCTACACGTCTTGATGTACATGCTAAACAGCTATCCAATAAAATGGTTGGTTTTAATAATGTAAGCCAAGCTTTGAAATTGGTATCAGATGGCCAGTTAGCTTACCTAGATCTATCTTCATATTTGGAGTACGAAATGAACTCTCAGTTTTCAAACAGTGGTAAAATCTCAGTTCATTTGATGAAAACGTGTGCAACTACAAGCATGCTTAGTTTTATATTGAAAAGGAAATCTGTAATGAGGAATACTTTGGACAAAATCATTTTACGAACTGTAAACTCGGGTCTTTATGTAAAATGGCAGACGGATACCAAAAGTGTTGTGCAGCTATCATCAAGTTATTTCACTGACAATAGCGAAACAGCAAACATACTAAGTTTACAACACGTGAAGAGTGTATTTGTAATACTTTTAGTTGGTCTTTTGATAGCAACATTAGCATTAGTTTTAGAAGTAGTTAATAGCAAAAAGTTTAAAAATTAATAT

>LsigIR3_partial mRNA_

TTTCTAACATACTGGCTCGATTTCTAATCTTCTATGAAGGTGAATGTCTATGGTTAAATGATCCCATTTTCAAAAAGGTTCCAAATATTGCTGTGGCAATTACACGTGAGGACATAGGTTTCAGTCAAATTGATATAAAAACACGTGCCATATTTTCAAAAACAGTATATGACAGTGTCATAATGGACATTGAATCGTGGGATGGCAAGAGTTTCACTAAAAATTGTTCAACTTTATATGAAGACAAAACTTCGAATTGGTATGGAAAACACCTTCGAGTCACCACGTTTCACAGCCCTCCTGACGTAATTGTATCTGAAACACATCCAAAGATATATTCAGGTATGGAAATGGAAATATTCAATATTATTGTGAAAACCTTAAACATCTCGTACACCTTTCAAGGTCCACCAGAAGGATGGCTATGGGGTTGGAAGCTAGACAATGGAACTTGGACTGGTACAATGGGTGACTTAGTGTACAATATTACTGATATAGGTCTTGTATCATTCTTTGTTGACAAACTAACGATAGAAGCGTTAGATTGTACTACACCATATGACGGAACGTGCATAACCTTTCTCACACCTGCTCCAAAAGAGATGCTCCACTGGTGGTCAATATATAGGTCATACCATCTTGAAACATGGTGTTTGATCGGGGTAGTTGTACTTATATTTTTAGGTTTTGATTACTCCGTTCATAATCATTTATGGATTCAATCAACACTTAGTTCTTTCGGTTATTCGGTTGTTCATGTTTTTGGCTTTATTACAGGATCGTCTGGAAATTTCAATTCTCAATTCAAGTCAATGAAATGCTTGAATGTATTTTGGTCATTTATATTTACAGTCTTTACAGCTGCTTTTGTGAGTAAATTCATATCTTTAATGACTACACCAATTCCGGAAAGTGCCATCAATTCCTTAGAAGATCTTTTGACCAGTGGTTTACCATACGGCGAGATAGAAAGCGATTATTACACCCAGATTCTACTGGAGTCTACAGATGACGTCACAAAGAAAGTAGCTGGTAATATGTTGGTTGTTCAAAAAGATGTCGATGGAGCTCTACAGGGGGTGTCTGACAGAATTATTTCAGTAATGGAAAGTTCTACTTTGCTTGAATACTACGCGAGCAATCACTTTACAGATGGTGACGGGCATTGTTCTGTGCACTTAATGAAGGAATGTTTAATTTATCTTGGAGTGTCGATTGGCTTGCCTAGAAAGTCACTTTTGAAATATAACGCGGATATTATCATCACACACTTAGTCCACGCTGGGATGGTATCAAAATGGCAATGGGATATCAAATACCCTAAATTACCTTACAAATCAGCTGTGCCAAGAAACAATACTTCACATCCACGTTCCTTGAATATTGGCCATCTCCAGGGAATCTTCATGTTCTGGAGCATTGGGATTATCTTCTCCATTTTTGTATTTATTTTGGAATATCAGTTGAACCTACGAAAGCAAAGGAAAATATCCCAATACATTGTTTCTCATTCTGGCCCTACGGAAGAACCTCAGTGAAAACATTGCCATGAAATACTTAGAAACTGTACTTAGAAAGTTCGAATAGCAAATAGCACATCCTTTACAGCATATTATCACGTTGAATCAGATTTCATTTTATCATATGCAAGTGATGTTTATATATTTCCCAATTTAGAATCTCGGACGATGTTTTGTAAATAAAGACACTGTTGAAACTATATTGGAATAAAGAAACAAAGGTTCCCAACGGTTTTTCAAATACCACGATCAATGTGTTTAGACTGCTGATAGTTAATTAAAGCACTCATATTGAAAATATTTCCTGATGAAGTATATACTACTTTAAGGTATTAATTAGAAATTAAATTTGTAGCTTACACCAGTTGTTGGAATTATTCAACATATCTCATATTCGTTAATTGTTTTGTAAATGCTATTTTCATTGATTTTGATTACAAACAGTTTTTCTTTTTTTGCATAAAATGCACCATCACAGAAAAAATATTACTGTTAAAAATAATTTTTTGATGATGACAAATTGATAGTTTTGAAGAATAACCACTATGTGCTTAGAAAACGCTGTTCACATTTTTGTAAACACATAATATTTTTTTTTAGGTTTACAAAGGAGGTTTTAATGTGCATTATTTAATGATTTGTTGATATCMCCCMCGCAAAATGTAGTTAAATTGTAGAAACATGTGGCMCATGACAATAAAATTAGATTTATCAAAAAAAAAAAAAAAAAAAAAAAAAA

>LsigIR4_partial mRNA

TGCTTTCGAAGCTCATTTTCACTGCTCAAAATTTCCTCTCTTACAAGCTTTTGTAAAACACCAGAAGAATGCAAATTATTAATCCTTCCGTTTACTGTGTTTACGAAAATTGATTTCAATGGCATTATATAAGTGTAAGGCATTGTATAAATACAACCGTCCATGAAATGAATTCTTTCCCAAATATTCACATTACTTTTCGAAAACAAAACAAAATAAACGAAATCATAATAGGACATAATCACTGCAGTTTTGCCTTCTAATAGCAAATGTATTACATCCTTTGGATCAATGTAATTAAGTTTACTTGCAATGGATTTTACGTACTTATCTGAAGATACATTAAAAATTTGAAAAGGCAATTGTAAGTAACTAGAATAGTTTAGATTACTTTCTGAAAGCTCTTTCAAAGAGTTTATTGGCAATTCTCCAGTTGGAGTTGTGAGAATTGATTTCAGATTTGCTAAGAATCCTGTCGTGAGCAGAAGGCAGAATACTTGAAAACAGAAAATAAAAATACGTGTAGGTTTTCTAAATATAGTAAGGTCCGAATAGTTTGCTAATAATATACGTATACATTGAAACAAACTATCTCCAAAAGATATTATTTTGATAGTGGACGATTTGAATATTGTGTTCCAATTAGAAATGAAATAGATAAACAAGGAAGTGATTAAAAAAACCAAACACGAAACTAACCATAAAAACAATGAAAATGATGTGAACAGCGAATACCAGTAGAATTTACGCTGCGGAACAGGTGTCACAAACACATAGCACTCAAACTTTGAAGGATTGGTAAGCCTGAAAGCATAA

>LsigIR5_partial mRNA

GTGCAAAGAAATTGATACTTAAAACACGTAACGAGCTCTTCACGAGTTCACTACTGAACCCCAAAATTGCAGTTGTTCATGAATGGAGCGAACATAGTTCTCTCAGCCACAAGAACACTTTGTTTTTAGAAAAAGCACTAAATTTGGATGGCCAACACCTAAATTTAGTAGATGTCATACATCGAGATAAGGCAGACCATAAAATATGGACAATACTGGCCAAAAACATGAACTTTACATTCACAGTTAAACCCCCAACGCACTCGAAAGGACATAGTGGTGCTGTGCTGGATCTGATTCTAGGTATAAGTGAAGCTTACGTATCATCAGTTATGAGTGAACCAGCATTATATTTTATCATTCAAAATACTTTACCCAATTCAAAAAGGTGTCAAACATTCGTAACTCCGGTACCTAAACGTACAATCCACTGGTACTCAATATTTATCACGTTTGATATGTATTCGTGGGTTGCAACCGGTTTGTGTTTAATCTTTACAACTATATCTGTATGTATTATCTCTAAACTTTCTCCCAAACAACCAAACAGCACTTTAGCACATATTATCAAGTTTGTTAGAGACATATTGTTCACCTGTGTTGGCATACTTCTGCAGAATGGCTTTAGTTTTACAAAATTAACTAGACCTGCTCGGGTTGTACTCTTAAGTTGGGTTATATTAAGCATTCTGCTAACAACAGCGTTTGTGGCCAATCTCAAAGCCCTACTCACAACCTCAACAGGAGGATCACCAATCAACTCCTTTGAAGCTCTCTCTAAAAGCAAGTTGAAAATCTTGACTTACAGCGATTACTTCATTAACAACCTCAACGCATCATCAGATCCCAATATAAAAGACATTGCAAAGAAAATGACTAAAATAAACAAAGTTGAAATGATACCTCAGCTACTTTTCGAGGGTAACACAGCACTGCTGACTTCTTTGAATGAAATAGTTGAGCTTTCTAACTTCTACAATTCTTCAATGGATATTCTAGGTGCTGTGC

>LsigIR6_partial mRNA

AGACATACCTTATCAGTAATTTCATTTTTCAATACATCTTACGAAAAAGGATTCATCAATAATAATGCTATGTTTCTTGTTGTAACCGACAGTAGCAACTGCCTTCTAGACAGAATTTTTCTTAAAGTGCCAAATGTTGTTGTTGCTGGGCGTAATGCCGAATCAATTTCTTTGAAACTACAAACACGTGATTTATTTCCTTCTAGTTCTTCAGTGAACAGCAGTATATTAACTGTAAAACATTGGAATGGTACCATATTTACAGAAGACAACAAAGTTACGTTTGCAGAGAAAGGTAGCAACTTCAACAATCACACAATAAATATAGTAGAGGCCTCCAACTTCAACAGAAAGTTAGATAAAAAAATATGGTCCATTGTTTCACAAAGCATGAACTTTACAATACAGTACAAATATCCAAAATATGGGGACACAATCATAGCTGCTATGCACGATTTGATTTTTGGTATCAGTGACGGAACTGCAACAACAGCTTTTATTAATACACAAGCCTTCTATCTCATAGACATTTCTATACCTATGTCGATAAAATGCTATACATTTGTAACACCTTTTCCTGAACGTGATTTACATTGGCATTCCTTCCTTAAATCATTTGATATGTACTCATGGATTGCAACACTTACAAGTATCATGCTAGCTTCAATTGTAATCTATTTAATTGTCATATTAATGCAGCGTCTGCACGGTACCCACCGAAGAATATCATTTGCTGATAGTTTCTTTGGCATCACAAGCTTAATTCTACAAAATGATTCAAATTTCAAGCCTACACAAAGTTCCACTCGTACTTTCATCATAAATTGGGCTTTCTGTTCTTTGTTAATTACAACTGCATATCAAGCCAATTTGAAAGCTGTTCTGACCACACCTACTGGCCAACTACCAATAAACTCTCCCAAGGATATTGCACGAAGTGGACTGAACTGTGTAGCTTATCAACAAATATTTAGAGAACTTTTAAACCACTCTTCCAACAAATATTTAAATGTCATTGGCCAAAATCTTCAAATAAAGAACAGAAGTGAATCTTTAAAATTATTGTTTGAAGGTCACGCTGCTTTGATATCTACTGCTCAAGATATGCCAGGTCAAGTAGAGGCCCTGGCCTCAGTTCATTTTATGGATATGTGCCTCTATTCCCCCGTTTTTCACTTTCTGTTACCCCTGAGATCAATATTGAAGAATGTGGTAAATAACAAACTAAGGCATCTATTAGACTCAGGAATCATTAGAAAATTGGAATGGGATTATTTAGGTTTTGATAAAGTTCCGTATGGAACTATTTTCCAAAGTGGCGAGATAAAATCACCTGAACCATACAAACTGAAAAATGTTTTAGGCGTGTTTTTTATCTGGGCCTTTGGAATTTCAGTAGCAGCTATTGCATTTGCTGTAGAGATAATTTATGTAACTACAAAGTCCCAAGTTAATGCTCTAATAATCCAAAGAAATAAAAAATAAATACATTTAAAAACATAATTTCAAATAAACAAATATGATATCAAGTAAGCTCTTTTCCAAAACTTTGG

>LsigIR7_partial mRNA_

TTCGAATGTGGGATATATTCAGATGTTTATAAAATTGGAGAATGGAATGGAACACAATTTAAACCAAGTTTAAGTCAGTTGTTTAGTCATAAAATTTGTAATCTTCGTGGGCAGGAGCTAAAAGTGTGTGTGAGTAATTGGCCACCGTTTTTAGACGTAAAAAGAGATGAATTTGGTAGACGGACGTACGATGGATTGAACGTTCAGTTATTGGAATATTTGTCTAACAAATTAAACTTTACTTATTCTTACAAACTTCCGACAAACGGTAACACCTGGGGTGGAATGAAAGAAAATGGGATCGTGCCAGGGATATTAGGTGATATGTATCATGAGTTTAGTGACATAGGAATAGGTGGACTATACCTTGATCATTTGGGGTATAATTTTAGTAATATCATAATATATGAAGGGGATACATTTGTGTTCGTAACACCAACACAGAAAGAGATAGTAAAATGGTATTCCTTTGTTTTACCTTTGACATATGAAGTTTGGATCCTAATATTTCTTTTCATGATTGTTATCATTGTCAGTGTCCTCGTTTTCACAGTTATTTACTCCAAATCTATTTATTCAAATAATTTACCATCCACACATAAACAGGACATAATATTCATAAGTTTGAGTGTTCTAGTAAATAATGTGTTTAAAGTTCAACTAAGAGGAAACAGTACACGAATCGTTATTTTTTGTTGGGTCTACGCGAGTGTGGTATTAAGCACAGGATATATAAGTAAATTGGTGTCAGTTTTAACAATTTCAAAATCACCTCCTCAGTTAGATAATCTTGAGCAGCTTTATTTAAGTAATATCAAATGTGCTACAAGTGATGTAAAGTATTTTGGTGCTATACTAAAAGGTATGAATAACGCTTTTGCTGAGAAGATACGGAGGAACCTAAAGCAAGTGAACCATCTTTCTGAAGGTGTTCAAAAGATTGCAGAGGAGAATATTGCCTATCTAGAAAGTTGTTCTGTATTACGTTACTATTCAAAGCTTCAATACCCGTATAAGAAGTTTCACATCATGGGCCAAAGGTTTGGCTTCTTTTATTTAGGAATTGGTGTGAAATTTAAGTCAGTTTTGTTGTATGATATTGAAGCGTTTATTAGAAAACTAGTAGAGTCTGGAATAATGACTCATTTAAAAAAAAAAAAAAAAAAAAAAAAAAA

>LsigIR8_partial mRNA_

CTGCGCTGTACCATTTCTATATATTATATGAAAGTTTTCCATATAACAAATAGAAAGGATAATTTATTAGAGAAGATGGCTTTTCAAGCAAACTTTTTAACAATTTTTGAAATCATAGCTTTATATACTTACACTGTGTGTGGAAATGTGGAAATCAGTGATGCATACAATGGCAGTTTTAGTCAGAGGTATATGGTAAAGTTTTTATTGGATTTCGCTCCACTGTGGAAATATGATTCGGTATATATAAGTGTTCATATCTCTGGAAAACATTTGGAGAGAGAAATTGGGAACAATATCCGAAAAATGTCGAAGTTATATCTACCATCGATGGGAATTCAACTACCGGTGACAAATTGTACAACGAGAGCCAGCGAAATACTTCAAAAAACTACTATGATTGTTTGGTCTGGCGTCGAGGAATTGGAAACTATTCAAGAGGTTTCAAATTGCATAACGTCGTCAGGCAGATGGATATTTATTTTAAATGGAAACCAAAATGTGAACCAAATATTCCAAAAAGCTAAACTACCATACAAAAGTCAAGTATTTGCAGTACAGCTACATAATGGCAAAAGAGACAAATACCAGATTGACGAAGTATACAGGACTCATTATACAAACTCATTGGCTGTAAACAGAGTTGGTATGTGGAGTCCAGATAATGGAGTCTTTCTTAATTCGAAAAGAGTTCTCAAAAGATATGCAAATCTAAGAGGGGCGCCATTTAAAGTATCCAGTCATGGGTACTACCCTTACGTAGTCAAACAAAACGAAACAATTGAAGGTATTAGTGGAAGAGAAAAGGTAATATGGGATATTTTGCAAGAGGCAATAAACTTCACGTATGTCTATGTGCCACCAACCGATGCTGGTTTTGGGTCACTCAAAGATGATGGACACTGGTCGGGAATTGTTGGAATGCTTGAACGGAGAGAAATAGATATTGGAATGACTATACTAAGCCTGAAGCGATCTAGAATGGAAGCAATCGCCTTCTCGACACCGTTGGCTGCAACACGATATGGCTTTTTAATTAGAGAGCCAACATTTCTGTATTTATGGGACGGTTTCATATATCCGCTGTCAGGAATGTTTTGGGCTTGTTTAGGCATAATTATAATTGTCATCACTTTCATCACATATGCAACGTACATCTTAAGGCAATGCACAAGGATAAGGAATGACACTGAAATTCGTAATTTAGGTTTCACCGGAATTCTGTTTAATATATTGGGAACAGTTTGCTATCAGGGTAAGTACAGAAATGCACAATTAAAATATAAAATGAGTGTTAAATGATGTAATAATTTGTGAATGATGACAATTAGTTTTTGACAATAATGTTACTCGTTTAATGTANNNNNNNNNNNNNNNNNNNNNNNNNNNNNNNNNNNNNNNNNNNNNNNNTTTTTTTTTTTTTTAAACATATTCCAACAATATAGCTATGCTCAAATGAATGTAATTTGTCAATATAAATTTATACATATAAGTTATAGGGTGATCTTAAAATAAAGAGTCACTCATCAAATATATTAGTTTTCAACATCCAGTGATTTTTTTTCTTTTTCGAC

>LsigIR9_partial mRNA_

CATGGGATTACTTTTTTCGAAAAATCAGCCATTTTTGATGTTAACCAAGGTGCTCTTCGTTCCCCGATACGAAGTTCGGGCATCCCAGCTAGTAAATGAATAAAATAAATAAACCACTATCTGCATCGGCATCTACTGGGTGAATTATAGGGGGACTTGTCGTTCAAGTGTGATAGAGAAGTTTTCCGAAACAAACGACTGGTCTTGTCTTTATAAATCGCGAGACTACAAATTTTCGTAGTGCGATTAGTCCTTATTGGTTACCCAGACTACTGTAAAGTCTTTCAAGGGAAGCAAAACTTCCATTTGAAATATGTGTTCGACTAATAGTCTTTTTCCAAACTTGCAGATCGTGTTTCCTATTTCGCCAGCCTTCTCTGTCTTCGGTGTCGAACATCTTCACTCTTCCATTCACGCTGTTCGTGTGGCTGTGCACCATTTCCCTTCTTCTAGTTATTGCCTTTGCTCTGTTCTTATCAGTGAACGTGGAGAAACATTACAGCAATGAGGATCAAGAGGCCGACTATTCATGGAATGAAATTCTCTTAGTGGCTCTCGGTGCCATCTGTCAACAAGGCTCTCCCTGGGAACCGAGGTCCTTCTCTGCACGCATCACGTTCCTCTTCTTGTTCGTGCTGGCAGTGTTCCTCTACAACTCCTACGCGGCCTGCATTGTATCTTTGCTGCAGTCCACATCAACAACAGTTCGCAGTGTCAAAGATCTTATCGACAGTCCTGTCAAACTAGCAATTCATGACACGAACTACACTAGAATGTATCTAGAGACGTCAAACGATCCGGTGGCAAAGAGGATGGTGAAACGTCGCAAACCTCGTTATCTGGATCTGAGTGACGGAATGAATCGAGTACGTCGAGGCATGTTTGCCTTCCACGTGGCAACTATTGACGGTTACACGGAAATGGAGCAACAGTTTACGGATGACGAAAAATGTGGTCTGCTGGAACTACCTCTTTACAGAGCCGAAATGTTGGCCTTGCCCCTTCCAAGAAACTCGCCTTACAAAGAATTCTTCAATCAAGGATTGCTGCGTATAGTGACATCCGGTGTTCGCAGTCGGGAATTCACACGTTGGGTGAGTCAGCGTCCCAATTGCCTTCAAGATGGCGGCGGATATGTGAGCGTGGGTTTCCTGGAATTCAAACCAGCCCTGGCTGCACTTGGCTATGGCATACTTTTCTCATTCTTATTTCTTGTTGGAGAAGTGCTCTTTCAACGCAGACACAAGATTCTACAGTGCAAATTTCTTGAAAGAAGTCCTCCAAGCCAAGGAGAGTTATTCCGTCGTTTGCCTTCACCAAAACAATTCGCACGCTCCTACCAATTACCGTATCCTTAATTATAAATACATTTTATGTGGCTTGCAGGGGCGTGTGCAGACTTCCTGGATCGCAACCTTGTATTTACTGTGCATGTGAAGCTGAGTTTAATTTTTAACAATTAATGTGTAAAATTGTTCATAACATTCACATAAGCTAAGGGTAAAAAAACTACATGATTACATTAAATGTGATTAATATGAACATTGTTATCCTGCTCGCTAAAGCATATAACCTCCTGGGATTTACATCATAAAGCGTTTTGTAGAACTGTATTTAGGTTTYTGCACTTTTTGTTAGAGTACAAATAAACAAAATAAAATTAATTCATGTATTTCAAAAAAAAAAAAAAAAAAAAAAAAAAAA

>LsigIR10_partial mRNA_

ACATGGGGATCTCTGCTCACATTTGCTGAAGACACAAATGTAGCTGTAAGAAATGGGCAACCCCAAGATGACTTTGCGAAATGGAGGTTTCCACCGGACCTTTTGAAGTCCATTCACACCTTCGCTTTTATCAACAAATTTTGCATACTCTTACCTCAGAGATATTTCGAAGATTCGAGATTAATGTTGCATCTGAAAGACGTGGCACGTTTGCTTTCAAGATGGAAAATTCCTTTTACATTCCAAAGTGTGTCCAATGGAACTGCTGACAAAATAAATTACCACACGACACCAAATTTAGGATTTTTGGTATATGAGGAAGAGAGCCATCTTTCCCAGAACTTAGCAACGGCTATCGAATCGACAACTACCTCACCAAAGTGGATTTTCACTATGGCAGAAGAAACGGAAGTTAGATCTTATTTCGATGGTGTTTATATTACCCTGCGAAGTAACGTTCAAGTCTTTCAAAAACAAGATGGAGGACCAGGATACAACATCTTCTCTGTATACAGGGTCGATAAATCTCATCAATTACGAATCATTGAATTAGGGGAGTGGATTTCTTCTAATGAATTCAATTGTGACCTGCTGCACATATATGAATTGAGACACAATTTGGAATTAGTGGACTATAAAGGACTTCAGAGAACAATTTCAGACATGCCAGACGAAATACGAAAGCATTACATATCAAACTCAGTAGGATTGGCTCTGATGCTTATGGAAACATTTAATATCACCATTTATTCTAGCGCACAGCTACAGTTTTATGAAACAATGGGTAAAAGAGACAGTAATGGAAAATGGGATGGTGCTTATGGGCTGATGCAATCTGGTGAATCTGACTTCATGACAACTCCAATGCCTCTAACCAAAGAAGTTTATGAGTTTGCAGCTCCAGCTTGTCCACAATTGTATTTATCATATCGACTGTACATCAAGAAGCCAAGTACGTCGTTCCACGATTGGATATCAATAGTCAAGCCTTTGAGCAGAAATCTTTGGACAGCCTTCTTGGCAAATTCCTTCCTTGTTGCTGTATGCCTTGCAGTGGTAAAGATGATTTTCCAGAAGATTCACATTGAAGACACTGAGGGCAGCACCATGTCAACTATTACGTACTCTATTCTGTCAATTCTTGCAGGGTGGACTGGACAAGGAGTGGAATCTCTTTCTAAAAGTGCAACTATTCGTATACTAGTTTTCTCCAATTTAATTGCTGCTGTGGTTATTTACATTGCTTATTCTTCGGAGCTACTTTCCAATCTCTCAGTTCTGACTCTGCAGCTTCCATTTTATTCACTGGAGGAATTGGTTGAGCTGAGTAAAAGACATGGGAGACGCACATT

>LsigIR11_partial mRNA

AAGAAGAAGATGGAGTCGACTCCATTCTAAACTTGCCGACAGCATGGAAGCTCGTACAGTCGTTGTTGAAAAGTGTGTTTTAAATTTGCTCTTACACCAGTTTTCCATGCTTGGAATCAGCAAAATATACAGGCCATCGTATCAGCTGATAGCTTTGGTTTGCCGTTAGGAAATGGCAGCTACGATGGTGGTATGGGGATGCTGGAACGTGGAGAAGGTGACATGATATTACAAGCTGTTTTATATAATGGTGATTTTGTTGGCAGCATTGATTACACTTCAACAGTTTTCAAGGGGTGGTATCAAATGTATATCCATACACCCTCATCAGAGTTTGGCTGGGCAACGGGATTTGTGAATGAATTTTCAGTTGGTCTTTGGAGAAGCTACGGACTACTAGCTTTGGTACTTACTGTTTCATTGGCAGCAGCGACGAAGATTGGTCGTGCTTTGAAAGTAGAATCTTCCATCTCATCCCAAAATATGTTGGAGAGTTTAACATGCGTTAGTGGTGCAACGTGTAATCAGGGACCTGATATTCAACTTCAAGCTTTGGGACCCAGAATAATATTGTTTTCCACTTTTCTGGCATTCTCGGTTCTATACCTGGCTTATTCATCGGAGCTAATGTCGACATTAACATCAAAGACCATCGACATTCCTTTTCACAACTTGGATGATCTCGTCAATTTAAAGAAAGGACGGTTTGTTTTGGTAATGGAAAATGACTCTTTACCTCACAACAAGTACATGAAAGATAACTCAAACGTATTTAAAAGAGCACGAGAAAATCTCGAGACAAAAATTGTGGAAGATCTTACAGTGACTGAGGAAACTTGTAGTTCTAAACGGAATGTGTTAATGTATGTCTTTGACTTAAGACAACAAGCAGCGATAATTGGATGTGATCTCGACAAATTACCCAAGAAATATTTTGAAAACCCTGCCGTTTTCCCTGTCAGAAAGAACATGTTTTGGCTACCAGTTATGAACAGAATGTCATCCAACATTTCCCAGGAA

>LsigIR25a_ mRNA

GCAGTTAATTATGGGTGTACTGCACTACTCATAACACTGTAACACACGTAGGTCTGTTTTGATAAAACAACCAGTCCACCAATGAATGTAAAAGACATCTACGTTTCGTTTGGTCTGTCTACACGTTTGCTTTCGATTGATAATGGCGATGACCTTACTCTGAGGTAGTCGCTAACCAGCTGTTTAAGGGTAATCCGAGTAATGCCGGCAATCGTAGCTATCGAACAACAAAAATGTATTGATAGTTATCCAGAATTATTCAAGTGGAACAACCAATCAATATATTTCTTTCACAAAAGTTCTCTTCAGATCATAACAACATCTTGCAATTTCTGATAGGACCTACAATAATTTGTACCTAGTATAAAATGGAAAAAATAACTACAATGTTGGTTCAATTTTCTCTTTAAATTCAGAAGGATTAAAAAATAAATTCTTACAAGACAAGGTTAAATGCACATGACAATACTGATTGGCAAACATCTTTCAAACAAACCTGAGAAATCACTTGGAACAGACAAACGCTTTCACTAAAACCGATTAAATGAAGCCATACATTTAAATAAGATATAACGCTTCGTAAATACACTTGATAATGTATATCGTTGAATATCACATTGCCCACTATACATGTAACATCTGTGCGTCAACACACAAAACTCGCACACAAATTCATTTGTGTAGAATTGGGCTTCACTTTGTGGAAAAATATCTCTGGTACTTAGTATTTGTACAATAAACCTCTCATTGACGATAACAAATTGTGTCCTAAAACGGATTTCCTGGAAAGCGTACACCTACGCCCCTGAATTCATCTTGAAGGGTGGCAGGAGGAGGCCGTGGATTGAAAGCCGTCGCGTCCGCTGCCCCTCCGAAGTTATCTTTGTTTGTTGGGAACTCGCGAACATTGATCTTTTGCACGTCTACGACTTTTGAACCTCCTTTCTTGAAGCGATACCACCAGTATTCAAAAGCCAATGTGATAATGGCCAAGCCGATTCCAACAAAAATGACAATGAAGACTCCGCCAATGTTTTCAATGCTTATGCCGTCTGATTGGTTGTCATCATCGGTGCATTCAGACAAATTTTTGGGTCTTCTCCACCAGAATTCTTTAAACTTTTCCAGTTGTCTCTTGTTCAAAAGCTGCAGAATAGCATTGTTGAACTGATCTTTGAGAGGAGACCCCTGTTGGACGGCAACGGCGTACGGTTTTCTTGAGAACTCCTCTCCTACCATCTGCAGGTCACAGTTTGTGAGAATTTGATACCGAATGTCGGTGGCATCACCCAGAAATGCAAATCCTTCGCTGGATGACTTTGACTTTCTCACTCGTGCCAATGCTTCATCAAAGTTTGCCGGGAGTCCAGATTCCTTCATCGCCTGCCACATTTTGGTGTACTTGTCACTCACTGGGTAGTCCCACACAGCCAGCTTGGCCCTCTCAACTTCACTCAGGCTGTCGTTCAAACTCATGTCCTTCCAGATCTCGTAAAATTTGTTCTCGATGTACGCCATTCGCTCGAAGTAGGTGGCTGTCGCCGACCCATTGACAGGGGCATACTGAATCTTGTACTGCTTGGAGAGGTCATCCAGAGATTCAACAGGTGTGTCCAAACGGGATACGGTCAAGAAAGCAGCCAAGTTAGCAGTGTAGGATGCAATGATGATGAATCCAAACAACCACCAGGTAGCAGCTACCAGTCTTCCAGAGAGGTTTTTCGGGGCTTCTCCACCGCCCTGCGGTGTGAGTGACGTCATGCAGAACCAAAGACATTCTTTGAGGTTGAATTCTCTCTTCTCTTCATCATCTTTGTACTTTTCTCGGTTGTTCTGGTAGCTGTAAGGACTCCACCGATCGAAAACCCACATTAGGAAACTGGTGAAGAAGTAAGCAGCTAGAATGCAAAGCCACACGTCGTTTTCGAGCACAGTGAGAAACTTGAACAGAGAAGTTGGTACTTTGGGCTTCAACATGAGAATGGTGATCCCGACGAGATCGTAGTATGGCACCGTGAAATCAATGACGCTTTCTCTTTCAGCCATAACGGAGAGAGAGGATAAGGCCACGTCTGCTCTCTTTTCCATGAGTTCCTTCACCATTCCGTTCCACTGATTCTGATCGTTCATAGCTCCAAACTTCTTATCCGGTGCCTCGTAAATTTCGTAATCAAATTGGAGCAGTTCCTTGATATCGTTCAACAAATCGATGCAGTAGCCAACAAATTTCTTATCCTCTCCCTCTCCAATTGCCATTACGAATGGTGGTTGCACAACTGTTACCACACGATATGTGGTTTTTGAAGCGAGCTCCGTGAGATCTTGTCCATCGGCATATGTCAGAGTTCCAGGAATGCCCACTTGCCAGGTTCCAGCTACCTTGCCTCCTTTTGGCTCACCCTTGATGATATCCAGGCGTTCCAGTTTCACTTGGAATTCCATGTAGCTTTGCCCATTTTCGTCAATCTTCAATTTCCCGTAAGAGTTGCTAGGGCGCTGAATCTGTTTGAGCGCATTTCGTAGGTCGAGACCCGTTCTGGTAATGGCGGTATCAGAGTTGTGGTCATCACATTTCACAAACTTCATATTTTCTGCTGCTGGCCATTCCTCCCTGTTCAACATATTTCGGACCGCCATGAACGCTGTAGTTATGACGTCAAAGTAGAAGGCGACTTCTATATCCGGTTTGGGTCCACCAAGATTGTAATTCTGTTTTAACAGCTGTAGATTGTTCACGCTTCCAGGATCAGGTTCGGGTCTTCCAATGACGAGGCTTGCATTGGCGCACTTACACTGTACAGTGTCTCTTGAGTCCTGAGTGAAGATGTACCAGGCATGCTTTTTTCCAAAATACTTCATGTCGTCCGCCGAATCCAAAACTTTTTGTGCAGACGCCGCATTCCCCATCACAAAATAGTTGACAACGTCGACGCGTCGCAGATTCCAGAGTTGAGCCTTCACATCGTCATCGATTGTCGAGATGACGTGTCGCGTTGGAAGGTTTTGAAGCAAAGATTTGTATTTGTGGTCCATCACGTATGACTCATCAAAGAGAACAGCAGCATTTGTTAAATTCAGCGTGTCTGCTAACGAACGGACAATCATTGGAATGACGTCAGCAGGAGGCATAATCTGCACCAGGTACTTGTTGGCTTCCTGATCAAGGTTGCGCCATTGTCTAATGTCGTTTTCTTGCCCAAATGAAGTGGAAACTGTCGGAAGACCCAAAGTTTTTGTGATGTACTTAATGGCTTCCGAAGTAAGGCCGGCAGTTGTTGAATCAAACACCAGATGGAGTGGTTGGTCTCCATCTATTAATGCATCATATTCTTTACAGAATTTATCGGCAAGGATAGTTGTGTTGGCACTGTCGGCATAGACGCGCGCTTCCTTTCCGACAGTGACTTCTAATTTAGGATCTGATCGGAATTGGCTCAACACTGCGTCGAACGCTTTTTCTGCATTGGTGTCTTTTTTTTCACCGACAAACAAGACATTAATGGTTTGAGCACTGTGAATTGGATGGATCACAAATAGCAACAAGGAAATACACACCAATAGCATCTGTTGTAGAAGAGTAAGTCTGCCTCTAACAATCATTTTGAGCTGTCGTTTAAAAAAATCCTGTAGTGATTTACGGTTAATTTTCACTCCTCATTATATATTATGTAATCTGTTGTGTCTTGCCCAAAAATATTAAGCTAGATATAAACTCAAAGCATTAAAAAAATATTCGAACAGTAAAGTCTTGATTCGGCCTGCCGACTGACT

>LsigIR8a_partial mRNA

AAGACTGCAGGGCGAGTCCCTTCTTCACCATTTACAATGAAGCTCCATTTTATGTGCCTTTTCTGTATCTTCCTTCATTTATCACAAGGTCAAGAAGAAGAAGAACCTGCTGCTCCTGCTGCAGACGCAAAAGAAATAAAAATTTTGGTGGTTCTTGAAGAACAACAGCAAGTGATAGCCGACCATTTAACTTCTGGCTTTCAGAAAGCTGAAAATACCGTAAACATGAACGGGATGAAAGTAACAATCAAACCAATTGCCGTAAGTCGTGAAGAAGAAGAAACTGGATTTAGTGAAGTCTGTAAAGACCTTACAACAAACTACAACCTTATTCTTGACTTTACGTGGGGAGGCTGGTGGAGAGTTCGGAATATGGCAGAAGGCTCTGGAATGCCATACCTTCGGGTCGACTCTACAATTCGACCGTTCGTTCAAGCAGCTGATGACTGGATAAGAACACGTAATGGAACAGATGCTGCCCTTATATTTCAGAATGAAGGTGAGTTGGACCAAGCACTCTACTATCTGATCGGAAACTCCATTGTCAGAGTAATAGTCCTTGATCGGCTACCGGGAAACACAACTGACAGCTTGAAAACTATGAGGCCATCACCATCTTATTTCGTCATGTACGCCAAAACTCAAGAAATGCAAAAATTGTTTTCGATTGCTCTAAAAGGAGACATTGTTACCCGCGATTCTCGATGGACACTTGTGTTTCTCGATTTTGAGTACGAAGGTTTCGCCACATCTGATTTACCTGTCAACGTGTTCTTCATTAAGATGCAGAAGAAGGTTTGCTGTCAGCTCTTGGACCAGGGCACTTCATGCTCGTGCCCTGCAGATATGGAGATCGTGCCAAATTTTCTGCTACGTGCCACCAGTGTACTCACATCGACATTGGTTACTCTGAAAGGTGAAATGCCTGACCCTGACCCAATTGACTGCAAATCAGTGAATCCGGAAGCGGCGAAAAATGACACGAGGAAAAGATTCCACGAAATACTCGACATGGAAGGCAAGAAGAATCCTGCAGTTGGATTTGACCCAGAGATATTGCTGTTGTATTATCAGTCTGAAATGGACATCACATCTTACACGAAAGTGAACAAAACGGAAGTAGCGACGTGGACAACAGAAGACCGCTTCGTTCTCAGACCTGGATATAATTTAACAGCTGCACGTCGCTTTTTCAGAGTCGGCATTGTCGAGGCCATGCCTTGGACTTTTAAGAAGAAAGATGAGAACGGAAAAACCATGTTTGATGAGAAAACTGGGATGTTTGTCTACGATGGATACTGTATCGAGTTGCTGGAGCGGCTCTCTCAAACAATGGGCTTCGATTACGAAATTGTATTACCCAGAGACGGAGAATATGGCGAGAAAGGGTCGAAAGGATGGACAGGGGTCGTTGGAGATCTGGCTCGAGGAGAAACCGACATTGTGATTGCAGCCCTGACAATGACATCTGAACGTGAAGAAGTAGTCGACTTTGTCGCTCCTTATTTTGATCAGTCTGGAATTTCCATAGTTTTACGTAAGCCTGTGCGCAAAACGTCTCTCTTCAAATTCATGACTGTGCTGCGACTGGAGGTATGGCTGAGTATTGTTGGTGCTCTCACTGTGACTGGGCTTATGATCTGGGTTCTTGATCGCTTCTCTCCATACAGTGCTCAGAACAACAAGGAACTCTACCCTTATCCATGCAGAATGTTTACCTTGAAAGAAAGTTTCTGGTTTGCTCTGACTTCATTCACGCCTCAGGGTGGTGGTGAAGCGCCAAAGGCTTTATCTGGACGAACATTGGTTGCAGCCTATTGGTTGTTTGTGGTATTGATGTTGGCTACTTTCACTGCCAACTTGGCTGCTTTCCTCACAGTGGAGAGAATGCAGACACCAGTTGCGTCGTTGGATGAACTTTCCCGTCAATCAAAAATAAAGTACACTGTCGTGAAAGCATCAACCATTTACCAGTATTTCGAAAACATGGACCATGCTGAGAAGGAACTCTACAAGGTTTGGAAGGAGATCACCTTGAACAGCACCAGCGACCAGACCAAGTTTCGTGTCTGGGACTATCCGATCAAGGAACAATATGGACACATCAAGAGTGCAATTGAGAACACAGGAATGCTTGACAATGCGACTGAAGGATTCACAAAGGTTTTGGCCAATGAAAAGGGAGAGTTTGCTTTCATTCACGACGCTGCTGAAATTCGCTACGAGGTTTACCGCAACTGCAATTTCTCGGAAGTGGGCGAACCATTTGCTGAACAACCATACGCTATAGCTGTGCAACAAGGAAGCCATCTGCAGGAAGAAATCAGCAAACAGATCCTGGACTTGCAAAAAGATCGCTACTTTGAAGGCTTGTCGGGCAAATATTGGAATTCATCACGTCGTGGGCTGTGCCCCAATTTGGATGACAGTGAGGGTATTACACTGGAAAGTTTGGGTGGCGTGTTCATTGCAACGTTATTTGGTCTGGTGTTAGCGATGTTCACTCTTGCGGGAGAAATCTTCTTTCACAAGCGCAAAATTAAACATCAAGTGCACAACATCACCAAATCAAATGCACTCACTCTCGCAAGTGACTTCAGAGGTACAGAGAAGTCAGTACCTCGTGTGTCTTACATCTCTGTGTTCCCGCGTAGTTAAGTCACTTGTGCAACATTCACAAGTAGTGTGTTTTGGATGTACAAAAGACTTGGTAGCACAGGAGGATTTTTTCAACTGTAAATAAGCATTACATTAAGATTGTGTATAAAAAGTGGCAGTTAAAATTCAAATGCAAAAAAAAAAAGGTTTTGCAAGGTCCTGCCCGTACACAGTCCTCGAGCATATCTCCTCCCAAAATATATAATTCTATATATATATATTTTGTATAAATTCAAACTACTTGCATCAGGAAATTTTACGATTTATTATTTTCACCCCTGTTTAGTATGTATTAAAGTTTCCATTCTCAACGTTCAAATTTGAGAAAGATATTTCATAAACAAATATTAAGCAAGTTTTTTAGTACTTATTAATATATTATATTGGATATATCATGTATACGAGTAATTAGAGTCTTAAATATGTTCATTATACAGCGTGTAACAGAAAGATCGGGACACTTAAACATATCACAAACCACGAGGATTTCAAAAAATGACATGATNNNNNNNNNNNNNNNNNNNNNNNNNNNNNNNNNNNNNNNNNNNNNNNNNNNNNNNNNNNNNNNNNNNNNNNTGGTCGGGATCCGATGTGGGCCCTATTGGACCCGAGTAATGTTTTTCCAATTCAAACCCCAATTTATTAAGTCACATTCTTGTTTTGCATCAAAAAATGAAGTTTATTTGATGATTCCATGAGGCCTAAATTGCTATTCGAAGATCAAGGTTGAGGGAGTTCAGAGAAAAACGTTGATCTTGTGCAATACTATAGACTAAACATTAGTTTTCAGAGAGAAAGATTAACTTTTATATTGGTAAGTAGGAATGGCAATGATTGTTTAACATTGGGCCCAAATCCCATGTTATGCGCAATAAATCCCAATAAGGTCCGAATCAATATTTTTACACGAAATCTCTCATTTGAATGATTTTTCATTCTTAATGACAATGTGCATTAAATTGTGGACCCAAAGTCCGAATGGGTCCCAATTAGACATATTTCCATTTGTTTTCATCCGATTGAGCCCAAAATGTTATTCACGTTATTCTAAATAATCAAAAGTGTTTAAAATTAACAGTTTTGCCCAAAACATTGATTTAAGCCACATTGGACCTCATTAGGCCTAAACTGGAACTTTGGCCCAAAAATTACATACAAGCTCGTAATTTTTAGTAAAATAAGGGTTTTCAGTAAAAAAAAAATTGACGTGCTTGATTGAGCCCCACTCGCGCTTTGGGCCCAAATGTGAATATGGATCCAAATGAAGCTTTGCACCCAAAATGTAATGTGCATTGTTATGGGGAATGAAGAGTCATTCAAATGAAAGGTTTCATTTAAATATTGATTCGGGCCCAAAGTTAACAAAAGCATTTCCATTCACATTTAAGATCATAATTATTTCTGGAAACTGTGATGTGTT

>LsigIR76b_ mRNA

TGGGGAGTAATTCTCCATTTCGTGTCGAGATTACATCGTCGGCGTGCGAGTCCACGTCGCTCTACCAGTTCAATCGACACTCGACAGTGATATTGGCAAACCAAGCTACGCCAGTCGCTTGGTGGAGTTTGGACAAGGTTAATAGAAACTTTTGGAAGCGTGAAAACAAATTTCTTCTGTTTCCACTGCTGTTGTCTGTAAAGTGTACGACCATGTTCTACTAATCAGGAGGGGCTGTTTAGATTACAGCTCTTCTCGTATGATGTCATTTCACCAAAAACAGAGAAAATACGTCTGTAATATACCAGGACCGTAAGTAATTGTGCTTCATAAATCATGGCCGGATCACTGAATTTTTTGAAAACGGTCATGATGAGTGTATGCCTGGCTGGAGCTGGCAAGGATGGAACCATTGATCCAGCGACTGTTACTGAAGAATATCTAACAGAACAATGTAATATGAACAGACTCCCAAAAATCGACTTCTGGAATCTGAAACTAGGACCTTCCAAGGAATTGAATGGAAAACATCTTCTAGTTGCGACACTACAGGATCCGCCACTTCATTACACGGAAATGGACGAACAAGGAAAATTGGTTGGTAAAGGAATCGCGTTCGACATGCTGAACCTCCTCCAAGATCACTTCAATTTCACTTATGAGGTCAAAGAAGCATCAGAAAACTACATCGGATCCTACGACGCAAAATTGAAAACCTGGAACGGTATCCTCGGCATGCTGTATCGAAAGGAAATTAACATGTCTGTTGCGTTCACCCCAATTACGGTCACCGCCATGAAGGACATCGACTTCAGTTTCGGGATGGGAGAGATGAAATGGGTCCTGATGCTCAAACGACCGGTGGAATCTGTGGGCGGTGACGGTTTGTTGGCACCATTTCAAACAGGGGTGTGGTATCTCATCCTACTGTCTCTGGTGATAATGGGCCCACTCATCTATGGGATCATCTTGTTCCGAGTGAAGCTGTGTCGAGGTGACGCGAGGTTGACAAGAATTTTCCCCCTCCATCAATGCATTTGGTTTGTCTACGGTGGTCTGGTGAAGCAGGGTTCAACACTCAGCCCTGTCACGGATTCCTCCCGCCTGCTGTTCGCTACCTGGTGGATCTTTATCATGATCCTCACGTCCTTCTACACGGCCAACCTGACTGCTTATCTCACTCTGTCCAAGACAACGTTGGCAATAGAAACCAAGAAAGACCTGACCGGCAGCTCCACCAAATGGTTTGTGAGAAAAGGAAGCGCCATTGAAACAGTGATCACGCACGAGAAAGGTCTCGAAGACTTGGCCAAGATGGTATCCAAAGGGTACGGAAGCTATGCCCGAGCTGATCTGACGGACGTTGAAATTATGGCCATGCTTGAAAAGAGGAGTGTGACATTTATCCAGAGGGCGAACTCCATCGAACGAATGATATTCCGCCACTACTTGAACCAGACCAAGAATGGAGTACCTGAACCCAAGCGATGCACCTTCGTGTACAGTTCGTCCCCTATTCTGAAACTGGAATACGGATTTGGCTACCAAATGGGAACCCCATTAAAGAGACTCTTCGACCCAATGGTGTCTTATCTGGTGGAATCTGGCATCACGAAACATCTACTTCAGCTGGATCTTCCTAGTCGAGAAGTGTGTCCACTGTACCGAGGTTTCCAGGAGCGACCAATGAGAAACGAGGATCTGTTCATGACGTACACGATTGTGGGAAGCGGTTACGGCTTGGGATTCTTGTCATGTTTGGTGGAAATGTTCCTGCTCTACATCTGGTATCCCAAATTTTCCAAAAAAAAAAAAAAAAAAAAAAAAAAAA

>LsigGluR5095_partial mRNA

GAGAACCCGCCTCGTTCCCTATTTTGAGAATGTTCCACTCAATAACTCTTTTAAAGCAACGAAACATGTTTGCAGCATCCTTCGTCAAAGTGTCGTAGGCGTCTTCGGGCCTCAGTCGGAAGAGTTGTCGGGCCCAATTCAATCTGTATGTGACGCAGTGGAGATTCCGCACTTCGAGAACCATTGGCAGATACAAGACACCCGGGAATGCTGTCGATTCAACTTGTACCCGCACTACGAGGCGCTCAGTAGGGCCCTTGTCGATTTAGTAACGGAATGGGAGTGGAAGACGTTTCTCTTGCTGTACGACGACGACGATGGAGTGGTTCGTCTTCAAGGCTTACTCAAATCTCTCGCTAACGATAAACAGCCTTTTATAATGCGACAGCTACCGGAAGGAAATGATTACAGGCATCTTTTACGTGAAGTAATGCACACAGGAGAACACAATATTGTGATTGACTGCTCGGCAGATAAAATATTTAACGTGCTCAAACAGGCGCAACAAGTTGGCATGATGACAGCGCATCACAGCTATTTCTTAACCTCTTTGAACTTGCACACGGTAGACCTGGAAGATTTCCAATATGGCGGCACCAAGATTGTGGGGCTTCGAATAATAGACATCGACGACGAAGATATCATAAGAACTGTTCGCCATTGGAGATATTCCGAGATGAGGAACAGTAGAATCCTTAACTTGTCTGCCGCCAGCTTAAAGACAGAAACAGCCCTCATGTACGATGCAGTTCATATTTTTGCCAAAGCTCTCCACGAACTAGACAACAGTCAGTTTGTTACAATACAACCAATGAGTTGCGAAACTCCTGATCCCTGGCATTACGGATCCAGTCTGATGAACTACATGCGTGTGGGAGAACATCGAGGCCTCACTGGTTTAATTCGTTTCGATCGTGAAGGTTTCCGTTCCGATTTCATGATCGATGTTGTCCAACTGTACGAAAACGGACTCAGGAAAATTGGTACGTGGAATTCCACTGAAGGGGCCAATCTCACCAACATCGAGCATAAAGTTATACAGCTTGGTCCAGACAGTCTAAGGAATAAAACTATGATCGTTAGCACTATTTTGAATGAACCCTATACAATGCTTAAGGAAAGAAGTGAGAAGTTGACCGGAAATGACCGCTATGAAGGATTTGCCATTGATCTCATCCATGAGATTTCGCTAATTCTTGGCTTCAAATACATAATCAAGCTTGCTGCTGATGGCAAACATGGCTCCTATAATGACACCACCAATACATGGAATGGGATGGTCGGTGACCTTAGAAGAGGGGAGGCAGACCTGGCGTTAGCTGATTTAACTATAACTGCACAAAGAGAATCAGCGGTGGAGTTCACTATGCCATTCATGAATCTAGGAATCAGCATATTGTATATGAAACCTCGGAAGCAGCCCCCTAATCTTCTCTCCTTCCTCTCGCCACTCTCCATGGAAGTTTGGGTGTACATGATGGTTGCCTATGTCGGAGTCTCCTGTGTCTTATACAGTTTAGCCAGACTGAGCCCGTACGAGTGGGACAACCCGTATCCTTGTATTGAAGAGCCTGAAGAATTAGAAAATCAGTTTACTTTACTCAACAGCCTGTGGTTTACGATCGGATCGCTTATGCAGCAAGGATCTGATATTGCACCCAAGGCTGTGTCAACGCGAATGGTAGCAGGCATGTGGTGGTTCTTTACTCTGATCATGATCTCCTCTTACACGGCTAACCTGGCTGCCTTTCTCACAGTAGAAAATCTTGAATCGCCCATAAATAGTGTCGAAGAATTAGCAGCACAAACTAAAATCAAGTATGGAGCATATAAAGGCGGTTCGACTGTCAAATTTTTCCAGAATCATCCTCTGGAGTTTTACAGAAAAATGTTTGAAGTGATGAAAGAAGCAAAGCCGTCAGTATTTACATCGACGAACCAAGAAGGAGTGGAGAGGGTTGAAAAGTCGAATGGCCTCTACGCCTTTTTCATGGAGTCTTCTTCGATTGAATTTAATGCCGAAAGAAAATGTGAAATCACTCAAGTCGGTGGTCTGCTCGATTCCAAAGGATACGGAATAGCTTTACCAAAAGGGTCCCAGTACCGTTCTGCTGTCAATGGCGCTCTTCTTAAAGTCCAAGAACAAGGAAAACTCCACATTTTAAAGTCTCGTTGGTGGAAGGAAAAGAGAGGTGGAGGCCAGTGTACTGACGATGCTGGAGGTGGTGGTGCAGCTGCTGAGTTAGGCTTAGCCAACGTTGGTGGTGTGTTTGTGGTGCTGGTTGGCGGAATGTTCACTGCCTGTTTCATTGCCGCCTGTGAACTCTTATGGACAACAAGAAGTGTTGCGGAAGAGGAAGGGACTTCTTTCGGTTCAGAAGTTCTTGCAGAGTTGAAGTTTGTCGTGAGCTGTCACGGTTCAACAAAGCCAGCCCGAAAGAAACCTCAAGAGGATGTGAACAGTTTTCTTCCCTTGAATCAATATCAAGATTACGGATTCCCCAACGGTGATGAGAAATCCCAGAACTAATACATTATCGCAACGCACAGTGCCTTGCTTGAGAGACAATCTTTAAAACGGTTGTTTGTTTAGACTAGTTCAGATTGTGTCTTCCCAAACAGAGAGACATGTTGGCAATATAAAAAATGTTCAGAGAATTTATTTTTTATTCAAAATGGGGCTTCCATGTTGCATTACTAATAATAAATGCTCGTTTATATTACTGTTGTCAGCCTTAATAAATATACAATTATAATTAACACTTTTATAAATGTTTACACTAAGGTCAAAGGTATATATGTATATCAGAACTTCTCGTTACATGTACACTTGCATAAGACAACACATATTTAATAGAAGAAAATGTGTATTCAAGTGTGTTATTGTGAAAAATTGTTTCAACAGGACAATGTGTGTTTATTGTTTGTTTACCAAAAACCATCAAAAAAATATTACAGTACACAAGAATGGATGTTAACCCCTTCCTGTACACCAGTATCTAATTTTTATCAACAGAGCAGGCATTTTAATCAAAATCAAAAGAAGCATTCTAGTTGCAATTACACCAAAAGATGGCAGATAATGTATTCAACTTGTTCACTAACCACGTGTCAATGTATTTTTGAATGCTACAATTAAACAAAAATGAAACACAACAAATTATACAGGAGGGGAAAATGGTATAGGAAGGCATTAACTACAAGATGAAGTGTTTAATCAAATTTACACTAAAAATGATTTGAAGTCTGCATTGAGTTGTTGTTATTTAGTGGTAAATGTTGCAAACTAGTGTCTTTAAGCGGTTCCCTCAAGATATTGTCTTTGTTTTAAAGAAAATACATGTATTTTCATTTGCATTTTTAAAGAAATGTACATGTATTTATGTTTTTAAATATACCAATAGTGAAAGTTTTGTCCGTTTCTTTGTACATTTAATATGTTTTTAATAAAGTGCTAGTGTTGTGCACTCTGTTACGGTCA

>LsigGluR5145_partial mRNA

GGGATGACGCTGGAAGCCATATCCATCAAAATGGAAGCCAATCCAATTAGAACAGCCCTCAACGTCTGCAAAGACATCATTGCTCGACAGGTGTATGCGGTGGTGGTGTCCCATCCCCAAACCGGAGAGTTATCTCCAGCAGCTGTCTCCTACACTAGTGGATTCTATCACATTCCAGTTATCGGCATTTCGTCCAGGGACTCCTCTTTCTCAGATAAGAATATTCACGTGTCGTTCCTTCGGACAGTGCCTCCGTACTCACACCAAGCCGACGTCTGGGTTGAGCTCCTCAAACACTTCAAATATAAAAAAGTGGTCTTCGTCCATTCGTCAGACACCGACGGAAGGGCCATCCTTGGACGCTTTCGCGACACTGCACAGAGCCTTGAGGACGACACGGAAATAACAGTGGAGAAAGTTGTGGAGTTCGAGCCAGGACTGACAAGCTTTTCTGAACACCTGGAGTCAATCAGTGTTCGTTCAACTGTTTATATCATGTTTGCTGGAAAATCTGATGCGGAGGTGATTTTCCGAGACGCGTCGTTGCTGAACATAACTGGTGCGGAGTATATGTGGATCGTGTCGGAGCAAGCCTTGGCTGCCCAAAACATTCCAGAAGGGACGCTCGGTCTCAGTCTCATCAACGCCACTTCGGAGGCAAAACACATCAAGGACAGTTTGTATGTGTTAGCGTCAGCGCTGAGAGAAATGTACCGCAAAGAAAATATTACCGAAGCACCCGGAGACTGCAACGACTCAGGAGCTGTTTGGGAAACAGGGCGCCAATTATTTGAATACATCAAACTTCAAGTCCTTGAGGGAGGAGAGACGGGGCGCGTGGCCTTCGATGACAACGGTGATCGCAAATACGCTGAGTATGATGTCGTCAACGTGAGAAAAGATGGAGAACGTGTCGTTGGAAAATATCACTATGACAAGGAACTGAAGCGGATGTCGCTGCAGCTGAATACAAGCAGTATTGTATGGCCAGGTGGTCTGACAGTGCAGCCAAAAGGCATCACGGTGGAAACGCACTTGAAAGTGTTGACCATTGAAGAGAAGCCATTCGTTTATGTGAGAGAGATCAACCCTTCATTGGGAGAGGCTTGCGACGCTTCACAGGGAGAAATCCCATGTCCTCACTTCAACAATACCGATTATGAGTTTCGATTGTTTTGCTGCCGGGGTTACTGTATGGATCTTCTTCGTGAATTGGAGAGAAAGATCAACTTTACTTTTGATTTGGCTCTTTCTCCCGACGGACAGTTCGGCAGCTATGGACCAATCAGAAATAGCTCAGGAGTGAAAGAATGGACTGGTCTGATTGGTGAATTGGTGAAGAAGCGAGCAGACATGATAGTAGCTCCTCTCACAATCAACCCCGAACGGGCACAAGTCATCGAGTTCAGTAAACCTTTCAAATATCAAGGAATCACCATTTTGGAGAAAAAGCCATCTCGGTCTTCGACACTGGTTTCGTTTCTGCAGCCTTTCAGCAACACTTTGTGGATCCTGGTGATGGTGTCTGTGCACGTGGTGGCACTGGTGCTCTACCTGCTGGATCGCTTCAGCCCTTTCGGACGCTTTAAGATTGGCAATACGGAAGGAACCGAGGAGGACGCTCTCAATTTGTCTTCTGCTATTTGGTTTGCTTGGGGCGTCCTTCTCAATTCTGGAATCGGAGAAGGCACACCTCGTAGTTTCTCA

>LsigGluR16767_partial mRNA

CAGGGGTGTTGCAATCAACGGGGGTCAGTGGTTCTGTAGAAACTGTCAAGTCAGCATGGATCACTTCAACGGATATTTTGTAGTTGCTGTGGCGTTTTTGGTGTTGGGACCTAATCCATCTTTACAACAGTATGGCAGGGGTGTTGCAATCAACGGAACTACATTGAGAGTAGCCACGTTACTGACAACGCCATTTTGTTCTCAAAACAAAAATGGTCAGTACGACGGCTTCGTGCCCGATGTATTATCGGAACTGGAAAAACAAACTGGAGCTTCATTTTCACTCAAAAAAGCCTCTGATGGAAAGCATGGAGTCCTTGATAAGAGAACCAATATCTGGAATGGAGTCATTGGTGATGTTATCGGAGGGGACGCTGATATTGGATTTGCTGATATAACTGTTACAGCTGAGAGAGAAAAAGCTATTGACTTCACTGTGCCATTCATGGAAGCAGATCTGACCATTATTTATCGGTCAGTGTTGGAGGAACCAGTCGCCTTTGGATGGTTATTGTCTGCAATTTCATTGCCTTTTTGGCTTTCAATTCTCGCGTGCTATGTGATTGTATCGGCCGTTCTATATGCTATCGGAAGAATTACTCCCAAGAAAGGAAAACCCGAAAAGGATACGCCTACTGATTTAACTATATCCGACAGCTTTTGGGCCACTACAAGTTCTGTCTTTCTACGTGGAACAGATGTACCTGTTGACGCATTGTCATCGAAACTTCTGGTGTCCGGCTGGTGGTTCTTTTCTCTTGGGGTAGTTCTCATTCTGATTGTTCTTGAAGTCAGTATGTTCACGTCCGGAGCTGCCTTCACACCTGCGGCTCCAAATTTCTTCGCACACGTAGGATCTGCCACTGTTGGAGTTTTAGGTGGAGGTTCAAGTCACAGACTACTACTGGCACGACCAGATCTGGCACCTGTTTTAAGGCGTCTTGAGGCTGCTGGTTCAATGGGAACTCCGAGTACCGTAGCAGAGGGAGTACAAAAAGTTGTTGATGGAGAACTGGACGGTTTCGTGATTGAATCTCCATCTGCAGACATAATGAAAGCTTCCCATTGTACCTTAGAGACCATTAATGAAGCACTGGACATGAGGTCATTTGCTATCGCAGTAAAACAAGATTCAACTTTGCGGGAAGTGCTCTCTATCGAGTTGTTGAAAATGAGAGAAAGTGGAAAACTGGACGCCATTAGAAATCGTTGGTGGCCAAAAGATCGTTGCCTCGATTTTGATCAAACTACCCTTTCTCCAGCCCGTCTAGGAACATACTATATGCTGTTCGTAGTGTTGTTCATAGTGATCGCCATTGCATTGTTGGCAGCACTGGCCGAAAGATATATGCATAGTAAAAACAATTCTTAGAAAATGGTTCACAAAACGGCCTTCGTGAGAATGCAATTAATCTGATGACTTCATTGAACAAGATTCATTTTATACGAGTCACCGAGTGCTACAAAAAATCAACAAAACAACACGAACAAAAAATTTTATATTGTATAAAATTGTTTATTCATTATATCTGAACATTGTTTATAAGTGTACATAAAAATGTTCAGAATTGAAAAAAAACGCTATTCATTCCACAAAAATGATATTTACAATTCCTTACAAAACAGTATGAAGAATTGAACCTTAATGGTGAATTAATTAATTTAGCGATATTTTTGTTGTTTCTGTTTTGTGTAGAAAGTTTCTACGAATATAAAAATGCAAGTGCATTAAAAAAAACGCTTGAAGGCCTTACGTTTTTGATTGTATATTCTTGTAAATTAAAATGGTATGTCTTGTTGAAGATCATTTACATATAAAGTTGTATGAAAATTAATGTTATAGTGATAACTATGTATTGACACAAAATACATTATATTGTAAATTGGAAAGTCTGGTTTGAAAATGATGTACAGGGCTTTACTACTTTTAGCAGCATTAAATAATACATTTTTAGTTATCCAAAACATAATGAAGATCGTAAGTTTGGTATCTTCATTTGTTACATTGATATCAGTAGGCCTACTCCATGCTCCTTAAGAAACACCTATATTACATATTTCTAAATAAATTAATCTTTAGTTGAATCTCAGGCCTGTAGAATAGATGTCCAACGATGCTTCTCAAGCTAAAAGATTATGCCTTCTCAAATTCATGTTATGGGGTATTTGATAAAATGAACAACAATATGTAAATATTGAATATGTGCAAATATTAAATGGTGGTCTTTATAAATATTTTTCTAACATTTTTAATACTTATTACATTTCTGTGGCATTTTTCACAAAGTAGTATGTATCACAATGTCTTATTGCTCAGGGACATTTCTAAATTAACTGGGAAATAGTGATTTAGTATGTATTGAAACAAACCAGGTGTGAGGATGTTCTTTATCTTTATGTAACATGTCCATAGTAATATTGCATAGAATATGTTTTCTTTCCCTATTTTGTAGAAAGTTTACAAGTAGAACTAACTCCAACCTTTTCCTTTCTTGTTACTTTTATCTGAGTGTGTGTAGTTTATAGAGATTTTAACTAAATGTACCTTAATAAAAGAATATGATCTTTAAGCTTTTGGCAA

>LsigGluR9306_partial mRNA

AGCGGAGGCAGCTTTGAAGACAAGATGATCCGTCTAAAAGGTGCTTCACTAATATCTGTGGTGTTGGTGTTTCTGGTGTTGGCACCAAAGCCGTCGATTTTGCAGCAAATACCTAAAGCTCTCCGAATAGGATCTCTGTTTCTGGAAGACGACAGAGGGCTCCACGACACATTCCGAACTGCAATTGATGCGTTGAAGCGTCGCCCAGACCTCATACCTTCGACAAGAGTCGATTTTGACATTCAGTATGCAAACCCAGGGGACCATTTCCATCTTGGAAAAAGAGCATGCCTTGAGATGCAGTATGGTGTTATGGCTATATTTGGACCCTTCGATAACTCTTTGGACATGCATTTGGCGTCCGTGACATCCGTCTTAAACATGCCTCTGCTTAGTTTTGCACCCGGTGGACCACACGGGTCACTCTCCATATCACTGTATCCATCACAGTACGATTTAGCCTCTGCTACAAAAGACGCCCTAAAATTCCTGAAGTGGACAGAAACTGGGATTTTAATGGATGACAATGGGCCAATCTCTTCGTCTGATCTTCTGGAAACCATTGGTTCATTGGATTTGTCACTTGTTCCTGTTCAAGCTGGAGAAGACCTACGAGAATCACTTGCTGAGCTAAGGGACCGCCACGTTCATCACATTATTATCAATTTAGCTGCCCAAAGGACGGAAGAGTTCATCGAGGCTATTCTAGGCACTGGAATGGTTGATGAAGACTACCAATACTTCTTTACATCACTGGATGCCCACAACACTGATTTTGAGCACCTGCGATTTGTTAAAGCTCAGTTTTATTCCTTCCGGTTGGCACCACACAAAGACTCTTCATTTTATCGCCACATTACTCGAGAATCACCAGGACTTTTGAACTCAACGACGCAGCTATTAGCAGTCGATGCTTTAGAATTACTTGCTCGAGGAATTCGCATTGCTGCGGATAAAGGAGCGGATATGGTCACACCGCCACGGGTCTCATGCTCAGGGGAGCAGACTTGGAGTGCGGGCTTAGCTCTCCACAGTGCTCTGCAACAGGTGAAGTTCCGTGGCGTTAGTGGTCCAATAAGCTTTGAAGATGGTACCAGGAACAGTGTGGATCTGGATCTGCTTCAACTGAGTATACACACAAATGTCACTAGTTTCGAGCGCCTTGGCATATGGTCTATACGCCCAGAAGACAATGCTGGATCGCTTAACATCACAGGAGGGTTTCCGCATGCAGTCAACAGATCCACGCTTATCGTAACAACCGTTTTGGACTCTCCGTATTGTCAACAAGGAGATAATGGTGAATACAAGGGCTTTATACCAGATCTACTAGCCGAACTGGAACGGTTTGTAGGAGCTTCTTTTCAAATTAAGCACGCCGCAGACAACCGATACGGAAGCTATGTTGCCAGAACCAAATCTTGGAACGGAATTATTGGAGAAGTTATGCAAGGGAAAGCCGACATTGGATTTGGGGCGTTAACAGTCACAGCAGAAAGAGAGAAAGCTATTGATTTCTCAACACCGTTTTTGGAAGCAGATCTCACAATTCTTTATCGTTCTCAAACGGTGGAGCCAGTAGCATTTGGTTGGCTCTTGACTCCTGTGTCTTGGCCGCTATGGCTTTCACTTATCGTCTCGTATATGCTCGTGTCCATAGTCCTGTTCCTCGTTGGTGCCATAAGTCCATATCAGCGCCAAGTAGCACCAGGTTCAGGAAGGTGCTGTGGGTCGGGTCTTTCGTTTGCCAATAGTTTCTGGGTTACTACTAGTTCTTTGTTTCTGCGAGGATCAGATGTGCCTATTGTGGCTACATCTGCGAGATTAATAGTATCTGCTTGGTGGTTTTTCTCACTTGGAGTGATACTTATTCTGATTGTTCTGGAAGTATTCGTGTTCACTTCTGGAGCGGCCTTCATGTCTGCAGTTCCAGACTTTTCAGGACACTCAGGGTCTGCACTTGTAGGAGTTTTACGAGGTGGTTCAAGCTTTAGATTGCTGCAGACACGACAAGATCTCATGCCTCTTTTACAACGTGCTGAATCTTCAGGGCACACGGGTTTCCCCAAAACAATAGACGAGGGAATCGAGAGTGTACTGAATGGTAACTTGGACGGCTTCGTTATAGAATCTCCTACAGCAGAGCTAGCAAAGGCATCCCATTGTAATCTGCATACCATTCCACAAGTGCTTGATAAAAGAGCGTTTGCAATTGCAGTACAACAAGGTTCTCCATTGCGTGATAAACTTTCTGTCGGACTGTTGAATTTGAGAGAAAACGGTAAACTAGAGCTCCTTAAAACTAAATGGTGGCCGAAAGGACATTGTAAGGATTATGAACACACATCCTTGAGCCCAGCCAGTGTTGAAGCATATTACATGCTGTTTGTGGTTTTGTTTTCAACGCTGTTCTTTGCATTGCTGGTGGCAGTGGCTGAACGGATATTTCATCGGCGAGTCTTTTCAGCAAAG

>TdomIR1_partial mRNA

AGAGTACAAATGATGCTGTGTTTAATAAAGTGATGGGTAAGTTACATGAGACCGAACAACCATTAACAATACCAGAAGGCCTCAACAGAGTCTGTAACGAACGATTTGCTTTTATTATGTCTACTCTTTCTGCTGCGATGACTCTGCCAAGTGCTTCTTGTCCTATTGTTGATACGTCAACAGATTACTTCAAGACCACTGTGTCCTTGCCCATGAAGCTCAGATTTCCTTACAGAAAGATTATAAACTATTATTTGAAACGAATGAAGGAAGATGGCGTTCTGTATAGGCTGATAATTACTCGAGGACTTCTACGTACTGAAGATTCGGTTACAGAGGAGCTTACCAGTGTCAGCTTACTAGATGTAATACCACTCTTGCTTTTACTGTTGTTAGGCATAATCTTATCCATATTTACCCTTGCAGGTGAACTTTCTCACTGGCATATCCGATCGTGTACAGATAGACATATTGTCAAACCGTTTATTGATTGACTATGTCATACAAAACTTCCTTTAAAATGCGTATATAAACTTTATTCATCTTTTGAGGAATTGTAATAAAAGTTTTACTATCTTGTAACTTAGTGGCATACTATAATTAAAGTTTTCTCTGGTA

>TdomIR2_partial mRNA

CTGCGCTGACGACAATATTGGGAATAGTTTTGGCTCTCTGGATTATGATGTATCTACAATCAGTGCTACTGAAAAGAGGCTTAATTGTGAATAATAGATACAATTTTATCGAACTAGCATTTAATTCCTTTCGTGCTATGTTCTTTGGACACAAACGTCCTGGCCATCCATTACAGGAGTCTGCATATCCACTGCCATCTGCTTGTTGGTTCGTGCTTTTACTGATAATTTTCGCTTATTGCACAGGTAATACGTCATACCTTACATTACCAATCTTCTCAGATTCATTACATACGGTGGATGATCTTATGAAGACTAATATTACCAAGGGACAAACTAAACATACCTCTTTGAGTTATGTTCTTCGTTCTGAGGATTCTAGTGTGGCGAAATTTGCCAACAAGTTCCAGAAAGAGGCTGGGATAGTCCACCGCGACACAAAAACTCAAGAAAATAACTATGCTATACTGGTGAAAACAGTCGGCCAGAGTTATTTGGCAGGAGCAGAAGATTTGGAAGAACAAAGTCACAGTGATTTGGAGTTAATGGGGGAATGTGTTTCCCATTTTTATGTCGCATTTGGTCTACAGAAGTACTCTCCTTACAAGAGACTTTTTGACCGTGCCCTCACGCGATTCTTTGAATCTGGAATCACTCAATTCTGGGAAAAAGACGTTGCTAAGGATTTGGGCTATCATTATATGCGTGAATTTTTTTCAGACATCGTCAGCAAATAGACTTCTGGTGCCACAATTGCCAGTCAACAATTTTCAAGAACTTGTTCTTGTTTTGGTTGTTGGATACGTCAAACCTATTGCAGTCTTGACCTTTCAACAAATGTATATATATTTGTGTGCATTATTAGG

>TdomIR25a_partial mRNA

TTGTGTATGTAGAAACGAAAGAAAGTGGCGAAGTGGAGTACAAAGGATACTGCATTGACTTGATAAACGATATTAAGGATATCTTGAAGTTCGATTTTCAAATTCGWCAYGTAGACCATTTCGGYAATATGGATGACAACGGRAACTGGAATGGAATGATTGAYGARTTAGTWAAAAAGAAAGCTGACATTGCTCTTGGCTCATTGTCAGTRATGGCAGAACGGGAAAACGTTGTTGACTTCACAGTACCGTATTATGACTTGGTAGGGATAACTATTCTAATGAAGAAACCTAAAGCTCAAACATCACTGTTCAAATTCTTGACAGTGTTGGAGAATGATGTGTGGCTTTGTATTTTGGCTGCTTATTTTTTCACAAGCTTCTTGATGTGGGTATTCGATCGCTGGAGTCCGTACAGCTACCAAAATAACCGAGAGAAGTACAAGGACGATGAAGAAAAGAGAGAGTTCAATCTTAAAGAATGTCTCTGGTTTTGCATGACTTCACTTACGCCTCAGGGGGGTGGTGAAGCGCCGAAGAACTTGTCTGGTCGTTTAGTCGCAGCTACATGGTGGTTGTTTGGATTCATCATCATTGCCTCTTACACTGCCAACTTGGCTGCCTTCTTGACTGTATCGCGATTGGACACTCCCATCGAATCTTTGGATGACCTCTCCAAACAGTACAAGATCCAGTACGCTCCTGTCAATGGCAGTTCAGCTATGACCTACTTCCAAAGAATGGCTGACATCGAGAAGAGGTTCTATGAAATCTGGAAGGACATGAGCCTTAATGACAGTTTGAGCGACGTGGAGAGAGCTAAGCTGGCTGTATGGGATTATCCAGTGAGTGACAAGTATACTAAGATGTGGCAAGCCATGAAGGAGGCTGGATTACCAGGAACGTTTGAACAGGCCTTAGAGAGAGTCAGAGCCTCCAAATCATCTAGCGAAGGATTTGCCTTTCTAGGAGATGCCACTGACATCCGGTACCAAGTGCTGACCAACTGTGATCTTCAGATGGTAGGCGAGGAGTTCTCTAGGAAGCCATATGCTATCGCTGTGCAACAGGGTTCACCATTGAAGGATCAGTTCAATAACGCAATCCTGCAGCTGCTGAATAAGCGAAAACTGGAGAAACTCAAGGAAACATGGTGGAACCAGAACCCACTGAAGAAGACATGTGAGAAGGAGGATGACCAATCAGATGGAATCAGCATCCAAAATATTGGTGGTGTATTCATTGTAATCTTCGTTGGTATTGGCTTGGCATGCATCACTCTTGCCTTTGAGTACTGGTGGTACAAATACAAGAAGAATCCTCGAGTCTTGGACACCACAGTAGTTCATGTGAGAGAACAGCCCCCCAACAAAGAGACTTTGCAATTCAATCCAAGATACAACTACCAAACAGACTTCAGACCCCAAGGACTGAACATGGCAGGTGTTACTAACCCATGGTAAAAGCAACGAAAAGAACAAGTATGTCTGTGATTTGCATAGGTAATTTTATGGCAAGGAATCAAAACGTTCACTT

>TdomIR8a_partial mRNA

GTTTCGAGCTTGCCAAGGGATATGAACTGGTGCCTATCAAGAGGTTCTTCCGTGTTGGCACAGTGGAGTCACTCCCCTGGGCATACATGAAAAGAGATTCAAGTGGGCAACCGATTAAGGATCAGAATGGGAAAGAGATTTGGCAGGGCTACTGTGTTGATCTCTTGGATAAACTGGCTGAGAAGATGGATTTCGATTATGAACTAGTGTTTCCAGAGAGCGGAACGTATGGAAAACGATATGAGAATGGTACATGGGATGGAGTCATTGGGGATCTTGCTAGAGGAGAGACAGATATTGTGGTTGCTGCACTCACAATGACATCGGAAAGGGAGGAGGTAGTAGACTTCGTAGCTCCATATTTTGATCAGTCCGGTATCTCTATAGTGATCCGTAAACCAGTGAGGAAGACATCTCTATTCAAATTTATGACTGTGTTGAAACTGGAAGTATGGCTAAGCATCGTTGGAGCACTTTCTGTCACTGGTGTCATGATCTGGATTTTGGATAAGTACTCTCCATACAGCGCACAAAACAACAAAGAAATGTATCCTTACCCATGCAGAGAATTTACCTTGAAAGAGAGTTTTTGGTTTGCTCTAACTTCTTTCACGCCTCAAGGAGGGGGAGAAGCTCCTAAGGCACTATCAGGTCGAACTCTTGTCGCCGCCTATTGGTTGTTCGTGGTTCTTATGTTGGCCACCTTTACAGCTAACTTGGCTGCCTTTCTTACTGTCGAAAGAATGCAGACGCCTGTTGCTTCTCTGGATGAGTTGGCTCGACAATCCAAAATCAACTACACTCTAATGGCCGGTACATCAATTGAACAGTATTTCTTAAATATGGCTGGGGCTGAACAAGAACTGTACAGGGTATGGAAAGAAATTACACTGAACAGCACAAGTGACCAAACCAAGTACAGAGTGTGGGATTATCCAATCAAAGAACAATATGGTCACATTCTCTTAGCTATAAATACGACTGGGCCAGTGGAGAATTCCTCTGTTGGATTTCAGAAGGTTTTGGACAGTGAGGACGGTGAATTTGCTTTCATTCATGATGCTATGGAGATTAAATATGAAGTATACAGAAACTGTAACTTAACAGAAGTTGGTGAACCATTTGCCGAACAACCTTATGCCATAGCAGTACAACAAGGAAGTCATCTCCAAGAAGACATTAGCAAACAGATTCTTGACTTGCAGAAAGAACGCTACTTTGAAACACTCTCTGGTCGCTACTGGAACGCTTCGCAACGAGGTTCCTGTCCGAACACAGATGATAGCGAGGGTATTACCTTGGAAAGCC

>TdomIR76b_partial mRNA

ATTTATTAAAAACTAAATTACATTGTAACGGTTTTGACAACTTAACGTTGTCTTCATCAGACACTAACTCTTACACTAATTTAGCGTAAAAGCCGTTACACCGTAAAACCTGTTATACTGTAATTTAGTTTTTAATAAATATGTGTATATGCTCTGGTCAGAAGTGTTTAGTATTCCTTTAGTGAGTTTCAGAGTCGTGATGGATAGAGTTATATATTACTATCTATTAAGATAAATAAATTAGTTGCAGAAACGTGTGCGCTTACTTCACAACTTTGAAGTTTAAATTTGATATCTTTAAAGGATATGCGCACGACACATACAGGATATAATGTCGTCTCAAGTTTTTCTTTTAAAACTCACTTTTTCCTGAATAAGGTTTATGGATAGTATATTGAACATGATTTTGTGTATGTTGAGTGTGAATTTTAAACTTGATGAACACGTAATTTCTGACAGTTTTTTGCTTTCTTTCTAAGGATCGTGATAATTTATGTGATAGAACCGGGCTTTTGTAGTTCCATTTTCGGTATCTTTAATTGAATACTGACCTTTCTTTTCTTTTTAAAATTTCTTTATGCAATATAGACTTTTCCTGTTTTCCTTGACTAATATAGATTTTCAATTTTCTTCTTTCCTTACATTCAATTAGACTTTTCATTTTCTTTTACTCAATACAGACTTTTTATTTTCTTCCCTTTTTCTTTGATTCAATACAGTTTTTACTTTCTTTCCGTGTTTTTTTTGTGACTCAATACAAACTTTTCACTTGCTTTTCATGTTTTCTTTGACTCAATACAAGCTTTTCATTTTCTTTTTGTTTTCTTTGATTCAATGCAAACTTTCAAACTTCTTTTCCGGTTTGATTTGTTTGAACACAATTTTCTTCCTATTTTCTTTGACTCAAAACATACTTTTCTATTTTTCTTCAGCTTTTCTCTGACTCAATAGAAATTTTTAGTTCTCATTTTAATTACCTCTACACTTACAATCGTATACTATGCAATTTTTTTCGTATCATTACGAAATTTTTACCTGTATTATTCTATTAAAAAAACAATAACAATTCAATTGACTTCTCGCTTTTAATTTTTTTGAAATAATAACAAAACTTAAAAACAAGTTTGCCATTCGTGTACAGAACTATTTTACTATTTTCACACTTTCAGTCATTCCTTCAGGTACTGTGACTAGAGATTAATTTCTTATAACCACAAAGAAAATAGAGCTATATTGCTCGATACAATAGCTTGTGTAGGTGGGCTTTCAAATACATTACAAATGCTGAAACTAAAAACAAGACAGAAAAAATTACAATTCACAAATCTTTCAACACCAAATGTACCTTTCTTCCCAACATTTTCAGACCTTAGGCGGAGTACTGGAACAAGAATGCAGATGGCGTTCTGACAGGGATCAACCGTTGCCCACCATCTTTCGCGTTGATCACAAAGTAATCTCTGCCATTGATCTGCTTTGACTTGTAGTCTTCGCCCATTTCGAGATCCCGTGTAGTGCCTCCGTATACAAACTGGTGTTTGGGGTTGTATAGGACGTCATCCTTGAGTGGTACCTTCTTCTTGTCCTTGTTCTTGCAGTGCTTGATGAGTTTCAGGATCAGTTCTCCGAAGAACGCCACTGCTGCCGAGCTAAAGCCACCGCAGACGACCACGTACGTTGTGTAGAGATCTGAGTTCCTCAATTTCCTTTCTTTGGTTCCCAGATTCAGAGGACATATGTCAGTGCCAGGTAGCAGCTCATGTAGTTTGTGATGGACGATGCCTGCTTCCACTAACGACAGCAATATCGGATTGAACAGCGGTGGTAGATTGCTTCCTTTTGGATAGCCAAATGCCAAGGGTCTGCTTAAGAACGGCTTCGGGGTTGCCACAAACGTACATCTCTTGTTTTCATCAACGTTTCTCCTGGTCTTGTTTAGATAGTCTCTGTAGAGCAGATCCTGGATGATGCGGTTTTCTCGGAAATATACTCGTCCTTGTTTCATGATCTCCTTTTCCATAATCATAGGATCTGGACTGGACGCAAACACTCCATATCTAGAGTTGTTCAGGTAGTCAAACGTTTCATCAAACTTAATCCAATGTTCCATTGGTCCTCCTGTTTTGGCCACCCAGTACATATGTTTCTCGTGCACGTGTTTCGCTGTTTTGATGGGCAAGGTGAACTGAGACAGTGTCAAGAAGGCAGTCAAGTTGGCCGTGTAGAATGATGTTAGGATGGTAATGAATATCCACCAAGTGGCGAATAGCAGCCTTGATGAGTCCGTGATAGGGTTGAGTGTAGATCCTTGCTTAAGAAGCGCACCGTACACAAACCAGATACACGACTGCAGAGGAAAGATTCTGGTAAGCCTTGCGCTTCCTTTGCACAGTTTCACTCGAATCAGGATGATGAAGTATATAATGGGACCCATCACAATCAGGGACAGCAGGATCAAAAACCACACCTCTGTTTGGAAAGGTGCCAGTAGCCCAGATCCTGTGGCCGAATCTTGAGGTCGCTTCATGAGAATAGCAAACCTGTCTTCGGCTATGTTGACAGAGTACTCTATTTCATCCCTGTAGTCGTCATATACGGGCATAAACACTGCTGCGAAATCTACCTCCTTTCGCATTAGCATTCCAACTATCCCGGTGGTTTTGTCTCCGATGATGTCCTCTTTCGGAGTTTTAATCTTGTAGTTAAAGCCATATTTCTTCTTTAGGATGTTAAGGAAATCGAATGCCACTCCTTCTCCGACAAGATTTCCTTGTGCATCTTGTGTTACTACACTTAGCGGCCAATTGTCCTGTAACGCAGCAACTGTGTAATTGTGGTTCCGTATCCAGTAATATTCCTTTAGTTCGTCATTGTAGCATTCATGATCAAGCCAGCTCTCTAATTCAGCCGCATCTTCAGTTGGAGGTTTCCCCGGCTGTTTAAGACATATCAAAAATAGCATTTCAAGTAATTTCTTGTTTAGCGCCATTTTGTTTATTTCATAGCATTAGTT

>TdomGluR19206_partial mRNA

GCCAGCACTATGCTAACCGGAAATGACCGCTATGAAGGCTATGCCATTGAGTTAATTCATGAAATATCTCAGATTTTAGGTTTTAACTACACTTTTAAAGAGGTGGATGATAAAAGATACGGAAATTGGGATAACAAAACGAAACAATGGACAGGCATGATTAAGGAACTTTTGGAAGGGACGGCTGATTTGGCTATAACCGACCTGACAATTACATCTGAACGAGAAAGTGCAGTAGATTTTACTATGCCTTTTATGAATTTAGGTATCAGTATTTTGTACAGAAAACCAACAAAGGAACCACCGAGCTTATTTTCATTTATGTCTCCCTTTTCTATGGAGGTTTGGATCTATATGATGGCAGCCTACATGGGTGTATCAATGTTATTATTTATATTAGCAAGGTGCAGTCCTGACGAATGGAATAATCCTTACCCATGCATAGAGGAGCCCGAAATTTTAGAGAATCAATTTACTTTCAAAAACAGTTTATGGTTCACCATTGGTGCCTTGATGCAGCAAGGTTCTGATATTGCTCCAACAGCTATTTCTACAAGAATGGTGGCTGG

>TdomGluR39276_partial mRNA

CGCTCTTAACCAATCGTTGGAGTTTATCCGTATGTCCTTAAACAATCTGGATACTTCTACTCTTCAATGTCCTGATGGATCAGCTCCAATGCAAAAATTAGATGCTGCAGGTCCTGTTGTCGGAGTAGTCGGAGGTTCATATAGTTCTGTTTCTACCCAAGTAGCAAATCTCCTTCGTCTTTTTCATATTCCTCAAATCTCCCCCGCTTCAACCGCAAAAGCGCTAAGTGATAAATCTCGTTTTGCCTTTTTTGCCCGAACTGTTCCTCCCGATACATTCCAGGCCATTGCTCTTGTTGACATCGTCAAAATGCTCAACTGGTCGTATGTATCTATTATTGCATCAGAGGGTTCTTATGGAGAATCTGGCCTTGAGGTTTTGCATCAGGAAGCAACGGAGAGAAATGTGTGTATAGCTGTGCAAGAAAAAGTTCCTAGTTCTGCCGACGACCGCGTTTTCGAATCAATCATTCACAAACTCAACAAGAAGCCAAATGCCCGTGGAGTGATACTTTTCACAAGAGCTGAAGACGCAAAGGGAATTCTACAAGCTGCTAAGCGACTCAATTTGTCTCGACCTTTTTACTGGATTGCGAGTGATGGGTGGGGAAAACAGCAAAAGCTGGTAGAAGATCTTGAAGATGTCGCCGAGGGAGCGATTACTGTGGAACTAATGTCGGAAAACATTCCCGCATTTGACAGGTACATGACTTCCTTAACACCGGAAACAAATTTGCGGAATCCGTGGTTTGAGGAATATTGGGAAGATATATTTAACTGTGTGCTAGAAAAGAATGTACCTCTTACAACTAACACTACAGTGCGTGTGTGTGAGCGTGGGCTTCGTTTGTCGGAGAAAGTGGGTTATGTTCAAGAGTCAAAAGTGCAGTTCGTAGTGGACGCCGTGTATGCATTTGCCCATTCGCTTGCGAAGTTGCACAAGGATGTGTGTCCTGATAGTCCTGGCATATGCCCGGCGATGGCGGCATATGATGGTGGAAGTTTCTATAACAACTATATTCTGAATGTCTCCTTTAAAGACTTGGCTGGCATTCAGGTGCAGTTTGACAAGCAAGGTGATGGTCTTCCACGATATGACATCCTTAACTACCAAAAACAGAAGAATACTTCAGGATACCATTACAG

>PsicIR1_partial mRNA

GACCGAAGTTGTGTTCTCTCTTTTGAGCGCGGCCGAGCTGACAAATGTGAACGTACTGATGGTCAACGTAGGCGCACCCGAGCAGCTTGGCCTCGAGGATCTGGATTTGAAAAACATCCATTCTACACCCGTTCCAGTCCTGACCACAAGTAGTTCCTTCCACGGCGAGAAATTCAGGTTCTTCGAAAGAGGCGATACGACTGCGAACAGTATAGCGTACGTCTTCGTAAGGAAACACCTCACCGAGCTGTGTTTCGTGGAGTCGGTGTCGACAAGCTACAAGTGGAGCTCGAGAGCGTATTTCGTGCTGATCGTCACGTTTCCTCTCAGAGACGCGCGTGCCAGAATATCTGCGATGCTGCGCCACTGCTGGGAGTCTTGCAACATCTTGAACGTCTTGGTCGTTTTCCACGGCGAAACATCCGACAAAAACATGCTTTCAAGCCAGAAACGCCATTTCCAGATGTATACTTACAACCCGTTCAAAAGTAACGGCGGCGCAGGGGACAAATTGAAGGCGCTGACTCCGGAAACGTGCATGGACGCATGTCGGAGGAAACTGAACAACGCGAACGGGTACGTGTTTGACGTGTCCGCGTCCCCGCACCATCCACTTCTGTGGGAGCTCGTGGACGCGAGCGGGCGACAGACGTTGGGCGGCCCGGACGGAGAGATCATGAGCGCCGTCATCCAGAAACTGAACGGGAGTCTGAGGTTCTTCCGGACCGCCAAGGACTTCTTCTCGGACAGCAAGAGGCACACGAAGGTGCTGATGGCGAACGAGCGGGTCGTGCTGAGGTCCATGCTGAGAAACAGCATCTACCCGCGGCAGCAGGCGTGCATCACCTGCATCGTGCCCAAGGCGACGAGGGTGCCCAGCTACATGAGCATCGCCATGCCGTTCTCGCCGGGAACGTGGTTGCTGTGCCTGTCGAGCGGCGTCGTCTCCGGAGCCGCCTGGAAGCTGGTCACACGCCACGACCGGCGCGAGCCCATGACGAGGACGTTCGTCGACGTGGTGCGCCTGGTGGTCCTCGGCGTGGCGGGGCGGCTGCCCGTCTCCGACTCGCAGAGGCTCCTCGTGCTGTGGTGCCTGGTCGGCAACCTGGTAATGACCAGCCTGTACCAAGGCTTCCTCGGCG

>PsicIR2_partial mRNA

ATGAAGAAATCATCACTTGTTGAGTACGCACTGATAGTTCTGATGCACAGCGGCATAGTACTGTCAATGAACAGTGCTATTTTCAACAGAAGAGACTTCGGCTTGGTGGAAGCACTGGTTGATTATGCTAAGCAAGTATTTGACGAACACCTTCGGAGAAGACTGCTGTTGGTGAATTATGACTCCAGTACGAGAACAGAGGTAGCCAATGAAATCGTAAGGTACTTTAACAATGTTGACCAGCCTCTCATTACGACGATGGTTCCTCCAAAAACTGGAAGAAATGATGAAGTGCGCACTAGCGCGCGTACACTTGCGAGTCATTCACGCCAAGAGTCTTCACCCCTGTACCGACACGACAACGTGGAGAGCAGCATCTCTTTTGTCGTATTCAGGGACACTCTCACCGGAACATGCTTCGCGAATGAGGCTTGGATCCGGACGCCAGTTTGGAACTCCAGAGCGCGTTTCCTCTTGATAATCACATCTCCGTCTGCAGAAAACTTCACGGAAATCACCGAACTGTTTCGAGAATGTTGGAAGAAGGCAAATTTGCTGAACCTAGCGGTAGTTTACTTGGCGTCCCCTTCCGTCTGCAATGGCGGCGAACAGTACAGTATCCGCGCAAAAGAGCTTAACCCTTTCATAGATCCAAATTGTGTGATGAACGTCACGGTTTCTGGGACCAGACGTAAACGCGAACATGACATGTTTCAGGAGAAGCTAGGAAACCTCTTCGGATACGAAATACCCGTTTTTTTTAACCACTCACATCCGTTTTTGGAAGTACGTTACGAGCAAGGAGGGGAGCTAGCTTTCGATGGGCCCGACGGACATATATTTAATACTCTCCTGGCTAAATTTAATTTCACCGCGATGCCCGCTACTAACATTAGCAGCGATTTCCGAATAGAGGACTTGTCGCCAGAAGAAAAACACTACTACAAGAATGGCCTGAATGCAAATGAGGAAGCGTTACTGCCCTCCAATATAAATCGATCAAGCTACCCATTTTACCACGACTACATTTCGGTGATCGTACCCAAAGCATTCCCTCTACCCAGATACATGCACGCGATTCTGCCTTTCACGCAAGGCATGTGGGCACTTTACATATTCTCGCTGATCTCTTTGTGTGCTGCTGGGGCAACCCTAAACTATTTTGATATTCACGAACATGTTCAGGTCAGGAGTTCAGTGTTTGAAGCATTTCAGCTGCTAATTACTGGCACAGCGAATATAAGGAGGCTATCATTTGTTCAGAGACTCTTCCTTACTGTCTGTTTCGTTTTTACTATCGTAGTTAACAATGCGTACCAAGGTTCCATGACAAGTGTCCTGACCAAAACGCTCTACCAACCAGATCTGAATACGTACGAAGATATTCTCAAATCCGATCTCCGAATCGCAGTCTTTTTCGACAGTTCCTACGCTGCCGAGACTAGCAAAGACTTGGAAACGAATGATTCGGACGACGACGATTTTGTGGAGAGGGTCGTAGATAAAATGGAATTCATTGCCGACGACCACGAGGCCCTGATGATGGCTGCGATTCACAGAAATGTCTGCAGACCCACTTACAGATTCTTGGCAGAAATTCTGATTGAACACGAGGATTTCTACAACGCAGGCGAACCTCTGTTGCACATCGCGGAAGAAAAGTCACTCCCTCTGTTAGAAGTCTGGAGTCCAAAACACAGAGATTCTGTGTTTCTCGAGAAAATCAGCCGAATTAGTACGAATCTGGTCACTGGTGGAATGATTGAAAAATGGAGAAGCGATATGCAGTACGCAGAGCTGATTTCCAGACACAGGAGAAAGACGAGTTTCCGAACGGGTATTAAATTAACGCTAGAGCACCTCCAGGCTGCATTCTTTATACTTGGAATTGGTTTGCTTATAGCTGTTTTAATGTTTGTCGCAGAAACGGTATTCAGCTTAACAGATGTACAGCTGTGTTTCGTAACATTGAAACGGTTTCTTTCCTCAAGTAAATAAAATTATTTTCGTGCGATGTGGCGTCTTGGTAGCAAGAGCGGCGTAAAAATAAAACAAATAACTTAATATACATCAATATCATACAAGTTGCGGTTTTAATTTAAGGAAAAAATTGTTTAGTGTCCATTACAAAAAAATCTATAACCGCATTGTTTAAAAGTTAGTTCGATTCTGAAGTTTGTGACTTAAATTAACTGACTATGATACAGGTTTTCATTATTTCCTTTGAGAATAAATAAAGGAAAACTGGTGAAATTATGATTAAATGGTAGTATTTCAAGATCTCTTTCCAAGTAGTAATAAGTTGAGTGAAATATTTAGTAAATAAATATTAATA

>PsicIR3_partial mRNA

GTTGCTGTGTATGTTATAAACGAGAGAACAACCTGGCCAGCCAGCATGCCCCAAGAAAGCACAGCATTTGTGATACTGTCTCAGAGCGGTGAGGTTGATGTATACATGAATCATTTAGCACAAGTCTGTAGGCTACCTTTGTGGACCAGCTGGTCACAAGTGTTTTTCATTGATGCTAACTTGAAGGACAATCACAAGGACATTTCGTTCGCGCTACTCAAAGCCAGCTGGAAGAAGTTCGAAATTTTAAATTGCCTTGTAATATATAGTGCTACATGTAAAGATAAAAGCAGAAAACTTTCTGTCGCTAAAAGCTTAAATCCATTTGTGAGTCCAGGTCGGACAATAAATGTGAAATACAAAGACGACCTGTTTGAAAAATTCCCGTTTGATTGTATGGGGTATCCCGTGAAATGTACTGCGTTCAACTATCCTCCTTTTGTTTTTGAAGAAACTACTCAAAATGGGATTAAAAAACTGGGCGGAATGATTGGTACTATTATAAATATAATTGCGATGTCAGCGAATGCAACCACCACAACTGACGTAACTACACTGAAGGATACATCAAAATGGGTACTGCCATATTCTTTCTACAACACAACTGATGTCATCTATCCTTTCGCTCCTCTACAAACAAGTAACTTGAAGGAATCCTCTTACCCATTCCTCCATGACAAACTTGTTTTACTGGTTCCGAAAGCAGAGCAAATTCCCAAACGGATGTGTGCCATATTGCCGTTTAATTATGAGGTTTGGATTTGTTATTTAGGTGTCTACGTTTTGCTTGTTTTGTTAGGGAAGGTATATTCACGCTACACAAGAGGAAACACACTTGCTTTATTTTCCAGTTTGGAAATATTTCGATTGTTTTTTACTGGAATGTCACAACAACGTGTCACCGCAATTGGCCTAAGAGTATACTTAGCGTCTGTAATGTTGTTCAATATTGTCTTAAATAACGCATACTCTGGATCATTTACCAGCTACCTTAGTATTCCGCAGTATTACTCCGACATAAACAGTTTTGCTGATATCGATAGATATTGTTTATCGATAGGTCTGTCTCTCAAGTATGGTACAGATGCTGCATTAGACTTTTCCAGTATTGTGTCACAGCTTGATACTGACGATAAAATGACCGCGAAGTTTTTATATCGCCTGGAAATGGTTGAATCACTAGAAGAGGGCGTACACCGTGTGAAAACTGAAAGAAATTTTTGTCTTTTGCATTTTCGGACTGGAGCAAAATTCGAAATCAGCAAACCAGAAAACATTGCAGGCAATCGACCATTATTACACATTGCAAAACAGGAACTCCTCCCGTTACATGCAGTAAGCATTATGCGTTTCAACTTTCCCTTTTTATATTCCTCCAAAAGCAGGCAAAGGTTGGTGATAGAATCAGGCATGGTAAATATGTTGCTAGTAAACTTCGACAGAGAGTTACTAAAAACTCATGAAAACAATGACGTTGTAGTTCTCAATTTGCATCATATGTGCATTTTTTTTATTATTCTTTTCAGTGGGTGCCTTATTAGCATTATCGTATTTGCATGCGAGTTAATCATGGGAAAAATCGCCTTCAAAAACATTTTTTCTAATGGCAATAAGAGAACGTAAACATTTTAATAGTGGTG

>PsicIR4_partial mRNA

TGTTGAATATATGGTTGCATCAGCCGCGCTGTGCCTGATTGTGTACAATGCTGTAATGATAAAACATTATGCAACTTGTGTTCTTCGTCTTGCTACTCCTGGTTGTAGCGACTCTCAATGGCTCCGTTATCACGGGTCGGAACAGATATGAAAGAAGAGCTCAGCAGTTCGTCATGGAGGCGACATCAGTAATACACGAGTATTTAAGGGATGTTGCGGGAACAATGGTCTTGTACACTGAAGACCACCAGGGCACCACACAGGACCTCATAATAAGCTACTTGTATTCAACAACAAAGCAACCTCTGCTTGTTTTCAAGAATACTGCACAATGTTGTCCTCCCATTGCTAGTCAAAGTGCAGGATTTATAATATTATCTGCACGAAGAGACGTTCATGCTTTCATTCAATCATTGGTGCATGCTTCAGGACTTCCTGTGTGGAGATCGTGGTCACCAGTAATATTCGTTGACACTAATTTAAACGAAACTCGCTCTGACATCACATTTCCACTACTCAAGACCAGTTGGAAGAAGTACGAGACTTTGAACTCATTGGTGATTTGTGTGTCTGCGAACAGTGATGACTCTTATAGGCATGTTGTGGTCAAACGATTCAACCCATTTGTCACTGCAGGAAAAACTGTCACTGTAACAAACAAGCAACTTACAACTCAGAAACTGTTCTTTAATGCCAGGGGATACCCGATAAAATGTCCAGCATTTAATTACCGGCCATTAATGTACGAAGTAACAAGGCCGAATGGGGAAAAAGCTCTTGGTGGCATTATTGGAATATTTGCTGATATGATTTCTGAGCTTGGGAATTTCACCACTACTACACAACAAGATACTTTAAAAAATCAGTTTCAGTGGGTTCTGCCTGAAAGTTTTTTCCACGAAAGATTTGACGTTAAGTATCCAATCACAATTCTACAGCCAGATATTTTAAATAATTCTCTTTACCCATACATGATAGATAATCTCTTGCTGATAGTACCAAAGGCTCAGCAGGTCCCGAAATGGATGACTGCAGTACTACCTTTCACTTACACATTGTGGATACTGTATTTGTCTTGTTACCCAATACTGGTTGTGTTAGGAACTATTTACTCGCGTATTTCAATAAATAAGTCAAAAGGTTCTTTCCATGCATTAGAAATATTTAGAGTGTTTTTTCTTGGGATGGTGCACCATGAGCTCAAGTTTGTGTGGTTCAGGTTATATTTAGCTTCTGTGATGATTTTTAACATTGTACTTAATAATGCTTACACTGGGTCGTTTACCAGCTACATTAGTATACCTCATTACTATAAAGACATCAACACACTTGAAGAGGTTTTCCATCAAGGTTTCTCTGTGGGAATAGCTGTTAAGTATGGTTCCAATGTTAACTTCAATTATACTAGCATGATATCTGATTTTGATTTCAACGATGAACTCACATATATGTTAATGAGCAAATCGATGACAGTGACATCGACAGAAGATTTTTTAATTTCTGCAATAACCAACAGAAATTTAAGTCTTTTTCATGCTTCAGTGGCTGGGATGTACGAAATGAGCAAACCAATATATTTTCGGGACGGTCGACCATTACTGCACATGATTCCCCAAAGTTTCCTTTCTTTGTATCTTGTTTTTGAACATCGAAAAAACTACCCTTTTGCCAATATACTTGATCGTAATTTGCAGCTTGTAGTAGATGGTGGCTTCATCACGAAACACATTTCTGATTCAATTGAAATGGTACACACAAGTTTCCATTCATTTAGCAAAGATTTCACTTCCCTGAATACGGAGCATTTACAAAGTTTCTTCTACCTTCTTTTATGCGGCTATATTATCAGTACTGTGGTCTTTGTGGTCGAAGTTTACTATTTGTAGAAGGATGTAGGGTAGGTATTAATTCTTAAGTGATATTCTTAAGAGGAAGAATTAATTGTAAAGTAATTTTCTTAATTTATATGATTATATTGATTACCAGTAAGTTCAAAACCAAATGGAAATATGAGCACTGTTGAGTCATCATAAAGACATTTCAGAAAGGCAATAATAGTGACATTTTATGGAGAACAATTTTCTAAACATGCCTCTTTCTTTTGTATCTATATTCTTACTATATATATTTAATGCAAAACTTATGATTGGAAAGTAGCTTTAGAATTTTCGAAGAATTATGTGCCTATTAATATTAACTTACATGTATTGATGACAGGATGTTTTTCATCCCTATTTGTGTATGAAATGTGGAACTAGAGACAATTGTTACAGCTGTTCCACAGTACGAGACTGACTCTACGGGAACTCTGTTACAAAATTGGTTTTATGTAATTATCACTGACTATTATGTGAGATGGGTTGTTGGTGGAGCAGAGATATATTTCCTTTGTAGAGATAACTATATTAACTACAGTAAACTACTTCACTATTTGCCTCGTGTCCGACATTCCAATATGCCAACATACACCCAGGTTGAAACCTGTACTGTCTAAACTGAGTAAACTTGAGTAAACCGTAAATAATCTTTTTCAAAAATGGTGATACATTAGAGAAGAAAAAATTATCATAAAGTAATAAATATTTAGTGAACATATTTTCACCAAGGCTTGTTAAATATTTAATCACTGGTTAGCTCATTTCTTCTTCACAAAATCACAGTATAGGTTTTTGTCCAG

>PsicIR5_partial mRNA

TATGAATTCATTAACAATTTACTTTCAGAATTTATTTGAAGTGAATCAATAAAAGCATTTGATCAAACTTAACTTCATGCTGTCTGCCGAGATATGTACTATGTTCTATTAAAAACAATACATACCAACGCAGGGCATGGGACACATTTTATGGCACCAAAATGATCAACCTAATAAGCGCCTGAACGACGACTATAACTTCAGCAACTTTGAAGAAACAGTCGGGTTCCCGGCACACGACAGCAACATGAAAGCGTTCCAGTACATTCAAGAGCTCTGCAGATTCAAGACGACCTTGAAAATTACAGAAGAACTGGCTAGCCTGTCTGTCACAGATAACGACGGGATTGTCGGAATGCTGCAAAGAGAAGAGTTTGATGTCTATGCCACTCTGTCTGGTATTCTGCCAGTAACATTCCAATACGTGGATTTTCTGCAACCTATCACGCAATTCCAGCCGGTGATAGTGTTCCTGCAGCCGACGGTGCCCACGCTGCGGAACGTGTTCCTGCTGCCGTTCACCCGCAATGCGTGGCTGGTGCTGGTCGGGACGGTGGTGCTCAGCACTGGCGCGCTGCTGGTCAGCACCACGCTCGCAGACTATGGCCAGCACTGGGGAGTGTCCGACGTAGTCCTCATCGTGCTCGGCATCATCTGCCAGCAATGCATGGAGCAACTGCCACGCAGCTTGGCGGCGCGCGCCGTGCTCGGCGTACTGATGCTGTCCACAATACTCGTGTGGGGAACGTACTCGGGGGTGGTGATATCCCTGTTC

>PsicIR6_partial mRNA

CTGGCCGCGACTCAACCTCAGTGTGGACAGCGAGCTGTGGTGGGCGTGCCCAGGAGACGACGACACAATGCAGCTGAGGGACGTGTACCGCCCGGGGCCCGGCGTGCCTCACGTCGACCGCCCGGCGGGCATGTGGACCCGGTCCCGCGGGCTGGTCCACGAGCTGACCGCCTACAAGTACCTGCGGAGGGAGGACCTGGCCGGGTTGCGACTGCGCACCGGCATTACGATAAACGCCCCGCTGGAGAATCCAGAAGTAAATTTGCTGAAGTTGGAGAACAGACAGTTGGATGCGATGGCTACCTACAACTACGGGTTGTACATCATGCTCCAACAGCAGTTCAACTTCACCATGGAGCTGGTGGTGACGGACAAGTTCGGCTTCGTGGTGGAGGACGGCCGCTACGACGGGTTGACGCAGCTGCTCGGCGAGAGGCAGGTCGACACAGCCATCTCTACGCTGCTCATGAACAGGCCTCGCATGAGCTTCGTCGACTACAGCACCGGCTACGGCTGGGAGTTCCACATCTGCGCCATCTTCCGGCACCCATCTGTCTCCGGCGGGGAGGACGCGCTCATCAAACCATTCTCCGGCAGCACGTGGCTCTGTACGTTCCTCATGTGGCTGGTGATCGCGGCATTCCTCAAGCTGCTGGCGTGGATTCAGCCGCACTTCGCGGACGTCACCGGCGAGGACCACGAGGAGATCCCATCCTGGAGCGACCTCATGCTACTGGTCGTCGGCATCGTTGGGGAGCAAGGTACTTGGCTGGACTCGAAGTGGTTCACCTGGAGGTTCGTGTTCTTCATGATGCTACTGCTGACGGTACTGCTCAACACGTTCTACGCCGCCATCGTGGTGTCCACCCTGCTGAACCAGCAGGCGCAGACCATCAACACGGCCAGGGACCTCCTCGACAGCCACCTGCACTTCGGGGCCGAGGACATCATCTACAACAGGCCCTACTTCGAGGTGAATTCAGATCGACTCATCCAAGAACTGTACAAGAAGAAGATGGCGGGACATACCAGCTACTTCCCAATGGAGAAAGGAGTCGCCAAAGTCCTGAATGAGGAGTTTGCATTCCACACGGAGGCTGTGCGGGCCTACCCTGTGATCGAGAAGATGTTCCCGGACGAGAAGAAATGCGCTCTGAAGGAGATCGAGTTCTTCCCGGTCGAGATGGGCTTCTTCGCGTTCCCGTTCATGTCTCCCTACAAGAAGATGTTCACCTACGGGCTCCGCAAAATCGCACAGACCGGCCTGTGGCAGCACCAGAACCAAGAGTGGCGCTCCTCGAGGCCCACGTGTGCGTCCACCGGTGCGGAGGTGATCAGCGTGGCGCTGCCGTCCCTCGCGCCGGCGTTCGGCCTGCTGGCCCTCGGCTGCGCGCTGTCGCTCGCCGTCCTGGTGCTGGAGAACCTGCAGCATGCGAGCCGCCACCGGCCACGTTTCCGCACTTCCGACAAGCAACTGACATGACCTGCACCGCAGTCACCTAGCTTTCTAGGAGCACTCACCATCCTGGCACTGGAGAACCTGCAGCGTGCGAGCCGCCACCGGCCACGT

>PsicIR7_partial mRNA

GGCCCTTCTCTGGAGCGCTGTGGCTCGGCATGTTCTCCCTGTGGCTGCTCATCGTCAGCTTCCTGCGGCTCCTGGCCTGGCTGCAGCCGCAGTACGCCGACATCACTGGCGAGAACCACGAGGACATCCCTACCTCCAGTGACCTCTTCCTGCTTGTCGTCGGCATCGTGGGCGAGCAAGGAACCTGGTTGGATTCTCGGTGGTTGACCTGGAGGTTCGTGCTGCTGATGATGCTGATGTTGACCGTCCTCTTGAACACGTACTACGCAGCTAATGTGGTATCCACACTGCTCAACCAGTCTCCCTTCACCATACGCACTCTCAAGAACCTCATCGACAGCTCTCTGCAGTTCGCGGCTGAAGACGTCATTTATAGCCGATCTTACTTTGAGATCACAAATAACTCACTCATCCGGAAGCTGTACACAAAGAAGATGGCTCCTTGGGGCGGGAACTACATCAGCATGGAGAAAGGTCTGGAGAGGGTCCAGAACGGGGATTTCGCATACCACATGGAGGACGTGCGGGCGTACTGGGCTATCGAGCAGCTCTTCCCAGACAGCAAGAAGTGTGCCCTGCGAGAGATCGAGTTGTTCCCAGGGGAGATGGGCTACTATGTCTACCCCTTCAACTCTCCCTACAAGAAGATCTTCACCTATGGGCTGCGGCGGGTCGTGGAGGTGGGGGTGTGGGACC

>PsicIR8_partial mRNA

GGCCCTTCTCTGGAGCGCTGTGGCTCGGCATGTTCTCCCTGTGGCTGCTCATCGTCAGCTTCCTGCGGCTCCTGGCCTGGCTGCAGCCGCAGTACGCCGACATCACTGGCGAGAACCACGAGGACATCCCTACCTCCAGTGACCTCTTCCTGCTTGTCGTCGGCATCGTGGGCGAGCAAGGAACCTGGTTGGATTCTCGGTGGTTGACCTGGAGGTTCGTGCTGCTGATGATGCTGATGTTGACCGTCCTCTTGAACACGTACTACGCAGCTAATGTGGTATCCACACTGCTCAACCAGTCTCCCTTCACCATACGCACTCTCAAGAACCTCATCGACAGCTCTCTGCAGTTCGCGGCTGAAGACGTCATTTATAGCCGATCTTACTTTGAGATCACAAATAACTCACTCATCCGGAAGCTGTACACAAAGAAGATGGCTCCTTGGGGCGGGAACTACATCAGCATGGAGAAAGGTCTGGAGAGGGTCCAGAACGGGGATTTCGCATACCACATGGAGGACGTGCGGGCGTACTGGGCTATCGAGCAGCTCTTCCCAGACAGCAAGAAGTGTGCCCTGCGAGAGATCGAGTTGTTCCCAGGGGAGATGGGCTACTATGTCTACCCCTTCAACTCTCCCTACAAGAAGATCTTCACCTATGGGCTGCGGCGGGTCGTGGAGGTGGGGGTGTGGGACC

>PsicIR9_partial mRNA

GCAAGGTTCCGACTTGGATTCGAAGTGGATCACGTGGAGGTTGGTCTTCGCGACGATGCTGATGCTGACGGTTCTTCTCAACAACTACTACGACGCGTGTATGGTCTCTTGGCTGCTGTCAGAGACGCCCAGAACAATCACAGACCTCGAGAAGCTCGTACACAGTCCTCTCCACTTCGGTTGCGAGAACCTCAGCTACATCATCCCATACTTTGTGAACAACGACGACGCTTTGATGCGGGAGCTGTACACCAAGAAGATGCTGCGTGAGCGCGGGGTGGAAGGCGCCTTCTACCCGCCGCAGGTCGGCGTGCGCAAGATGCTGGAGGAGAGGTTCGCCTTCCACGCGGATCCCAACCACATGTACGGCATGATAGAGGCCACGTTCCCGGAGGACGACCGGTGCGACCTCACCGAGCTGCAGATAATGCCGCCGAGGAGCCACCACCTGGCGCTGCCGTCCCGCTCTCCCTACAAAGAGATCTTCAACTACGGGGTGAGCAGGTTGAAGGAAACTGGTGTGATGGATTACACATGGAAGCACACCCGTCCCACGAGAACCAAATGCTACTCTTTGCCACGGGTGCGGAGTATGGAGTTGTCGTACACAGCACCTGGTTTCACCATGCTCCTGATAGGGGCGCTGCTCTCGCTGGTGGTGTTTGCTGGAGAGTTTCTCTGGCACCAGAGGGAAAAGCACCGGGACTCGAAGATAAAACTATTCATAGAAACAAGTGACTAGAAGACATAAAAAATAATTCATTATGAAAATAATAAATCTTGTTGATCTTCAAGAGTTACTGGGTTCTTAAAGTCGGTATGACGAGATATCACACGTTCAGTTTGGGTTAAATGTGTACCTCAGTTCATTAATCAAGAACACTCAGTCTGTATTTCGTAGCAATTAATGCTAGAAAAATGCTTTATGATTTATAACATTTCCTG

>PsicIR25a_partial mRNA

AGCTGCGGCCCCGAGCGCTTGCCTCACACCAGGGGACCGCCCCGCCGGCGCCTGCGGCAGAGAGACTTGAACATGGCAGCGATGTACGCTTTCTTGGCGGGGCTGACTCTGCTTGGCGTCGCCTACGCTGCGGACATCAACATAATGTTCGTGAATGACGAGGGCAATGTGGTTGCCGATAAGGCAGTAGAAGCGGCATTGGAGTACCTAAAGAAGAATGGTGGTGGGAACTTCAAGGTGGACAAGGTGTCGGGAAACGGCACGGATGCTCACGACTTCCTCGACAAATTGTGCAAGGTGTACAATGATTCGCTGGAGGCCTCGCAACCGCCACACCTCGTGTTAGACACCACCATCTCTGGCGTACCCTCTGAGGTGCTCAAGTCCTTCACCTACGCCCTTGCGCTGCCCACCATCTCTGCTTCCCACGGCCAGGAGGGCGACCTCAGGCAATGGCGTAACCTTGATCCAGAGAAGGAGAAATACCTGATCCAAATAATGCCTCCTGCTGACATGCTGCCCGAGGTCATCCGCAGCCTTGCCTACACGCAGAACATCTCCAACGCTGCTGTGCTGTTTGACGACTCCATAGTGATGGACCACAAGTACAAGTCGCTGCTGCAGAACATGCCGACGCGCCACGTGATCGTGAAGGCCGGCGAGAACATGGGCATCAAAGAGCAGCTGGCCAAGCTGCGAGGGCTGGACCTCTTCAACTTCTTCGTGGTCGGCCGCCTCAGCACCATCAAGAAGGTGCTCGACTTCGCCAACATCAACAAGTTCTTCGCCACACACTTTGCTTGGCACGCCATCACCCTGGAGTCAGGTAACCTGAAGTGCAGCTGCGCGGATGCCAACATCCTGTTCCTGCAGCCAGAGCCAGAGGCCGACTTCAAGGACCGCGTGGTGCAGCTGAAGAATGAGTTTGGTCTGGATGAGAAGCCGGAGATCACGGCCGCCTTCTACTTTGACTTTGCTGTCAACGCGCTCTCCGCTATCAAGAGCATGGTGGATGGGGGCACCTTCCCAGCTGACATGAAGTACGTGACATGTGACGAGTACACGGAGGAGACAGCACCCAACAGGACTGGTGTGGAGCTCATGGAGGCCCTCAGATCGGTGAATAAAGCACCATCATATGCTCCGGTGCGCCTGGAGAACAATGGTCACAGCTTCATGGAGTTTACTCTGCGCATAACAAAGGCAAACATCTTGAACAGCAAGCTGGAGACGTCTGAAGAGATTGGCACATGGAAGGCGGGCTTTGACGAGCCACTGCAAGTGACAGACGAGGGTATCCTCAGCAACATGACTGCCATCACTTTCTATCGCATTGTAACTGTGGAGCAAAAACCATTCATCATAAAGGACGGGGTGGACGAGAAGGGCCGGCCCAAGTTCAAGGGCTACTGCATCGACCTGATCAACGAGATCCGCAACATCACAGAGTTTGAGTACGAGATCTTCGAGTCGCCGGATGGCAAGATGGGCAACATGAACGAGAAGGGTCAGTGGGATGGCATGATCAAGGAGTTGATGGAGAAACGGGCAGATATTGCACTGGGCTCACTGGCAGTGATGGCAGAGAGAGAGAATGTGATAGACTTCACTGTGCCTTACTACGATCTGGTGGGTATCACAGTGCTCATGAAGAAACCGAAGGCACCTACTTCTCTGTTCAAGTTCCTCACAGTGTTGGAGAAGGACGTGTGGCTCTGCATTCTTGCCGCCTACTTCTTCACCAGCTTCCTGATGTGGGTGTTTGACCGGTGGAGCCCGTACAGCTACCAGAACAACCGGGAGAAATACAAGGATGATGATGAGAAGCGAGAGTTCAACCTGAAGGAGTGCCTCTGGTTCTGCATGACGTCGCTCACTCCACAGGGAGGTGGGGAGGCACCGAAGAATCTCTCTGGACGGCTAGTCGCCGCCACTTGGTGGCTGTTTGGCTTCATCATCATTGCCTCCTACACTGCTAACCTGGCCGCCTTCCTCACCGTCTCTCGTCTGGACACCCCTGTCGAGTCTCTCGAGGACTTATCCAAGCAGTACAAGATACAGTACGCGCCACTCAATGGCTCCTCCGCCATGACCTATTTCCAGCGCATGGCCGACATCGAGAACAGATTTTACGAGATCTGGAAGGACATGAGCCTGAACGACAGTCTGACGGATGTGGAGCGGGCCAAACTGGCAGTGTGGGACTATCCGGTGAGCGACAAGTACACCAAGATATGGCAGGCCATGAAAGAGGCCAAGTTCCCCAACAACATGGAGGAGGCCATGGATAGGGTGATGGCCTCCAAGTCCTCCAGCGAGGGATTTGCCTTCATTGGGGATGCCACGGATATCCGGTACCTTGTCTTGACCAACTGTGACTTGCAGATGGTTGGTGAAGAGTTCTCCAGGAAGCCGTACGCCATTGCTGTCCAGCAAGGATCACCTCTCAAGGACCTGTTCAACAATGCAATCCTGCAGCTGCTGAACAAACGCAAGCTGGAGAAGTTGAAAGAAATCTGGTGGAACCAGAACCCGGAGAAGAAGACGGACTGTGAGAAATCGGACGATCAGTCAGATGGCATCAGCATCCAGAACATCGGTGGAGTGTTCATCGTGATCTTTGTGGGTATCGGCATGGCGTGCATCACCCTCGCATTCGAGTACTGGTGGTACAAGTATAGGAAGGATCCCAAGGTGGTGGACGTGACAGCGCAGCCTGCTGTTATCCGCCAGTCTGTCGCCCCGTCCAAGATTGACACTGGCATCACCATGGGCTTCCGCCCACGCCAGCCCTACCCTGACAACTTCAGAGGACACGGCATGCCACTAGCTGGTGTCGCCAACCCCTGGTAGGTAGGCAGCCAGCTTGCTGACTCCACAGCAGCCACTTGAAATGATGTCATCTGAACCCGGCATGGCCGTGTGTCGTCATAGCACAGCATGCAGGAACAGGAAACATTGCAGTTTGTTTTGTCACACTCCAAGCACTTACACATTTGCTTCATTGCATTCTAGAGCTAGCAAGCTAGACTTGACCACTATAACAACATGTTGAGTCAGTGGTTCACTGAAGAATTGCTGGAATAGATGTACTTGTTAGGTTTTGTATTTCTGTTTGCTGTGTATATACATGGAGAATTATTTCTTAGCAAGAGATGGAGCATTAATAAGCCCAAATAATCATAGTAATGATAAAAAATTATTTTCCAATTTGGTTGGCTCGATGGCAGTTCTGCTACTCTAACGCCAAACATTTGTGATTCATTTGGACTAGCAAAGGTGTGAAACAAGTATTTCAGCCAGTAATGAAGCAGTCAGACACGCACTACAACTTTTTGAAGGAACAAAGTGCCGCAGAATTATGTGTTATTTTCCAGAACTTTTAATGGTTACATTGACAAATAAGGAACAGCTTTACATGCAGACATCAGACCAGGCCTGTTTCCAGGTGTAGAACTTTGGGTGAGGGGGAGCTGTCAGATGATCACCTGGCTAATAGCTAAACAATTTGTTACTTGCCTTCCCCATAATGTACTTGGTGGCTGTAAACAAACACTGTAACAACCTAGTAGCTGCAGCTTCTAAATGTGCCAGATGGCAGTTGTTCAGTTACAGCTATTTTGACATCTCTGTTCCAGAGAAAAATAGTTATTCTGACTCTGTGTTTAAATTTGTTCCTTTTAATCTGAAAGTCCGTGCAGAAACAATAAATGAAATGTAATTAATATTAAAGAGATTTATATGATATTTATTTTGAGTAAATTTTCACTGGTAAAATTTTTTGTATTGCGAAAATATCTTCAAGGGAAATTAAATAATTATTTTGTGAAAATTTAGGGTTGAAGCACTAAGAAATATTTTGGGGGCCCTTTTTAGTTATGGGCCTGCACGAAAAAAAAAAACAAAAGGAAAATGTTTTTTATGGGCAAGCAGTCAACTTTTTGTGTGAGAAACCACTGCTTGTAATGGATGTGAAATTAGACCCACAAGAACTTTCTATTTCTAAAAGTGCTACACAAGTTTTCCCAACATTGCCCATTGTGTGATTGCTTTCTTCGTGCTTTTCACAGCTGTTGAGGATAAGCAAAAGTAAGTTCTTACAAATGTTCAAAAAGACTTTTTCAAGGGTAAAAAAACTTCATTAGAATATTCTTTCACTTTTCAGAAAACAAAACCATGTTTATCTGTAATAGAAAATGTGCTGTAACTGTTTGTATTCTTAAAAACAATTGATAAACGTTTAAAAGGAAAACCACATATTTTTTAAGAGTAAAAATTTTGCATATAGGTTTGGTGAGGATTTGAGCCATGCGTGTACTTTTATGCTGACATGTTGTGGTCTCAGCCAATGACAAACCAACAGCAAACAGTTCACATGCACCCAATCAGACTGACTTCTCACAAAGATAATGCTTAGTGAGGAAATAGATATACCTGACTGCTAAGAGGACTGAGAAGTGTAGCCTACAGTTCACTGAATCTCGGACGTTTTCGTTCTTGCAAACAGTTCAGCATTGTTCTTTCGTACAGTAGTTTACAGGAAACACTTTCTCTATTAGTTGCAACATTCAGAATATTCCTAGAAATATTTTTCACTTGACCTCAATATAAATTGGTTTCTTTGTCTCTGAAATATTTAATGGGATAATAAAGTTGCAGTTGTGCATAAAAAATAGTCTTCGAAACATTAGACAGTATTTAGTTTGCAAGAGCATTTTATATAAGTTCATTGTGAGTTTGTCATGTTATACAAATGAATAAACAACAAAAAAAAGAGTTGATTTTATTTATGATTGATTTTTAACAAAAGTCGAAAACCTATTTTATGTGAAAATAATACAGTTCTTTAAAACAATGTAATAAACAACTACAAAACTACTCACTAATTTTTTTTTATTTTAAATAGGCTTTTCAGATGCATTTGACCTGAACAAAAACAAAAAAATTCAGTTACAATTTCTTTTGTGTTCAAAACATATATTTGTTATTGATCTCTGTACCACTATTGCCACTGGGCTTGCTGCTGCAGTACCATCTAAACATCGCTTTGCGGTGAGTTTGTGTTTCAAGGAATCCAAAACATTCATCAGTTTCCTGTTCTTTGCTGTCATGAAATTTATTGATAGCACTATTCTCACGAAATTACACAAGTGATATTTATCACAAGCCTTGTTGCAAGCTTGATCAGCATCCTATTTACAGGAAAATTGTATATTCACAGTTTTCCCAGTAAGACTTTACACATTTCACTATCGTTTACATTATAAAGTGTTTGATACAACACTCAGGATGCTACAAACTTTTTCACTAGTGGCTACAACTTATATTTCCTTATGTAACACATGTTCTTTGTTGGTACTGAGCATAAGAGCAGGGTTGTACATATTCAAGTACTGTATGAATCATACATTTGATAACTTTGTAACTCTGTCAAATTTTCATTTTTCATTAACTCATGGTTAAACCTGTGTGAATTAA

>PsicIR8a_partial mRNA

TTGGTTTCTGAATATTATTTACTTTCGTAAACTGTATTACAATAATTTAGCCCAAACACATAATAGTGTAAACATATACCACTTTTTGATTAGCCATTGGTTTTTCCTATGAATGATGACGTCACGCAAAATCGTGAATGAAAATGGGTTGTATTCAAATGCTCTTAAGAAAAGTAGGTGATTACAACGACCCATATACTTACTGTGTAAGTGTATTTTGTGCAAAAGGACATTATTTGTATAAATTAATGATCATTTAGTCTGCAAGCATGTTAAAAGTCCTCCGGAACACCGTCAGTTTTTTTTTTTCAGTAAAGCTGGTTGCGTGGGAACACTGAGATGTAAGACACGCGCGGCATCACTTTGTCGCCGACGACCGGGCGAAACTCCTTGCCGATGGTGACCTGCTTCTTGGCGGAGAGAGCGTCCTTGAAGTTCCCCGGCTTCTGGACGGACACCGCCGTGTTCTTCTTCCTCTTGTAGTAGAATATCTCGCCCGCCAGCGTCACCATCGCCAGGGCCAGCCCGAACAGCGTGGCGATGAACACGCCGCCTAGTGACTCCAGAGTGATGCCCTCACTATCGTCGGTATCAGGGCACAATCCTTTAGCTGAAGAGTTCCAGTATTTTCCTGACAACGTCTCGAAGTAGCGGTCTTTCTGCAGATCCAAAATTCTTCTGCTTATCTCAAGCGCTAAGTGGCTGCCCTGCTGAACTGCTATTGCGTATGGTTGTTCCGCAAACATTTCACCCACTTCCGTCAAGTTGCAGTTACGGGACACCTCGTAACGAATTTGTGCCGCATCGTGGATGAAAGCAAACTTGCCATCTTCTTCTGCAATCACCTTCTGGAAACCCACGGTGATATTCTCCACAGGCCCAGCTTGCGTGATAGCTTGTAGGATGTGGCCATACTGCTCTTTGATAGGGTAGTCCCACACTCGATACTTGGACTGGTCACTCGTGCTGTTCAAGGTGATGTCCTTCCACACATTGTAAAGCACATCTTCAGCATTCTTCATGTTCTTGAAGTACTCGTGAGTGTCAGAGTCTTTGACAACAGTGTAGTTAATCCTCGACTGTTTCGCTAACTGTTCTAGGGATGCAACGGGGGACTTCATCCTCTCGACGGTGAGGAAGGCGGCCAGGTTGGCGGTGAAGGTGGCCAGCATGAGCACCACGAACAGCCAGTAGGCGGCGACCAGAGTGCGCGCGGACAGCGCCTTGGGAGCCTCGCCGCCACCCTGCGGCGTGAACGACGTCAGCGCGAACCAGAAGCTCTCCTTCAGCGTGAACTCCCTGCATGGGTAAGGGTACATGGCCTTGTTGTTCTGGGCACTGTAAGGCGAGTACTTGTCCAGGAACCAGATCATGATGCCGGTAACTGTCAACGCTCCCACGATACTCAGCCACACTTCCAGTCGCAGGACCGTCATGAACTTGAACAGAGATGTTTTGCGCACCGGCTTTCTTATCACAATGGAGATTCCAGACTGGTCGAAGTAAGGAGCCACGAAGTCAATCACCTCCTCTCTCTCAGATGTCATGGTCAATGGTGCGATGATCATATCTGTCTCCCCGGTAGCCAGGTCGCCGACCATGCCGGTCCAGGAGCCGTCGGGACGGCGGCTGCCGAACGAGCCGTCCTTGGGCGGCACGATCTCGTAGTCGAACTGCAGCGTCTCGGCCAGGTCCCGGAGCAGGTCGATGCAGTAGCCGTCCCACGTCGGCCTGCCCTTCTCGTCCAGCAGCAGCGCGCCCGTCGTCTCGTCGCGCGCCATGTACGACCACGGGATGCTCTCGGTGGTGCCGATCCTGAAGAAGCGGCGGCCGGATTGCAAGGTGACGTTGTGCTCCAGCACCAGCTGGTTCTCCCTGCTGAAGGTGCCCTGGCGTTGCTGGTGGGTGGAGTTCGCCATCGTGATCTCCATGTCACCGCGCAGGTACAGCAGGCGCTCGCCCGGCGCGAACCCTATCGCCCGCCTCTCGCCCATAGTCGCCATCACCTGCGAGACGTTGCTGTAGAACTTGGTGTTGGCGGCGTCCGTGTCGGCGGCGTGCGCGGCGCAGTCCTGCACCGGCCGCGGACCCACCTCGACGCCGTGCTGGTCCAGGTGCGAGGCCAGGCGACCGAGGAACAGCACCACGTGGCGCAGGTAGGACGGCAGCACCTGGAGGTGGTTGTCGCAGCGGCAGCCGGCGGGCTGGTCCGCCAGGCGGCAGCACACGTCCGTCCTCATGGTGAAGAGCACGGCGCTGAGCTGGAGCGAGCTGCGGTCGAACTGCTGGTGGCTGAAGTCGGTGAACACCAGCAGCCAGCGCTCCTCGCGTGTCACCAGGTTCCCCTCCACCGCCTTGTCGTAGAGCTTGGTGATGTTCGTGGTGGTCGCGTAGACGACGTAGTAGCTGGGCGACGGCCTCATGGCGAGCAGGCGCTCCGTCGCGTTCCCCTCCAGGCCATCCAGCACGATCACTCTGATGATGGACCGGCCGATCATGTAGTACAGCGTCTGGTCCAGCTCTTGCTCGTTCTGGAAGATGAGCGCGGCGTCGGTGGCCTGGCGGCGGCCCAGGTAAGCGTCCACGGCGTCCACGAAGGCGCCCGGCGTCACGTCCGCGCGGAAGTAGGGCAGCGCCGCGCTGTCGGCGGTCGTCCTCACCTTGTCCCAGCCCGTCCACGTGATGTCCAGCACCGCGCTCACGCCCGCCGCCAGCTGCTTGCACACTTGCTCGAAGGACTCGTCCTCGTTCTCCCGGTCGACGGGGACGGGCACCTGGTCGAACCGCACGCCGTCGTACGCCTGCTCCGCCGACTTCAGGCCGGCGGCCACCATGTCCAGCACCTGCTCCTGGTTCTCCTCCGTCACCACCAGTATTTTAAAAGTTTTCTGTGCGGTCACCAGTTCCAGTGCCAAGGCGTAGACGACTACGTAAGGCAACATCGTGTAGACAGCGACAGCAGTCCTGACGCCTTTGACGGTCCGTCCAGATCCGAATAAGAGTGCCGTCTGTCTACACCCGCG

>PsicIR76b_partial mRNA

ACGCCGCGCGCGACCCTGGGCGAACGTACGGTGGGCGAGGGTAGGACAAGGACGCGACGCCAGTGGACGCTCGCTACTCGTCCAGGTCGGACGCCGTGTCGAGGATCCTGCGGTTCACCTGCCCCGGTTCATGCACGAGCATCTAAACGAATCCCTAGAATAATTTGTTTGCGTGTGAACTGTCACGAATACGTATAATTTCTCTTCACTGGTGGCGCACTTTCCCCGTCCTGGTCTTTGAAGGCCAAACGGGGAAGTTCTCAGCTGTAGAAAATGTAGGAATGAGGCTTCGCCTAGTTTCACCGTCTCGTCAGACAGATCCAGACTACAAGCGTGGCGTGAGAAACTCGAGATGCAGTCGCTGATACCGCTGCTGATGATGAACGTGTGTTCCAACTACATCGGCGAGAACACGACCAAAGTGCAGGACGCCGACGATGACGAGGAAGTGGTGGATCCAGGCTGCATTCTCCGCGACCCCCCACTGCTGACGCACATCCACCTCAAGATAGCCACCATCACGGACCACCCTCTCAGCTACGTGGTGGAGGAGAACGGACGGAAGGTGGGCAAGGGCGTGGTGTTCGACATCGTGGAGATCCTGCGCTCCAAGTTCGGCTTCACCTACGAGGTGGTGCAGCCGCGCGAAAACTCCATCGGCGACAACAACACCGGCCTGCTGAGCATGATACACCGTGGGGAAGCTGACATGGCGGCGTACTTCCTGCCCATCGTTTGGGAGAAGAACCACGGGGTGCGGTACTCGTTCAGCCTGGGCGACGTCGACTGGGTGGTGATGATGAGGCGGCCCACCGAGTCGGCCAACGGTTCCGGACTGTTCGCGCCCTTCGACACCACCGTGTGGCTGCTGATCCTCGTGTCGCTGATCCTCACCGGGCCCGTCATCTACCTCATCATCCTGGTGCGCGTGCGTCTGTGCAAGGGCAGCGAGCGGCTCACCAGGATCTACCCGCTCGACGCTTGCATCTGGTTCGTGTACGGCGCGCTCATGAAGCAGGGCTCTACCCTCAGCCCCATAACAGACTCGTCGCGGCTGCTGTTCGCCACGTGGTGGATCTTCATCACGATCCTGACGTCGTTCTACACGGCCAACCTGACCGCCTTCCTCACGCTCAGCCGCTTCACACTGCCCATCGACGACGCCTACGACATGGCCGTCTACCGCTACAGGTGGATGGCGCAGAAGGGCCTCACCATGCACGAGGTCGTCCGGTATGATCCTGGCTACTACTACCTGAACGACTCGTTGAAGGCGGGCCGAGGAGAGTTCTTAAGGGGAGACAACGCCAAGATGATGGCCATCGTGCAAAAGGAGAACAAGATGTTCCTGAGGGAGCGCAACGTGGTGGAGTACCTGATCCTGCGCGACTACGTGACCAAGACGCACAAGGGCATCGAGGAGACGAAGCGCTGCACCTTCGTGGCCACGCCCAAGGCGTTCATGGAGCGCTCCATCGCCTTCGCCTACCATCCCAAGTCCACCCTCTACAAGCTGTTCGACCCCGTGTTCATGGGTCTGGTGGAGCAAGGCATCGTGAAGCACCTGCTGCGGCGAGGCCTGCCCAAGAACGAGATCTGCCCGCTGAACCTGGGCAGCAAGGAGCGCCAGCTGCGCAACTCCGACCTCTTCATGACCTACCTGATCGTCATGTCGGGCTACGCCATCGCCATCTCCGTCTTCGTCGGCGAGCTGATCGTGCGCGCCGCCAAGAAGTTCAACGACAGCCGCCTCATCAACACTCACGACAACGGCTACGTGCCCAGCAAGTCGCACATGTTCCCGCCGCCCTACTCCACCGTGCTGATTGGCGTGGACGCGGAGGGCGGCAAGAAGCAGAGCATCAACGGCCGCGACTACCTGGTGGTGAACGCCAAGGACGGCGACCGTCGCCTCATCCCCATGAGGACGCCGTCCGCCTTCCTCTTCCAGTACTCCGCCTAGGCGTGCGAGCGGGCGCCGTCTTGCAGCAGCAGCTCCGGCTGCATCATTCCGTCCTTCGCCGCCCGCGTCTGCTGCCCCTGACAGTCGAGGTCCCGTTCTACTGCCGCCCACGTTCACAAGCGCCACTCAAACCAAAGGAGCATTATGGATGAGCAGCAACTGCGTGAAACAAGGTGCCTAGGAGCATGGCTGAAGCGCAGCTGCTCGGCACGAACCTGCGATATATTGCTTGCACAGATTATCTCAACTTTATGAGTGCATAGCAATCTTACAGCATGCTGCCATGATTTTTTATTTTTGCAGAAGTACACTAGATCTGCGAGTGAATCGCAGCTGCTCAACACAAGCTTGTTTGGTTATCTAATTTACATAAATTGCAAATGGACCGCAGTTTTTTTTTAGCATAGGCAGTACATAGGGAACATGGGACACAAATAGCTTGGAATTTTTGTGAAATTTTTTGGCATTAAACAAGGATTTTGTGTTTCTTGAGAAACCTAGCGTTATGGGCGAATTGCATTAGTTTAGCATAAACCTAGAAGACGTGGATTCAGTAAACTGACAATACAGTTAAAAATAACTGTCCAGTATAATCTTGGAATATTTGTGTGTCCAAACTGTTTGGCATTTCGTGTAAATCACCGTTCACGAAAGGGTCGGTCCTGCTCGAAAACACATTCGCGGGTAACTGAAGCCAACATACGCTGGCCGACGGAATGCGGTCATGTCCTGATATGTCACTAGCCGTGCGCCCGTCGCGGAGAAGCGAACAATCGCTGCTGTCTTGTCAAGGACACAAAACCGACAGGAGTATCGGTACTATGGACTGCCAGAAATTGGAAACCACTAACCAACCGGATGTCTGGTCTTCAGCTAGACTCTCTCTGTCAGAAAATGTATCCAGGTGTTCAGACGTTGAACCGTGGTATTTTTCACTCCTTTAAACCATGCCGTTAAACAGGTTTATGCAGAAAGAACTTAAACACGCGGTGGATGCTATCAACAACGGGAGTATAGGCTATGCTTACCCCACCCAGATCAATAAACTGAACTTGTTTGTACATGTCTGCATTTATTTCCGTTTGCAATTTTGATCATTTTAACTTTATTCCGCACATCTCTTTAATAACGTCCGTACAAAACGGGATATATGCTCAATCATATTCACTTGCACGGAAAGCATACGGAAACACGGTAGTCTGGGGCGAGTAGGGTATAGTTGTAAGTAGCTTATTTGATGTTTGCAAAGTTCAAAATAAACACAAGGATTGCAAAATAAAATGTAGATTTTCATTTTAAGTTCTTACCATGGCAATAAACTCGTCACATGCAGTCAAATTTGACTGCAGAGAATGGTCCTCGTTAACCTACATTCCGCTACGGTTTTTAGTCGACTCTGCGACTTAACTGATGTGTTGAACGCTTCAAAGTTCACAGGCACATTGGGACACACGTTCGTAAAGTTGAAACAGGAAACACTTTTCAGGATAATACAACTGAACGTCGACACTTCGCAAACTAACATCAGTCATAAAGATGCCTTTGAAACGGAACAACAGATTTTGTTAGTACATTTTACAATGTTATTAATTATGCATTCATCCCTGAGAACACAGATCAAAACCAAGTACAATTAGCAACAAAGTGTGCTCACTTGAATGTCGCACCTTCGCCCCTGTGTCCTGCACCATTTGAAATTTGTAATAACACAATTTGAGTGTTTTAAACTGAGAATCACGTCATTCCGCACGTTTTTATGCTGTCTTTTCTTACTATGGTAAAAGACGATAGATACTAAGATCATATAATGGAGCTAATAATTTGAAATATAGACTAAACGTTTGTTGTGTAGCAATGAATAATTGAAAAATATGAGTTATTATGATGGTGTATCAGTCTTTTCACCGATAAACTTCATGTAGAAGTATTTGAGCTCAATAAAGCAGTAGTGATATAGCATAGGGTTTTTAATTTACTGCAATAAAGTTTTCTTTATAAGGAATAAAATACTTCGTTATCACTAAAGCGTGTCATTTGGAAATAAAGCTTGGTTATTAATGTGTGGCAGTGTTTTACTGCTGGTGTCGCTTAATACTGACTAACTGGATTTGTTCACATCGACACCAGTTGTTAATGTTTGACTGTAAAACGATTTGCTATTAAGACTTCTACTGCTAACAACATGCAAGCAAAAATAAAATGCAATAAGGTAGAAAGTAATATTTTCTGACTGCGTGATGGCTGTGGGCTTGACCTGAACCATGAGTTGTACAACCACTTAGGACCCTCAGTGTCCAAATACGTATATTGGTTATACTCCACCTGTAGGTCGCATTCTGAGTACACAAATATTTGGGCTTAGATGTTGGGGAAAACTTTAGAGAGGTTTGGCAGGCAGCTGTGGCTGGGAAACATGAGTGAAAATTTAATTTAATAAAAAAACCGCGTTTCTATGGAAATTTTAAATAATACTAATTATAACACAAACTCAATTTTTTTCAGAAGATGTTGAACATGGGAATTTTCTCTGATGCATATGTTGAACTGCTTGGTAAGTGATAGTCAAACACTAAGTCCACACAATAGCATTCTGCAGAACCAAGAAAACATTTGACCTTTGGATTTTTTCCACCTCTGTAAGCACGTATTCTAAGGAAGCCGCTTATCCTCCCCCATATTTTACAAACCACAACCGCAAAACAACCAATATCAAGCAGAAACCCCGCAGAAAACTAACCCACGCATGAGCAGGAACAGGTGTATATGCGTAGAAGAGGTCCATACCTTTTGGCTTAGTCATTTAGTGTACTGTGATTAAACGGTGGAAAAATTTGTAAAGTTCAATTGAACTCTGGCTAATAGAATAGGTGACATTATGAAAATGGACAATGAGCGAGCCAATTATACAGTAGCTGGAAGTGCAAATGATTTGACCCTACTTCCTTTGCGGATTTATTGATCGAGCACTTGCCAGACAAATGAATTTAATATACTGAAATGTGCTACGGAAAAGGGGCGGGGGGAACTTAAAGACAGCCAACACGAAATGGATTTGTGAGTATTTTTCCAGTTTCTGGTTTTGGAGTGTGGCTGTCTTACGAAACGCAGATATCCGTAAACCTGGCGCAATTTGAAAACAATTCAACAGTTTTTCGTCACTGGCAGACAGAGTTATTATTTAGCTCTTCCTCACTTGTTCCCTCGTCGCAGGCCATTTTTCATTCAAAATAAAAGGTGGAATCAAATTCAGAACAACATTCACCTAGGGAATAGCATGAACATAAAATTTCACCAATATTCATACCTTTAGAACAAATTTACTGAAACTAAGTGGTTGTGCAGACATTTTGTACTTTATCTTCATAAGAGTGTGCGCAGGGTTTTTCAAATCACATTAGAATGTATCTCACTTTTCTCAGAAATTAAAATCGGTAGTGAATATAATAATTTCTTAAGTTCTTGTTTGTATGACTGCATTCCAATTATTTCAGATAGCATTTTATTTGATTTTTTGGAGTGACGAAGAACAATATTTGTGTAGTAAAATTTACTGATCTTATTATTGCCCATGTTGAAATTACAATATTATAATTAAGATACTGCTGGCATATTTCGTGTTTTAGAGGTGCGTTTGATTTTAAAAATACTTGGAGGATTTTTATTCTTGCA

>PsicGluR636

GTGAAGCTGACGCCGGGCGGCCTGAAGCAGGTCGGGAACTGGCACTCGACCGAGGGCCTCAACATCTCGGCCACCATCGAGGAGGAGCCCCTGGTCACCGACGACAACACGCTGCGGAACAAAACCCTCATCGTGCTGCTGTCGCTCACGAAGCCGTACGTGATGACGAAGATCTCGAAAGATGCGCTGTTTGGGAACGAGAGGTACGAGGGCTTCTGCATCGACCTGATCAAGGAGCTGGCCGGCATGCTGCACTTCAACTACACGTTCGTGCTGCACTACGACAGCAACTATGGCGGCATCAAGAACGGCGAGTGGACTGGCATGATCCGCCGCATCATGGACGGCGAAGCCGACCTCGCCACCACGGACCTCACCATCACAGCGGAGCGGGAGTCCGGCGCCGACTTCACCATGCCCTTCATGAACCTCGGTATTAGCATCCTTTACAAGAAACCTCAGAGGGCTGCCCCGGAGCTGTTTTCCTTCATGGCTCCATTTTCCATGGGTGTCTGGGGGTGCATGCTTTCTGTCTATGTCGGTACCTCTGTTCTTCTGTTCATCATGGGCAGGATCTGCCCGTACGAGTGGGCGAACCCGTACCCGTGCATCGAGGAGCCGGACGAGCTGGAGAACCAGTTCAGTCTGGGCAACAGCTTGTGGTTCACCATCGGCAGCCTTCTGCAACAGGGCTCAGAAATCGCGCCAATGGCCGTGTCTACGCGCATGGTGGCCGGCATGTGGTGGTTCTTCACGCTCATCATGGTGTCTTCGTACACCGCCAACCTGGCCGCCTTCCTCACCGTCGAGACCACCTTCTCGCCCTTCACCGACGTCAAGTCGCTCGCCGAGCAGAAGACCGTCAAGTATGGTGCCAAGGTGAACGGCGCCACATTCGCCTTCTTCAAGGAATCGACCAATCCAGTTTACCAGAAAATGTACAAGTACATGGAGGAGAACAAGGCGGACGTGATGTTGATGACGAATGAAGACGGCGAAGAGCGAGTGAAGAGCGAAGGCGAAGACTACGCATTCCTGATGGAGTCCTCCTCCATAGACTACACTGTCGAGAGGAACTGCGAGCTGACGAGAGTCGGGGGCCTGCTGGACAACAAAACACTTATTATTCCTGTCGAACACAGTTGCAGCCATGCACAAACACCAGTTTCTATGCTGGAGGTGGTGGAACTACACAGAAAAAATAAGGCGGCATTACACTTTGTCGTTTTTACACCAGATTTTATACACGTAGCTCAGGCGAATGTTATGAAATGATCTGAAAAAAAAATGCAACAGGGAAAATAATACATAATTAGAATAATGGAACTGTAAATGTCAATATGACGAAAAAATATTATTTGAATGTTATGTTACTGCCACCAATAATATTTTTATGGTGGAAAATACCCTAAGATACCTGTAGGATTTAACCTAGTATAGTT

>PsicGluR7966

AAAGACATATTGCTGGAGCTGATGGAGGTGTCACAGGCCAAGCTGAGGAAGATCGGTAACTGGACTGTTTATGGAGGCATAACACAGGAGAAGGACTACAGTGAGCAGGTGTCGCTGGAAGCCAGGCAGATGATGCAAAACAAGACATTCATCGTCGCTTCCAAGATAGGCCCACCGTACCTGGGCTGGAAGAACCGAAGCGCTGTGGGGAATGAGCGTTTTGAAGGTTTTTCTCTGGACCTCATAGATGCGGTAGCCAAATTTAATAATTTCAAATCATACGAGTTTGTAATTGTGGCAGATAACCAACATGGGACACAGGACGCAGAAACTGGCCAGTGGAATGGCATCATGGGAGAAGTGATCGGCAGGAGAGCTGACATGGGCATCTGTGACCTGACGATCACCTACAGTCGTGGATCAGCTGTTGATTTCTCAGCACCTTTCATGAACCTAGGAGTCTCTTTTCTCCTCACTAAGCCTACCAAGGATCCTCCAGAGATGTTTTCATTCTTCTTTCCTTTTTCTTTTGATGTTTGGATTTACATGGCGACCGCGTTTCTCGGAGTTACTCTCATTCTTTTCGTGTTGTGCAGGATTACACCTCACGAGTGGGATAACCCGAATCCAATGGATGATGACCCAGAAGAGCTAGAGAACACATTCAACTTAATGAACTGCTTATGGTTCTCCATAGGATCACTTATGGCACAAGGGTGTGACCTTCTACCCAGGGCAGTTTCTACTAGGATGGCAGCTGGCATGTGGTGGTTCTTTACCTTGATCATCACTTCCTCATACACTGCAAATCTAGCAGCTTTCCTCACCAACAACAGGATTGATGACAATATCCAAAGTGCAGAAGATCTCGCAGACCAGACGTCGGTTAAATTTGGTTCTCTGAGAGGTGGATCTACAGCTCAGTTCTTTGCATCATCAAATTACACTGTGTACCAAAGAATATCAACGATGATGAAGCAAGCAAAGCCTGATGTGTTTACTAGCAGCAACCAGGAGGGTGTAGACCGGGTGCTGAAGGAAAAGGGAAAGTATGCTTTCTTCATGGAATCTACTAGCATAGAGTACGAGACAGAACGTCACTGTGAACTGACTCAGATAAATGGTCTTCTGGATAGCAAAGGCTATGGGATCGCCCTACCTTTCAATTCACCGTACAGAACATTTGTGAGTGAAGCTGTACTTAAACTCTCAGAAACAGGAAAGATAAAAGATATTAAAGACAAGTGGTGGCTGGTGAAAGATGGGACAGGATGCAGCGAGATAGAGACGGAAAAGGTGAATAATGATGAGCTTAAGATGGCCAATGTTGGAGGTGTCTTCTTGGTGCTCATAGTGGGCTGCTTTGCTGCCTTCTTCGTTTCTATCCTAGAGATGCTGTGGAACTGCAGGAAGATCGCTGTTGAGGAAAAGATAACACCTTGTGAAGCCTTGATCTCCGAGCTCAAGTTTGCTGTTAATCTGTCGCAGACAACCAAACCAAACAGAAAGAAGAAAAGCAGAAAATCAGGAGGTTCTTCAAGTTCCTCAGTGTCGGGTTCTGTACTTGTTGATGCCAGCCATGATTAGTAACATTGTGTGATGAATAATTACAAGTACCATACATCATTAACTCTGCAGAATGTCACAATATTGTTACATGTGCACCATACCAACTATTTTACAACCAACAAATTATAAGATGCAATTCTGAAGAAAGCTTGTAAAGTAATGTAAAATAAC

**Additional contigs**

>Lsig106170_putative variant iGluR_partial mRNA

CCAACATCTGCCCTTTGATAGCTTACATCTCCCATCATTCCTATAAGAGTGCCATTTTTTAATTCACTTCCCCACTTTAGACCATCACTTTGCGTTGTGTATTTGGGTTTAAAGTTGAGTTGTTTTCCGAGTGTTTCAATCAAATTAAATTCAACTCCTGCTGTAGGTTTGTCTGTGTTTCCCCATTTCATATACGGTAAATGATTGAGTGTACTTATATATACTTCATGCCCATTTAAGTTCGATGTCTTGCTTGGAAAGTACTGTGTATTTCTAACAGGGTTTTGGCTTTCGAAAGTAACATCTTCATTCTCAACTGTACAATGATCAACGACATAAAGAGAGCTCTCATCACTCTTTGGAAATGGCTGATAGGAAAGCAATTGAAAAACAAATTTCTGATCTCCATCACAGGAAGTCGCAGTGACAACTACACTGTTGACAATTTTCCAAAATTTCAGTTCTTGCAGTTCTTCTTTCCCGCTACTATTTGAAGTTAAACAGACAACAAACTTC

>TdomIR25a_2_partial mRNA

TATACATTCTTGTCCAGACTTATTTCTGGAGTAGTTGTTAGTGAGAAAAACGCCAATTAACCACGCGTTACAAATTGTCTTTGGCTTCATGTTTTTAGTTCTGACAGTTACCACCAGGGAATAACTGATATTTTGGTGTCGTTAATTTGGAAGACCACACCATGTGGTCCAGTTTTCTCTTTCTGCTGGTGCTCCACGTTGCGTGGGTCCATAGTCAACAAAACTTAAATATCCTATTTGTCTACGATGAGAAGAACACAATTGCCGAGAAAGCAGTAGGAGTTGCTCAGGACTATCTACGAAGACAAAACAAATATGGTGTCACAATTAATAATTTCGATACAGTGGTTGTCACCAACAGCTCCGACGCCAGAGGTCTACTTGATAAATTGTGTAAGGCGTATAATGACTCCATTGCTGCAGGAAAACCACCAGATTTAGTACTGGATACCACAATGACTGGTATAACATCTGAAACTGTGAAATCGTTCACAAATGCGTTAGCTCTACCTACCATCAGTACTTCCTACGGCCAGGAAGGAGATTTGAGACAATGGCGTGATTTAGATTCCGAAAAACAGAAATATCTAATTCAGATAATGCCTCCAGGGGATTTGATGCCACAAGTTGTCAGAAGTATTGTAGCAGCTCAAAATATCAGTAACGCTGCAATTCTTTTTGATGATTCATTCGTGATGGACCACAAGTATAAATCCCTTCTGCAGAACATGCCAACACGACATGTCATTACCATGGTAGATAAAGACCCGAAAAAGCAGTTGACACGACTTCGTGATGTAGACATTGTAAATTTCTTTGTGCTGGGCAACGCACCGACTATTAAAAAGGTCCTAGAAGCTGGTGCTTATAAAGGATATTTTGATAGGAAATTTGCTTGGTACGCTATATCCAAGGATGAGACAGCTCTTGAATGTAATACTTGCCAAAATGCCAGCGTAGGAGTCCTGAAGCCAGTGATTAATCCTGATTATAAAAATAATTTTGGTGAACTTAAATCAGAATACAAACTGGACATGAAACCAGAGATTGACGCGGCATTCTATTTTGATGTGGCCGTTAGATCATTCATAGCTGTCAAAACAATGAAAGACCGAGGCGAATGGCCCACTGACATGAAATACATTACTTGTGATGAATACGATGATAACACAGCCCCTCAACGTACTAATATAGATCTGAAGAAGGCCTTTAAAGAGGTGACTGATCCTAAACCATCCTATGGAAATCTGAAAATTGACAGAAATGGATTCAGTTTCATGGAATTCACAATGCCATTGCGAAAGAAACGTTTTGTGGGTGGGCAAGGACAAACTGCTGCTGATTTGGCCGTTTGGGAAGCCAGTATAGAAGGACCAATCAAAATCAAGAA

>TdomIR8a_2_partial mRNA

ACAGATGATAGCGAGGGTATTACCTTGGAAAGCTTGGGTGGCGTGTTCATAGCTACTTTGTTTGGATTGGCCCTGGCTATGATCACATTAGCAGGAGAGGTATTTTATCATAAAAGGAAACAGAAGAATACAGTAAAGGCGATGAATAATAAGCCCGTGACCATTGGCAATGAGTTCAAGCCGGCTGATAAGATGCCCCGTGTATCCTACATATCGGTATTCCCACGCAACTGATATTCAAGTAACACCCAGAAGGGTGATGTTCCTGCTGAGCAATGGGGATTTTATGCGTAAGAAGCAAAAGCGCTATTCGAAATTTATTACTAATACATGACCAGAAG

>TdomIR8a_partial mRNA

CTCTTGTCGAATTTGTCATAGTTAAAATCAGTGAACACAAGATTCCATCGAGAGTCACGTGTTACAAGTTGACCATTTACAGCCGTATTGAATAACTCCTTCATATTGTCAGTGTCAGCAAATATGACAAAGTATGATGGTGTGGGTCTCATTTTCCTTAAACCTAAAGTTGTGTTATCTTGTAAACGATTCAGCACAATAACTCTTAATATGGAATGGCCAATCAAGTAATATAAAGTCTGGTCCAATTCTGCTTCATTTTGGAAAATAAGTGCAGCGTCTGTGCCATTTCGTTTCTTCAGGTAAGCTTCCATGGCTTCTACGAATGGCCGAACAGTGGCATCCAACCGGATATAAGGAATCCCTGCGTCGTCTGCCAATTTCATACTTCTGTACCAACCAGTCCATGTGAAATCCAATATGACAGACACACCTTTTTTAGTTTCCTGACAAAGATTCTCGTAGCCCTCATCTTCTCCTTCTCGGCTAACGGGTATCGTTACTTGGTCAAGTTTCAGGTCATGAGACTCTTCAGCTTTCTTCACGCTCTGTCCCAAAATGTTGGCAATTGCTTGTTGATGGCTCTCGGTAACAGCAACAAATCTAATTGGAGACTGACATCTAACGCAGCAAACCAGTAACAATCCAATAAGGATCTGAGGATATTTTCCCGAGGCCATTGTAATGAAATATTCACTTAATAATTGCTTCAAGACATAGCCTTTTGTCTACAAACTGGCTTGCTGATACACTTCATACACCCAATGATTCAAATTGGGTGGCGCCACCTTGAATCACAATCCAGAACTATAACTTTGTAACGCCGCTACAGTC

>TdomIR76b_2_partial mRNA

ATAATTTATTTTTGTATTCTCAATATTACAGTATTATAATATATTTATTTGAATTGAAAAAGGTTTGAACTTTATTAACATAATATAGGTGGACATATCACAGTACAAAGATTACGTTAGAAGATAATTACTGGTTGCCATATCTCTGTTTAGTAAATTGCATTCTTTTACTAGGTTTAATTTTTGTTTGTTTCGGTTTTTCTCTATTGAGAGTCGTAATTCTTCTTAAATGCAGGTCTTCATCTTTATGACTTCTTCTGTTTGATATTATTTGATGCAAAATAATGCTGTAATGAAGAATCAGTTCCAGAAGAAATATGATCACAGCGGCAAGCGCACCACATCCAAAAAGATATAACGCCGGAATAACGTCTTGCAAATCGATACTGTTAAATACAGCAGCATCCCCATTTAAACATAAGGGCTTCAGGATAAACCATCGTTTGATTTCGCGATCAATGAGGC

>TdomIR93a_partial mRNA

CTTGAATCTTTAAGTAAGCCCCATGTAATGGTTCCCTTTCGTTTCAACAGATCATCCACTGACTCCACAGGATTCTCTATTTTGGGGAACGTAAGAAAGGCGACTAAATTCCCACAGTACGTAGTAACAATAACTAAAACAACCAACCACCATGTGCCAATTACGAGACGACCGCTGTCTGTTTCTGGTAGATGCATTCCACCTTGTTGTAATAAAGCACCATAAACATACCAGTAGCAATTGGTGATTCGGAAGAGACCTCCTGAAGTCTTCTTACACTGAAATCTGTAATTTAGACTCCGGCGATGGACGTAATTTAAAATAGGACCCATCAGAATTACTGATACAGCAATGCACAACCAAGAATCAGTAGTAAATGGGTACATGAAAAGAAGTACTCGACTTAG

>Tdom_21471_putative variant iGluR_partial mRNA

TACCAGAGAAAACTTTAATTATAGTATGCCACTAAGTTACAAGATAGTAAAACTTTTATTACAATTCCTCAAAAGATGAATAAAGTTTATATACGCATTTTAAAGGAAGTTTTGTATGACATAGTCAATCAATAAACGGTTTGACAATATGTCTATCTGTACACGATCGGATATGCCAGTGAGAAAGTTCACCTGCAAGGGTAAATATGGATAAGATTATGCCTAACAACAGTAAAAGCAAGAGTGGTATTACATCTAGTAAGCTGACACTGGTAAGCTCCTCTGTAACCGAATCTTCAGTACGTAGAAGTCCTCGAGTAATTATCAGCCTATACAGAACGCCATCTTCCTTCATTCGTTTCAAATAATAGTTTATAATCTTTCTGTAAGGAAATCTGAGCTTCATGGGCAAGGACACAGTGGTCTTGAAGTAATCTGTTGACGTATCAACAATAGGACAAGAAGCACTTGGCAGAGTCATCGCAGCAGAAAGAGTAGACATAATAAAAGCAAATCGTTCGTTACAGACTCTGTTGAGGCCTTCTGGTATTGTTAATGGTTGTTCGGTCTCATGTAACTTACCCATCACTTTATTAAACACAGCATCATTTGTACTCT

> Tdom_34097_putative variant iGluR_partial mRNA

CCTCTAATTAGATCACCCGTAACACCCGTAATCGTACCATTTGGAAGGGTTTTCCCCCATTTTTCCTCATCTCGTTCATGAACAATCTTCCAGGTAAAGTTAAACGTCTTGGCAAGTTCTGATAAAAACCTGGTTTCTATACCATCTTGCAAGATCCTAGATTTTCCGCTGCCTTTGTAAATTACGAACGGTGGACAATGAAATCCACTTACTCTAATAGGTCGGCCTCGAAGATCGGTAGGTTCACGTGGGAATAACGATCCGTAAGTTACCAATCCTGTCTCCTTTCTCCAAAATCCAACTGGTGTGTCTTGCTTGGTAGGTAAGGATACTGTGAAAGTTGTCCCTGTTGAATTAATTTTTCGAGACACTGTGATTACTTCTGATGTTTCATAAAAGGCAGGACATCCTACAGAAACATCTTTGTTTTCAATATCTTCATCACTGAATGCTACCACTCGATTGCCAGGTGTCAGGAAAAAATTTCTACCGTATTCCATGGCTAAAGTACGAGGATTTTTCAGAGCAAGAATGTAGCCATTGCAATTTTCAGATTGTCCTTCAGTAATTAAATCAATCCATTCTTTGGGGCTACCATCTGATCTGGGAGTCACCTCTATGTTGATAACGGGAACGGAAACGGACAAGTAAGTCGCTGCCATTATATCACTTGACATTGTTCCTTTATCTGTCAACAAGTGAACACAACGATAAGACCGGAACATGTCTTTGAGTATTTCTTTTGCTACCGGTTTGAAATCAGATGAATCTTGATTCCACATGAG

>Tdom_39421_putative variant iGluR_partial mRNA

ATACGTATGACACTAAGTATAATAAAACCTTTTATGGACTATTATCTGATGACAACTACCATGTTGGAATTCCTTCGAGTTACATTCGCTTTGATGTTCTTGAGAATTTTGATGGATCTGTACCATTCCTTGAGACAAGTTTAACTTTCCTTACGCCTAAACCTGGTAAGAAGAGTTCGATACTATCAATATTTGAACCCTTCAATGAAGTACTTTGGATGGCCTTAGGTTGTCTTAT

>Tdom_ 42947_putative variant iGluR_partial mRNA

TGGTAGCAACGCTACATTTCTCCTCTTCTGTTAGAGTTTCTTGTAAGTAGGTGATTGCTTCATTTGTATCTGTGATGAATGCCATTTGACCCTTCACTACAGACTGAAGACCTTCTTCTTTTTCAAAGAAAACATTACTCAGGTGAGGTTCCATCTTCTTTCTATATAACTCTCTTGCGAATTCATTAGTTTCATTCTTGTTGACAGAAAGCTCCAAC

>Tdom_64008_putative variant iGluR_partial mRNA

TCTTCGCTGTGATTTTCGACATCATTATGGATGAGTAAGAATTATAGATCAAATATCCAAAGAGTACAGCAGTTATATGCACGATGCGACCAGACCAGAAAGTAGCATGTATAGGTGAACCTTGAGAGCACAAGTTACCCAAAACAATAAGATGTGCTTCTGAGAGATCGACTACTCTGGCTTTGTTTGATCCTATTTTGTAATTCAGTGTACTAATACAGTAGACAGCAGTTGATAATATAATCCAAGTAAACAAA

>Tdom_71755_putative variant iGluR_partial mRNA

GTTGGAGCTTTCTGTCAACAAGGAACAGCTCGTGACCCGGTGACAGCTGCAGCCAGAATATTGTTCATTGCCCTTTTTGTGCTAGCAGTTCTTCTGTACACTGCATATTCAGGGAACGTTGTATCTCTTCTGTCAGCTACTGGATCTGTCACAAACACACAACAGCAAATTATTAATGACAAGATGATGGTTGGAGCTGAGAAGACTGAGTATTTCCTTAGCTACTTTAAGGTATGTTGATTAAAACAATGTTTGTGTTGCTGCGTTTAATGTCGGTTT

>Tdom_82603_putative variant iGluR_partial mRNA

GTTGGCAATGAGGATGATGTACCCGTTGCATCCGTCCTTCCTTACTTGTTCAATGGCTGCCAACGTCGTGTAGTGAGGGTTGATGAGGTCTTCATTATCCTTTACAGCCACTTTGAAGAACGGCCGGTACTCCAGGGTGGCAAAGAAACTGGCATGAAGCAGGCTGCTATAAATCGAGTCGGTTATGATGCAGAGGCAACTTAACTTATCCATGAACCGAAGAATTTGTGTGAGAAGTTGTTCTAGAAGGCGATGTCTCATGGATTCATTGACAGC

>Tdom_86455_putative variant iGluR_partial mRNA

CCATGCATGTTTAAAATTTTATCAGGAAATAGCTTACCATTGGAATGATAGTAACCGTTCTTGCAATACCCCATCAGAATTTTTTCAACATATTCTTCACAAAAATTTACGGAATAAGGGAAGTACGTGTACCACAAAGTTTCGTTATTGGTACTGATTGCAATAATAGTGTTAACTAGTCGTAATTTCATCATAAGTTCTAATATTATTCTGATTTTAGTTTTGTCAATTGTTTCATCAGTAAAGATAATTATAATTTTGGAGTGAACATCTATTACTTTAAACTTATTGAAAAAGGACACAAATTGTTGAAGGGAGTCTGCTAAAATCAAGATATTATCAGGAGAATTCGTGTCCAT

>Tdom_88427_putative variant iGluR_partial mRNA

GTTGAAATACTGATAGTAGAAACGATTTTCTTTGTGGCTTTGGCGTAAGGAAGCTTATACTTGTCTGTAGAAAAGGTATTGAAACGTCATAATATTCAAGCGCTGGTGCTAATATATATCCTGAAGGTATTCCTGCTTGAAATTTGGTGTCAAGCAATAACGAATAAAATGTTTCTCCAGATGATAAATCTTCAGAGGTACCCCAATCCGGGTTGGTAAAATGAAGATTCCAAGACAGATTGTAAT

>Tdom_89771_putative variant iGluR_partial mRNA

CTTCATAATCCTCAAGTCCAAACCTTCATGTATAGTTTCATTTAGTAGAATAGTATAAGGCTCATATTCCATAAAACATGCAAGGATTATATGTCTTTGAAGATTAGAAAGTTTATCAACAAATAAATCAACGTTATTTATAAAACTACCTGAGTCCCATACGTCAATCCTGAATGGATTTCCAGGATTTAAGTAATCATCAACCGTAAAAGTTGAAAATTGTACTTTATACGTTAAGTTCTTCGTATCAGTTACGTTGGTTGCTGGTGTGGCAATTATAG

>Tdom_93549_putative variant iGluR_partial mRNA

TCCCTATAATCCTGATAAGTTTTCCCTCAATTCCACTTACTCTTGTGCTGTTGTCGTCATTTTGCTTTAAAATAACTTGAGGAGCAATTTCAGCCGTACCTACAACTAACGGACAACCACCCATATTTCTAGTTTTATCAGGAAAAATAGTAGTCCTCTCTCTATAAATTTCTTGGCATGTGTTTAACAGTTGTACACGAATAGCTTTGCCACACTG

>Tdom_94268_putative variant iGluR_partial mRNA

AAACCCCATTTCATACCATCCTTAGGTGGTTTAGCTACAACAGTAAAGTTCATTCTATTTGATAGAGATGTTATTATTTTACCCTCAAAGCCTCCTAATTCTTTTGTCCTTTTATCATTTATTTGTGTGTAAGGAAGAAAATCAAATGTACTCAATCGCAATGGGCATTTGTTTAAGTTAGTAAATTCTGATTCCAATTGCATGCTCTCTTTAAAGTTTCTGGTTTTTGTACATTCTCTTAGTATAACCAAATCAGTTACATACCCACAAG

>Tdom_96733_putative variant iGluR_partial mRNA

CGCTTCTGTTAAAACAAACCAAGAACTAAAATCCTTCCACTTCTTTAGAATATTTTTCATAACGTAATCAGAAGGATCACTGAAATATCTTCGAGATACTGCAAAACCACCAAACGGAATACCTGACTGTAGAAGATCATTCATTGTATTAATCGGCGGGGGATGTTTTGGTTTGGTCACAAATCCAAAAAGAGATGCGTGGTAGCAGTAATTTAGAATGAAAGAACCAAGCACCCACATTAGAAATATTAGTTTCAATAGAGGTATACG

>Psic_11602_partial mRNA

CAAAACTGTTTATGTCGGAGTAATACTGCGGAACAGTAAGGTAGCTGGTAAATGATCCAGAGTATGCATTATTTAAGACAATATTGAACAACATTACAGATGCTAAGTATACTCTTAGGCTGATTGCTGTAACAGGTTGTGACATTCCAGTAAAAAGTAATCGAAATGTTTCCAAAAAAGAAAATAAAGCAAGTGTGTTTCCTCTTCTGTAACGCGAATATACCTTCCCAAACACAACAAGCAAAACGTAGACACCTAAATAACAAATCCAAACCTCATAATCAAACGGCAATATGGCACACATCCATTTGGGAATTTGCTGTGCTTTCGGAACGAGCAAAACAAGTTTGTCATGGAGGAATGGG

>Psic_12275_putative variant iGluR_partial mRNA

GGTTTCGTGGGCACTTTTGCAGCAATTGAAGAAGCCTGCAGAAATCTGCCTGTGGTGCTGCGCCTCATCTCCCAGTCTACCTCCCTCTTCAGCTTCAGCACCAGTCCTGACTGCACAGCACGGAGCAGCACCTGGTTCAACATGTCGCTGTGTGGGGCACGTCTTGGGAATGCTATGGCCACGTGGAATGGCACAAAGCACTGCTTCGCAATGTGCAAGAATGCCCGTTTGCTGGATTCTCCATGTGTAAAGTTAGCTCTGAGTATGAAGTCAAGTTTGTCTCGAGAGCCAAAAACAGCATAGCGAGAGTTTCGCCATTTGGTCAAGTTTCTTAGGCCGTCCTCGATGGCAACAAGGTGCTCTACTTTCTTCAGAACTTTGGTTGCAATAGGGTCATGCGAATTGTTGAAATAATCCTGCCATCCTTCCCCATCTAACATGGCTACTTTGAAACCACCTTCATAAACTTGTCTGGCAGAATCAATCAGTTGTGGATACAGTGGCAATGTGACAAATGCAATAATGGAACCTGTATAGCAGGCTGTCACAATAATGCTGAACATCCAGTATGTACCTGGGCAGTGCAGTAGAGGTCCTCGTGATGAATGTGGCGCAATCTATTGAGTGTCCTGCGGAAAGCGTAAGGTGTCTCATTGTTGTACTGGAGGTGTAGAAGCCAGCCACACCTATGTGTGCTCTGTGGGACATCAGTTCCTGCACGACACCACTTGCCTGCCCGCTCCTCCTGTCTGTGGCTGGGAAGTTTCTAAACTTTAGTTTAAAGTTGAGAGTTTGGGAGAGAAGCCTCAGTAAGCGAACTTCCAGTCCATCCCATGTGTTTCCACTAGCATCATCATCTGAAACATTCCGGTGTATGACATAGTGAGG

>Psic_13734_putative variant iGluR_partial mRNA

ACTGATGAGATCAAGGAATAAGGCTGGTGAGACATCATCTTCCATAGTTTTTTGTATATTCCTGTGCCATTCTCTAGTATCCCATAGGAAGAACTGTGGCTCTCAACCAGCAACTGGTATCCATCTTCCCTCATAGCCTGCTCTAGTTTTGCCAAAGTGTCAATTGGCGTTTCTCTCCCAGGGCAGGCCAGCAGAGATGTCAGGTTGGCAGAGTACATATCTCCAAT

>Psic_35409_putative variant iGluR_partial mRNA

GGGATATCTGTTGGAGTTTTGTGCCAACCTTGCTGACACGTGGTCCCCAGAGAGCTGAGGAATATCTCGCTCCAGTCCCTGGAGTGTGGCTCCAAGCTACTTGACCTGCCCTTTATCCACTGCACCTTGAACATGACCACCGTCAAGACAACCACTGTGGCAATGTACGTAGCCCAGACACCTTCGCTGAATGGCATCACGTATATGTTGGACATCGATGACAAGGATGGCTGTCTGAAGATGAAGCGAGGTCGGAATGTTACTGTGTCTGCCGTGTAGTCTGCGACATCCATGCGGTCAGTCGTCATCAAGGACACAATCCCACCAATGTCAATCTCCTTCCTCTGCAGCAGTCCAACCAACCCTGTGAAGTTGCCGTCTTCCCTCATGTAGCCCCA

>Psic_35410_putative variant iGluR_partial mRNA

GAAGGTTTTCACCATTTATCTCTGAATATTTTGTGTCAAATAAAGTGTCACAAGTAACTGTTTTTTCTTGTCAAGGGAATGATGAGTACCTTCAGCTATCCCAGCGGCTAGTCGCGGCAGGGTTCCTGGTGCAGGCCCATACATCTCCAGTGGCCGGGCAGCTGGCTGCCATCTTGGCAGTTGATTACTACAGGCTGGGTGTTGTCGTGGACATGGACTGCCCTCACATGGCTGCCTTGCTACAAGAGGCTTCGCAACAACGCCTCTTCAGCAGGCTGCACTGGTGGCTGGTGTTGGTGGATGGAGCCTCTGGCCTGCCGCTGGCACTGTCCTCCGAGCCACCCCTGGACAGTCACATGACCTGCGTGCGGGAGAAGGGAGGGCTGTACCACCTGGTGGAGGTGTGGTGTGTGGCGGGCAATCCAGTGACATCCTCTGCAGAGGTTGTGTGGAGACCAGGAGCTGAGATGCCACGGCAGGCACCCAGGGACAACCTCAGTGGGGCCAGCCTCCGTACTGGTATTGTGGCGGTGGAGGATTCCTGGAACCATCGTTCAGATCTGCGCAACAAGCATCTGGACACGTGGAACAAGTTCAGCTACATCCTAGCGACACACGTTGCTCAGATGATGAACTTCCGAATGAACGAAACAGAAGTTGATTCGTGGGGCTACATGAGGGAAGACGGCAACTTCACAGGGTTGGTTGGACTGCTGCAGCGGAAGGAGATTGACATTGGTGGGATTGTGTCCTTG

>Psic_39861_putative variant iGluR_partial mRNA

GGCCCAGCTTGGTAGGCGTCGTCATGATCAGGTAGTGCACCACTTTGGCTCGCTCTTCCGTGTAGAGCACTGCGGTGGAGCCCATGTCTGCCTCGTCCCTCTCCAGGTAGCCGACGATGCCGTCCCACGAGCCGTTCACGAGATATCCCCACGACGGGTCGTGCCGCAGCTCCATGGTAGCATTCATCATTTCGACTGCATGGACAGCCAATGCGTATCCCAATTTGCTTATGGTATCAATGTGCTTATTTTCTAAGTCAGTTAGATGATTTAGTG

>Psic_40191_putative variant iGluR_partial mRNA

TTTGCGCTCTCTTGATGGTGTGGTCTACAACAATATCTACATGTTTACTGCTAGGGAAAAAAAAACCACGCGGACTTCACGTTCTCATATTTCCTCGGTCTCTCGAGAGCACACGTGCCATATATGCTCCAGAGCGATGGCGACGAGGGACAGCGCCATGCCAATGGACAGTATGGCGAACGTGGGCACGACGTACTGCGCGCCGACGCTGACCATCTCCTCTACCGACGAGCACGTCGGCTTCTGCGGCAGCATGTACAGGTACATGTACGATAAGATGCCGGCTTCGCCCACTCGCATCAGCGCACACGTCAGAATTTCCTTCAGCGGGGACTGAAACGGTACAATGATGTATCCTTTGCGCGTCTGATACACGTCCACTTCAGTCAACGAGCACTTCTCCTCGTCAGGAAACGTATCCTCAGATCGGAAGAGCACA

>Psic_49669_putative variant iGluR_partial mRNA

TGGAAAAAATTATAAAAGTTTGATATCCAGGAGTTCCTTTCATCAGGTGTGTCACATGGTGTGGTCAACATCCTAGTAGTCTCTCCAGGCATCCGAAGAAACAAGGAGCCGAAGCTTGTTCTGCGTACCCATGACCTGCACAGTGGTGGCACCATCAGGTTGCTGACATCTTGGCGACGCGGTGGCCTCACGCGGGATGTAAATCTTTTTCCGGACAAGATGCGCCGGGGCTTCAATGGCCAAAGCTTCATAGTGGCAGCCAGTGATCAGCCTCACTATGTAATACACCG

>Psic_50572_putative variant iGluR_partial mRNA

GTCAACGAGCACTTCTCCACGTCAGGGAACGTATCCTCAATCAGGCCGTATATGTTGATGGTCTCGAGGTGAAACGCGAATTCTTCACGAAGAACCTTTCTGACTCCCTCCTCCTGCGGGGAGTAGATCGGTTTTTGGTTGTTCGGTCGCATCATCTTCTTCTGGTACAACTCTTTGACAAGCGGGCTGCTGTCCAGCTCAAAAAATTTATTGATATAGTGATTCTTCTCGGCTCCAAACTGCACTTTACTGTCGATGAGGTCTCTGAGCGTCTTGATGTTGAAAGGAGCTTTCATCAGCAGGGACGATACCACGCATGCGTTGTAGTAGTTGTTCAGAAGTACAGTCAGCACCAGCATCATGAAGAACACCAGGCGCCACGTGATCCAGCGAGAGTTCAGGTCCGTGCCTTTGTTAATAAATAACACCGATATACG

>Psic_51909_putative variant iGluR_partial mRNA

TCTCTGTAGTCGATGTACTTCATGCGGTCATGCACGAACACGAAGGTGGAGATCGCAGTGTCGACTCTGCCCTCGTGCAGCATCTGGACCACGCCGTCGTACGTCCCGCCTTCCACCACCACCCCGAACTTGCTGGTGACCACCATGTCCAGCGTGAAGTTGAACATCTTCTGCAATATGAGGAAGATGTGGTAGTTGTAGACGGCCATTGCATCCGCAAACCTGTTCTCTGCTTTCAATAAGTCCACCTCGGGGTTCTTCAAGGCGGAGTTTAGCACAACGGCAGCCCGCACCGACTGGCCCTGCATGTCCCCACGACGCAGGTACTTGTACCGCTCCAGCAGGTAGCGTAGGCCGGTAGTGGCGCTCCAGCAAGCCGCAGCAGTCTCCAGCACCGGAGCACCCTGCCCGGTGCTGTACAGCTCTGTCACCCGCAGCTCCCCGGTGCTGCCCCTGGAG

>Psic_52085_putative variant iGluR_partial mRNA

GGGATCTTTGAGCAATTTATGTAGCGTTTCTGCTGTGTCAGGTTAGGCAAACGTTGCTCACATTTTTCATACGTTTTTTTCAGCCGGGAAAATATTAATAAACTATAACAATTACTAACTTAAATGTATTATTTAACAAGACATAATTAAAGGATTATGTTGCAGAAGCATACACATGCATACACAAATACAAATATTAATGTAACCGTGGATAGCACGGAAAACAATAATAATTATACCCTTAGAATATAACTATCTGGTAGGGTTTTATACTTTTACATTTATACCAAATCACTGCACTGACAATGAATGCGCTATCGCTGCAAGTCTTCAATGAAAATTCAGTAGAGCAACGTGGATACACGAAAGAAATGGTTCGTGAAACAGAAAAACAACGGATACGTGCACAAATAATTTTCTTTCTTCATGTTCTGTCATTCATGTATTCTTCAGGAGCAGCGCGTCGTTACTCCTCTGCTCTACCCACATACGTCTGTTTTTGAGCGCCATCTCCAGGAGCAGCAACCCGAGCGACAGCAGAACGCCCACAAGAAGGATGGCGAATGCTGGGGTGAGAGCCTCCATGCCGACGGTGGCGACTTCACCAGACGACAGACATTCTGGCTTCCTCAGTTTCCACACCCTGTTGTGATGGTTCAGCAGACCGGTTTCCGCCACTTTCCTCAACCCGTACGTGATGAGCTCCTTGAAGGGCGACCGATGGCGGACGGGCACGAATCACATCTGCGGCGGGAACAGCTCCACCTCGGCGAGGCTGCACTTCTT

>Psic_58358_putative variant iGluR_partial mRNA

GTACCGGTTCCAGTTGCGCTTCTGGAAGCCACGCTCGATGAGCTTGTTGTACGCTATGGCCATGATCTCCTTGTAGACGGAGCCCTTCTGTATGACTTGGTAGGCGTCCACGACCTGTATGTAAGGTATCCTGGTGAGGCCGCACTTCTCGTCCTCGTCGAAGGTGTCCAGCACCAGCTTGTAGCCCGTTCCCACCTCGAAGTGGAAGGCGAACATGCCCTTCCTGATGCGCTCCACGCCGTCGGTCAGCTTCATGTAGCGAGGGGGTACCCCGGGCGGCGCTATTTTCTTCTGGTAGATGGCCCTCCGGATCGGCTCGTCGGCCCTCTCGAAGAAGTATCGGTTGTAGACCATGTCGTGCACCGCCAGCTTCATGCG

>Psic_61434_putative variant iGluR_partial mRNA

GTAGGGCGAGCCCTTCTGCACAGGTATGAGACTGGTAGTGCATGGCGGAAGCATGTACAGCTCCACGAGGTCGCACTTCTCCGCCTCCTGCCAGGTATCCGCGATGAACTTGTATGCGCCTATCTCCACGTGAAAGGCAAACTGTCCCTGCCTCACGAGCTCCATGCCGACCTCCTGCGACAGGTACACCTCGTAGTGCGGCCGTGAGTGGATCCTCTCCCTGAACAGCCTCTGTGCAACAGGCGATGCTGATATGTTCATGTAGTTGTTTATATTTTGGTTGTATATGATGTCATAAAGGGCTATCTTCATCGGACTCTTGACGAGCTCTTCAAGGTTGTTGATGGATGAGCTGGGGGACTGCAGCAGTGACACCACAATGGCAGAGTATGACGAGAAGAAGAAAATGGACAGCAGGTCCAGGCAGAAAAATATCAGTCTGGATGGGAT

>Psic_64649_putative variant iGluR_partial mRNA

ACGCCGAGTAGGACGTGTACACGAACATCACGAATATGAACAGCTGCAGGCTGACGATCCGCCCCGGCGTGCCACGTGACTCCACCGGCGAGCCTTGCTGGCAGAGCGCGCCGACGGACAGCAGGATGACGTCGGTCCAGCCGACCCGCTCGCGGACCCCTGCGGCGAGCCGGTAACCGCGGTCGGCGGCCGTCCTCTCCCAGTGCAGGGCGCCCAGCAGCAGCAGACCGCAGAGGACGAGCAGCGCCGCGCTGCACAGCCACACCGTCCTGCTGAACGGCATCGTGAATACGTTCGACACGGACGACAGCGGCGGCTGCCGGAACACGAACCCCAGCTTGGTGGGCGTGGTCATGACGGCGTAGTCCATGACCTCCTGCCTCTCCCGGTAGAGCAGCATGCCGGCGGCGGCGATGAGCGCGTTGCCTCGCACCACCTCTCCCACCACGCCCGTCCA

>Psic_69026_putative variant iGluR_partial mRNA

TGCGTTTGGATGTTAAGGCGTGTCAGCGAGCATACGTGATGCAGAGGGGGGCAAGCGCCAGGCAGCGTGCCCTGCGCCACAGCAGCTCCATCACAAAAATGGCAATGGAGAAGGCGTACCCATACAGCAGTGCTCCGAGGGCCGGGCCGAATTCTTGCAGCCCGACGCTGACGAAGACAGCGCCCGAGCTGGCGCACGATGGCTTCCCTACTAACCATCGCTTCCTCTCCCTCTCCAAGAGCCCCACCTCACGCAGCCACCGCAGCCTGCCAC

>Psic_72009_putative variant iGluR_partial mRNA

GGGATGCGCGACAGATAGCGGTGTGCACACTCCAGCACCAAGATGGAGGCTGACAGCAGCACACCAAAGGCGAGAACAAGCAGCGCTGGCTGCACATCCTGCAGGCCAGCACTGGTGAAGACAGCACCACGGTCCTGACAGCGAGGACGGTTGGTGCCCCAGCGCACCTTCTCCCTCGCCAGCAGCCCAACCCCACGCACACGTCGTAACTTGAGGGTCAGCATCTTCCTGTAGGGCGAGCCCTTC

>Psic_74229_putative variant iGluR_partial mRNA

GTACGGCTGGATGCTGAGGTGCGTGGTGAAGTTGAGCAACGACCGCCTCCGGTCCGTCGCGGGCAGCGCCACCGCCGCCAGGAACGCCCTCTTCTGCTGCACAGCATCCGCTAAGCTGGTCCATGGGATGGATACGGCCGAGATGTCCGGGGTTTCGCCACCAAACTCATCTACATGCTTGCTTGATGTGTTGCTAGGCTGTCCTTTGGAGGGAGATGAAAGTATTTTCACATGATAACTGAAGTTGAGGTTCCTGGCGAGAGCGGACATGACCTCGAAGACGACGCCGTGGAAGGTGAGGTTCC

>Psic_79628_putative variant iGluR_partial mRNA

CTGTCACCCGCAGCTCCCCGGCGCTGCCCCTGGAGGCCCACGCCACCTCGCTGTCCAGCCGCAGACCCCGGCGGAGGACGGCGTGGCGGGCAGCCGGGTCCTCCACCTCCGACCACACCAGCCAGTGGTAGGTACCATTCAGCAGGCCATTCTCTGAGCTCTTCTGCAGCAGACGTCTGCCGCTGGCGCAGTCGTGGTCCAGGAAGACACCCTGCCGGGTGAACTGCTGGGGCGGCGCCGAACCTAGCGAGCGTGGCGTCAGCACCCACCCGGGGGG

>Psic_83061_putative variant iGluR_partial mRNA

CACAGGCAGATAGCAGACCTCGATGGGGATGATTTGTATTTCGGGGAGGCTGCACTTCTTCTGGTCTGGGAACATTGTCTCTATGATGGGGTACATGTTGTTGGGCTCACTGTGGAAGGCGAACCTCTCGTGCAGAACTCTGCGAATGCCCTCCTCACGACTGAGGAACTCGGGCTTGGTGCGGTGTTGCCCCAACATCTTCTTCACGTACAGTTCGTGCACCAGTGGGTC

>Psic_83400_putative variant iGluR_partial mRNA

ACCCTGGTGCAGATAGAATAATTCTGGTAGGTGTGGCAGCAATGAGCTGGCGATCTGTTGGTGTGTACTCACGCCGGCGACCATGTCGCCGACGACGCCGTCCCAGGAGCCGTCGGGCCGGCGGACGCCGAGGCCGCCTCCTCGCGGCTCCACCAGCTCGTAGTCGAAGCGCAGGCCGTCCGCCAGCTCCTGCAGCAGGTCGACGCAGTAGCCTTCCCAGCGCGGCCGGCC
